# RRAD-reduction reveals efficacy of targeting L-type calcium channel regulation for treatment of heart failure

**DOI:** 10.1093/cvr/cvaf169

**Published:** 2025-10-01

**Authors:** Garrett Elmore, Sarisha S Lohano, Nicholas M McVay, Bryana M Levitan, Andrea Sebastian, Kyle W Barker, Alec Dupont, Steven W Leung, Riham R E Abouleisa, Pretty R Mathew, Austin Wellette-Hunsucker, Austin T Minton, Kenneth S Campbell, Solomon W Harrar, Mohammad Mehri, Jonathan F Wenk, Tamer M A Mohamed, Douglas A Andres, Jonathan Satin

**Affiliations:** Department of Physiology, University of Kentucky, 741 S. Limestone, Lexington, KY 40536-0298, USA; Department of Physiology, University of Kentucky, 741 S. Limestone, Lexington, KY 40536-0298, USA; Department of Physiology, University of Kentucky, 741 S. Limestone, Lexington, KY 40536-0298, USA; Department of Physiology, University of Kentucky, 741 S. Limestone, Lexington, KY 40536-0298, USA; Gill Heart and Vascular Institute, University of Kentucky, Lexington, KY 40506, USA; Department of Physiology, University of Kentucky, 741 S. Limestone, Lexington, KY 40536-0298, USA; Department of Physiology, University of Kentucky, 741 S. Limestone, Lexington, KY 40536-0298, USA; Department of Physiology, University of Kentucky, 741 S. Limestone, Lexington, KY 40536-0298, USA; Gill Heart and Vascular Institute, University of Kentucky, Lexington, KY 40506, USA; Division of Cardiothoracic Surgery, Department of Surgery, Baylor College of Medicine, Houston, TX 77030, USA; Division of Cardiothoracic Surgery, Department of Surgery, Baylor College of Medicine, Houston, TX 77030, USA; Department of Physiology, University of Kentucky, 741 S. Limestone, Lexington, KY 40536-0298, USA; Department of Physiology, University of Kentucky, 741 S. Limestone, Lexington, KY 40536-0298, USA; Department of Physiology, University of Kentucky, 741 S. Limestone, Lexington, KY 40536-0298, USA; Department of Medicine/Cardiology, University of Kentucky HealthCare, Lexington, KY 40506, USA; Department of Statistics, University of Kentucky, Lexington, KY 40506, USA; Department of Mechanical and Aerospace Engineering, University of Kentucky, Lexington, KY 40506, USA; Department of Mechanical and Aerospace Engineering, University of Kentucky, Lexington, KY 40506, USA; Division of Cardiothoracic Surgery, Department of Surgery, Baylor College of Medicine, Houston, TX 77030, USA; Department of Biochemistry, University of Kentucky, Lexington, KY 40506, USA; Department of Physiology, University of Kentucky, 741 S. Limestone, Lexington, KY 40536-0298, USA

**Keywords:** Dilated cardiomyopathy, L-type calcium channel, Therapeutic strategy, Heart failure

## Abstract

**Aims:**

Heart failure with reduced ejection fraction (HFrEF) is a major health problem. Increasing L-type calcium channel (LTCC) activity deteriorates heart function; however, myocardial RRAD knockout (cRAD^Δ/Δ^) instills tonic modulated LTCC current (I_Ca,L_) that preserves healthy myocardium. Thus, we chose to challenge the dogma that enhanced trigger Ca^2+^ is maladaptive. The study objective was to test the hypothesis that modulated I_Ca,L_ in cRAD^Δ/Δ^ mice rescues dilated cardiomyopathy by providing tonic modulated trigger Ca^2+^.

**Methods and results:**

Mouse and human models were tested. The muscle lim protein knockout mouse (MLPKO) is a murine model of dilated cardiomyopathy (DCM) and HFrEF. The experimental timeline was to induce cRAD^Δ/Δ^ after onset of DCM (2.5 months of age) and follow subjects for up to 1-year. Longitudinal echocardiography and cardiac magnetic resonance imaging (CMR) showed that cRAD^Δ/Δ^ intervention rescued systolic function. Patch clamp recordings of isolated cardiomyocytes of MLPKO with cRAD^Δ/Δ^ demonstrated augmented LTCC activity, along with rescue of dysfunctional Ca^2+^ handling and sarcomere function. Bulk RNAseq of hearts demonstrated down-regulated pathological signalling cascades and pro-hypertrophic gene expression which comported with the reduction in eccentric hypertrophy observed with gravimetrics, CMR, and echocardiography. *RRAD* knockdown effects translate from mouse to human heart. Ventricle slices from HFrEF patients were treated with lentiviral shRNA targeting *RRAD* and recapitulated the inotropic and lusitropic effects observed in the mouse model of DCM.

**Conclusion:**

Induction of cardiomyocyte-restricted RAD knockout in MLPKO mice after onset of DCM rescued cardiac dysfunction and attenuated pathological remodelling. cRAD^Δ/Δ^ intervention provided positive inotropy and lusitropy and reverted transcriptional signatures towards healthy myocardium. This study introduces targeting myocardial RAD regulation of the LTCC as a novel therapeutic strategy for systolic heart failure.

## Graphical Abstract

**Graphical Abstract:**
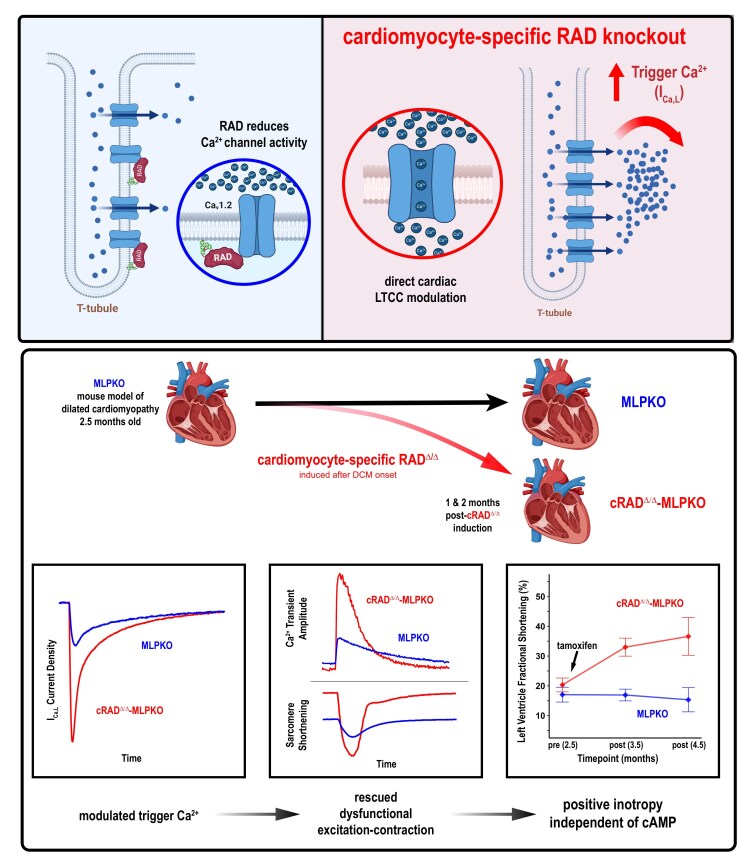

**Time of primary review: 39 days**

**See the editorial comment for this article ‘A RADical approach to treating heart failure’, by M. Obeidat and D.A. Eisner, https://doi.org/10.1093/cvr/cvaf184.**

## 1. Introduction

Heart failure (HF) is a major global health problem,^1^ and heart failure with reduced ejection fraction (HFrEF) represents 50% of HF.^2^ Current therapies fail to adequately address a principal issue: loss of contractile force.^3,4^ Myocardial calcium is a major contributor to cardiac inotropy and targeting calcium dysregulation in HF is an active area of investigation with targets including RyR2, NCX, SERCA, and TRP channels.^5^ LTCC hyperactivity has dogmatically been considered contributory to pathological signalling. Increased I_Ca,L_ by elevated pore-forming Ca_V_1.2^6^ or CaVβ2^7^ expression accelerates heart disease. In stark contrast, cardiomyocyte-restricted induced Rad knockout (cRad^Δ/Δ^) modulates I_Ca,L_ without onset of pathological signalling.^8^ Modulated I_Ca,L_ is defined by greater peak current with faster decay kinetics and lower voltage activation leading to increased trigger Ca^2+^. We reasoned that the increased trigger Ca^2+^ provided by RAD reduction provides an inotropic boost to ameliorate heart failure. This suggests the paradigm-shifting hypothesis that boosted LTCC, specifically LTCC modulation is an efficacious target for treating HFrEF.

Rad is an essential contributor to LTCC localization and modulation in cardiomyocytes.^9^ In the cRad^Δ/Δ^ mouse, I_Ca,L_ under basal conditions phenocopies protein kinase A (PKA) modulated LTCC.^10^ This provides a boost of trigger Ca^2+^ but without sympathetic adrenergic stimulation, which drives cardiac hypertrophy.^11^ When myocardial Rad knockout is induced in the adult there is no resulting hypertrophy or elevation of indices of pathological remodelling,^8^ and adult cardiomyocyte-restricted knockout is phenotypically distinct from the global constitutive Rad knockout which shows a propensity for elevated hypertrophic signalling.^12,13^ Rad is a portmanteau for Ras-associated with diabetes; however, evidence has failed to mechanistically link Rad to diabetes or even show GTPase activity as expected for a ras-related proteins. Rad is a LTCC Ca_V_β2 interacting protein. In over-expression paradigms Rad inhibits I_Ca,L_.^9,14^ Upon β-AR stimulation Rad inhibition of I_Ca,L_ is relieved by a loss of LTCC—Rad association^15^ confirming earlier findings showing that myocardial Rad knockout phenocopies β-AR stimulated I_Ca,L_—importantly, however, under basal conditions.^16^ In addition, β-AR signalling to non-LTCC targets is preserved in cRad^Δ/Δ^.^8^

Here we introduce the LTCC as a target for managing heart failure progression. We used the CSRP3 (commonly called muscle lim protein, MLP) knockout mouse^17^ to test the hypothesis that Rad^Δ/Δ^-modulated LTCC provides acute inotropic support and long-term heart remodelling to prevent and reverse heart failure. MLPKO mice serve as a robust model for studying human dilated cardiomyopathy (DCM) in that they share ventricular dilatation and wall thinning, myocyte hypertrophy, fibrosis, myofilament disarray, impaired contractility, relaxation, cardiac output, and SR Ca^2+^ cycling, prolonged ventricular action potential duration, compromised repolarization, and increased foetal gene expression.^17–23^ MLP is located at Z-discs in sarcomeres of striated muscle and nuclei, and thought to be involved in sensing and relaying muscle stretch.^20,24–26^ We chose the MLPKO model because it has been well-characterized over two decades of study and reliably produces a consistent systolic deficiency. A key focus of this study is evaluating the ability of RAD deletion to act as a calcitrope^27^ after the onset of DCM. Our results suggest that RAD reduction is an effective intervention to treat established DCM.

## 2. Methods

An expanded Methods section is available in the Supplemental Material. All procedures for experiments using animals were approved by the Animal Care and Use Committee of the University of Kentucky and conformed to the National Institute of Health ‘Guide for the Care and Use of Laboratory Animals.’ For echocardiography, Mice were first anesthetized with 2% isoflurane in an induction chamber, chest hair was removed prior to imaging, then a nose cone was placed with inhaled isoflurane, 0.5–1% + 0.5–1.0 L/min 100% O_2_) to maintain a light anaesthesia level, with heart rate (350–500 beats per minute) and core temperature (37°C) continuously monitored and maintained by a heated platform. For euthanasia, ketamine + xylazine (120 mg/kg + 10 mg/kg, ip) are administered and upon loss of toe pinch reflex, cervical luxation is then performed prior to heart harvest. Researchers were blinded to genotype during experiments and subsequent analysis. Human hearts were obtained through the United Network for Organ Sharing (UNOS), Baylor College of Medicine (Institutional Review Board H-53940) or the University of Kentucky (46103). Appropriate consents consistent with the Declaration of Helsinki were obtained in all cases. Some of the specimens were procured from organ donors when the native heart could not be used for transplant.

## 3. Results

### 3.1 RAD knockout rescues cardiac dysfunction and reverse remodels hearts with dilated cardiomyopathy

**Figure 1:**
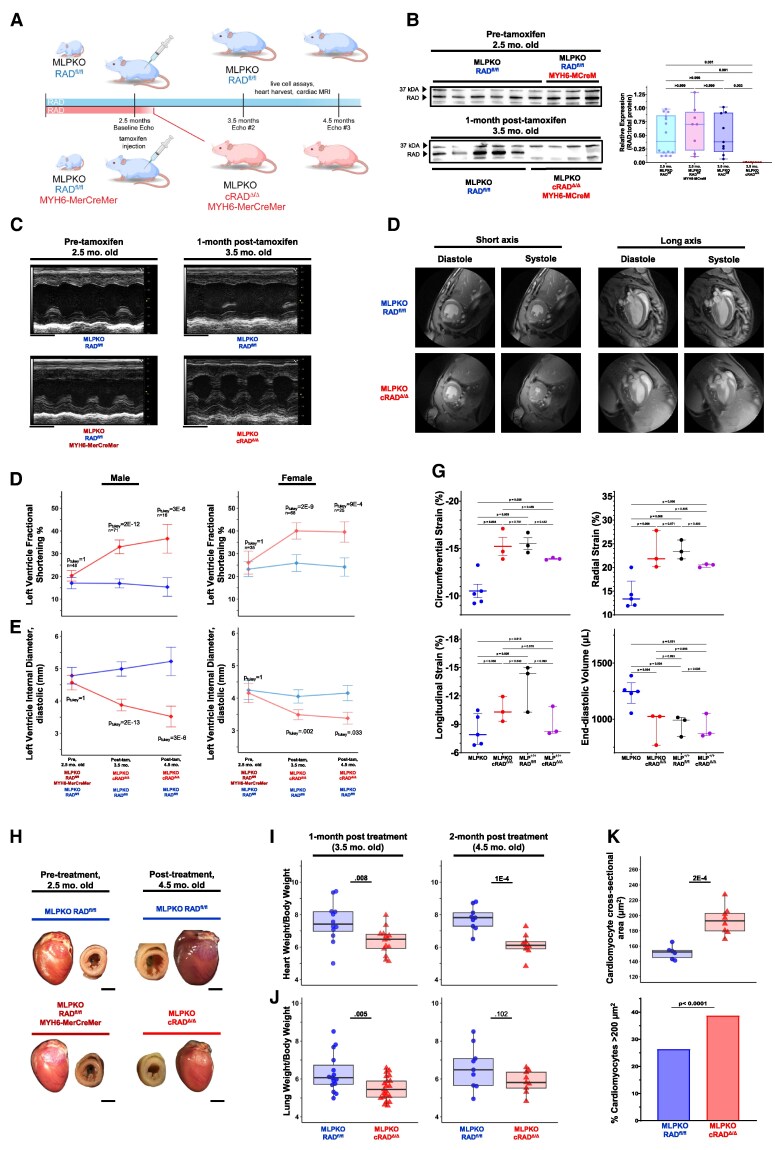
Cardiomyocyte-specific RAD deletion rescues cardiac dysfunction and pathological structural remodelling in DCM. (*A*) Experimental design. MLPKO-RAD^fl/fl^ or MLPKO-RAD^fl/fl^ MYH6-MerCreMer treated with tamoxifen at 2.5-months to yield MLPKO or cRad^Δ/Δ^-MLPKO, respectively. Longitudinal echocardiography on pre-tamoxifen (2.5 month old) and 1- and 2-months post-tamoxifen (3.5- and 4.5-month-old) mice. Live cell assays, heart harvests, and cardiac MRI were performed on mice between or at 1- to 2-months post-tamoxifen. (*B*) Representative western blot for RAD protein from snap-frozen mouse hearts before and after tamoxifen and summary data presented as relative expression of RAD normalized to total protein. cRAD^Δ/Δ^-MLPKO significantly reduced RAD protein expression Kruskal–Wallis (*P* < 0.001) and *post hoc* Dunn’s multiple comparisons test. *N* = 14, 8, 9, and 8 mice (respectively, left to right). (*C*) Representative M-mode echocardiographic tracings pre-tamoxifen and 1-month after tamoxifen administration. The x-axis is seconds (scale bar = 1 s) and y-axis is millimetres. Male mice are represented here; female representatives are shown in Supplementary material online, *Figure S1*. (*D*) Mean left ventricular fractional shortening before and 1- and 2-months after cRAD^Δ/Δ^ induction. cRAD^Δ/Δ^ had a significant treatment effect (genotype*time point, *P* = 2.3E-7, *F* = 16.71) on fractional shortening (linear mixed model accounting for genotype [*P* = 3.7E-17, *F* = 88.17], and time point [repeated measures] [*P* = 3.6E-10, F = 24.66] as fixed factors. The sex fixed factor was significant (*P* = 5.8E-7, *F* = 26.98). The means of male (left, darker colours) pre-tamoxifen MLPKO RAD^fl/fl^ [17.0% (95% CI 14.6, 19.5), *N* = 27] and MLPKO RAD^fl/fl^ MYH6-MerCreMer [20.3%, (18.0, 22.6), *N* = 21] were not significantly different. The means of 1- and 2-months post-tamoxifen male cRAD^Δ/Δ^-MLPKO (33.0% [30.0, 36.0], *N* = 37 and 36.6% [30.3, 42.9], *N* = 8) were significantly greater than MLPKO (16.9% [15.0, 18.9], *N* = 34 and 15.3% [11.3, 19.4], *N* = 8). The means of female (right, lighter colours) pre-tamoxifen MLPKO [23.2% (95% CI 19.9, 26.5), *N* = 14] and MLPKO RAD^fl/fl^ MYH6-MerCreMer [26.1%, (21.0, 31.1), *N* = 21] were not significantly different. The means of female 1- and 2-months post-tamoxifen cRAD^Δ/Δ^-MLPKO (40.0% [36.5, 43.6], *N* = 37 and 39.5% [35.1, 44.0], *N* = 16) were 55 and 64% greater than MLPKO (25.9% [22.2, 29.6], *N* = 31 and 24.2% [20.1, 28.2], *N* = 9). (*E*) Mean left ventricular internal diameter; diastolic. cRAD^Δ/Δ^ had a significant treatment effect (genotype*time point, *P* = 9E-7, *F* = 15.26) on chamber dimension (linear mixed model accounting for genotype [*P* = 1E-12, *F* = 58.88], and time point [repeated measures] [*P* = 2E-6, *F* = 14.20] as fixed factors. The sex fixed factor was significant (*P* = 2E-9, *F* = 40.34). The means of male pre-tamoxifen MLPKO RAD^fl/fl^ [4.78 mm (95% CI 4.53, 5.04), *N* = 27] and MLPKO RAD^fl/fl^ MYH6-MerCreMer [4.57 mm (4.35, 4.79), *N* = 21] were not significantly different. The means of 1- and 2-months post-tamoxifen male cRAD^Δ/Δ^-MLPKO (3.88 mm [3.70, 4.06], *N* = 37 and 3.52 mm [3.20, 3.84], *N* = 8) were significantly greater than MLPKO (4.99 mm [4.77, 5.21], *N* = 34 and 5.23 mm [4.78, 5.67], *N* = 8). The means of female pre-tamoxifen MLPKO RAD^fl/fl^ [4.25 mm (3.96, 4.54), *N* = 14] and MLPKO RAD^fl/fl^ MYH6-MerCreMer [4.16 mm (3.86, 4.46), *N* = 21] were not significantly different. The means of 1- and 2-months post-tamoxifen female cRAD^Δ/Δ^-MLPKO (3.49 mm [3.34, 3.64], *N* = 37 and 3.38 mm [3.20, 3.56], *N* = 16) were significantly 14 and 19% reduced relative to MLPKO (4.05 mm [3.85, 4.26], *N* = 31 and 4.16 mm [3.92, 4.39], *N* = 9). *Post hoc* Tukey tests were used for pairwise comparisons reported for echocardiography time points and are reported in Supplementary material online, *Tables S1*–S3. Significant differences are reported as percent difference of the treatment group increases or decreases relative to the control at the same time point. Individual mouse data are plotted in Supplementary material online, *Figure S1*. (*F*) Cardiac magnetic resonance imaging (CMR) representative diastole and systole short (left) and long-axis views (right) from mice 2-months post-tamoxifen (4.5 months old). (*G*) CMR summary data. Mean circumferential strain of MLPKO was significantly decreased relative to cRAD^Δ/Δ^-MLPKO, MLP^+/+^, and cRAD^Δ/Δ^-MLP^+/+^ [*P* = 0.001; F (DFn 3,DFd10) = 11.9]. Mean radial strain of MLPKO was significantly decreased relative to cRAD^Δ/Δ^-MLPKO, MLP^+/+^, and cRAD^Δ/Δ^-MLP^+/+^ [*P* = 0.003; F (DFn 3,DFd10) = 9.06]. Mean longitudinal strain of MLPKO was significantly decreased relative to cRAD^Δ/Δ^-MLPKO, MLP^+/+^, and cRAD^Δ/Δ^-MLP^+/+^ [*P* = 0.025; F (DFn 3,DFd10) = 4.83]. End diastolic volume of MLPKO was significantly decreased relative to cRAD^Δ/Δ^-MLPKO, MLP^+/+^, and cRAD^Δ/Δ^-MLP^+/+^ [*P* = 0.009; F (DFn 3,DFd10) = 3.67]. Holm–Sidak’s multiple comparison test *P*-values shown on graph. (*H*) Gross intact heart and short-axis views of pre-tamoxifen MLPKO RAD^fl/fl^ and MLPKO RAD^fl/fl^ MYH6-MerCreMer (2.5 months old) and 2-months-post-tamoxifen MLPKO and cRAD^Δ/Δ^-MLPKO (4.5 months old). Scale bars are 3 mm. (*I*) Male heart weights normalized to body weights (mg/g) at 1- and 2-months post-tamoxifen. The means of cRAD^Δ/Δ^-MLPKO (6.36 [95% CI 5.90, 6.83], *N* = 14, and 6.14 [5.62, 6.65], *N* = 9) were significantly less (16%) than MLPKO (7.53 [6.74, 8.32], *N* = 12, and 7.77 [7.21, 8.34], *N* = 9) (independent samples *t*-tests, *t* = 2.89, *P* = 0.008; *t* = 4.95, *P* = 1E-4). (*J*) Male wet lung weights normalized to body weights (mg/g) at 1- and 2-months post-tamoxifen. The means of cRAD^Δ/Δ^-MLPKO (5.50 [5.22, 5.78], *N* = 21, and 5.86 [5.41, 6.31], *N* = 9) were significantly less (21%) than MLPKO (6.31 [5.75, 6.88], *N* = 15, and 6.61 [5.72, 7.50], *N* = 9) (independent samples *t*-tests, *t* = 2.98, *P* = 0.005; *t* = 1.74, *P* = 0.102). (*K*) Mean cardiomyocyte cross-sectional area assessed in wheat germ agglutinin-stained histological short-axis sections was significantly increased in cRAD^Δ/Δ^-MLPKO mice (linear mixed model, cells nested into mice, genotype *F* = 12.733, *P* = 0.001, sex *F* = 15.992, *P* = 5E-4, genotype*sex *F* = 3.953, *P* = 0.058). Mean male area [193.6 µm^2^ (179.7, 207.4)] was increased 28% relative to MLPKO [151.6 µm^2^ (135.6, 167.6)] (*post hoc* Holm *P* = 2.079E-4). Male data are shown; female is shown in Supplementary material online, *Figure S4C–E*. The proportion of cells with area ≥200 µm^2^ was significantly higher in MLPKO cRAD^Δ/Δ^ (39% vs. 26%) than MLPKO (Fisher’s exact test, *P* < 0.0001). *N* = 29 total mice (14 male), 30 technical replicate images per heart from two sections, and 110 060 total cells (52 883 male).

An interventional timeline was chosen to test for Rad reduction to reverse existing dilated cardiomyopathy (DCM). MLPKO-RAD^fl/fl^ and MYH6-MerCreMer-MLPKO-RAD^fl/fl^ mice progressed to a DCM phenotype at 2.5-months, and then all mice were administered tamoxifen to induce cardiomyocyte-restricted RAD deletion in the latter (cRAD^Δ/Δ^; *Figure 1A*). Prior to tamoxifen, RAD protein levels were not different in hearts from 2.5-month MLPKO-RAD^fl/fl^ and MYH6-MerCreMer-MLPKO-RAD^fl/fl^ mice (*Figure 1B*). One month after induction, western blotting showed loss of RAD protein expression in MYH6-MerCreMer-MLPKO-cRAD^Δ/Δ^ hearts (*Figure 1B*). To simplify labelling throughout the paper, the induced MYH6-MerCreMer-MLPKO-RAD^fl/fl^ are abbreviated as cRAD^Δ/Δ^-MLPKO, and MLPKO-RAD^fl/fl^ mice are referred to as MLPKO.

We previously showed in healthy adult mice that cRAD^Δ/Δ^ results in boosted systolic function that is sustained for >1 year.^8^ We reasoned that RAD reduction would also boost systolic function in a model with existing reduced left ventricular fractional shortening (FS). Prior to cRAD^Δ/Δ^ induction, mean FS was about ≤20% for all male mice and was not significantly different across groups (*Figure 1C* and *D* and Supplementary material online, *Figure S1*). There is a significant increase in FS in cRAD^Δ/Δ^-MLPKO mice. There was significant genotype, time point, and sex main effects for FS; moreover, the interaction term (time point*genotype) which represents the treatment effect was significant (*Figure 1D* and Supplementary material online, *Figure S1B*). One-month after tamoxifen, male cRAD^Δ/Δ^-MLPKO mean FS was 95% greater than that of control MLPKO at the same time point. Two-months after tamoxifen, male cRAD^Δ/Δ^-MLPKO was 139% greater. Female cRAD^Δ/Δ^-MLPKO mean fractional shortening was 55 and 64% significantly greater than that of MLPKO at 1- and 2- months after tamoxifen (see Supplementary material online, *Figure S1A* and *B*). Additional longitudinal echocardiography parameters are reported in Supplementary material online, *Table S4*.

We next compared our present interventional cRAD^Δ/Δ^-MLPKO echocardiography results to published studies using the MLPKO mouse, its wild type healthy control, and double transgenic or constitutive knockout mice (see Supplementary material online, *Figure S2* and Supplementary material online, *Table S5*): DWORF-MLPKO,^23^ MYBPC3KO-MLPKO,^21^ βARKct MLPKO,^18^ and AT1aKO MLPKO.^28^ First, we aggregated a recent 2023 study^29^ study reporting disease progression in the MLPKO mouse. One month after tamoxifen treatment, cRAD^Δ/Δ^-MLPKO showed fractional shortening 89% greater (averaging male and female) than the published MLPKO average. cRAD^Δ/Δ^-MLPKO was comparable to published healthy and average protection (e.g. DWORF-MLPKO; Supplementary material online, *Figure S2A*). An important distinction from the present study is that published studies using DWORF, MYBPC3KO, βARKCT, and AT1aKO are constitutive models. In contrast, in the present study we intervened with RAD knockout *after* adult onset of DCM. In summary, DCM hearts recover from compromised function within 4-weeks following RAD reduction.

Heart dimensions were restored by inducing cRAD^Δ/Δ^ on existing DCM. A linear mixed model of the longitudinal echocardiography showed significant genotype, time point, and sex main effects and a significant time point*genotype interaction (*Figure 1E*). Prior to treatment (2.5-months), mean left ventricular internal diameter at end-diastole did not differ (*Figure 1E* and Supplementary material online, *Figure S1C*). One-and-two-months after tamoxifen, male cRAD^Δ/Δ^-MLPKO mean diameter was 22 and 33% less than that of control MLPKO (*Figure 1E* and Supplementary material online, *Figure S1C*). Female cRAD^Δ/Δ^-MLPKO had a mean diameter 14 and 19% less than that of MLPKO 1-month and 2-months after induction (*Figure 1E* and Supplementary material online, *Figure S1C*). The cRAD^Δ/Δ^-MLPKO increase in left ventricular wall to chamber ratio (h/r) suggests significant attenuation of structural remodelling associated with eccentric hypertrophy (see Supplementary material online, Figure1*D*).

We compared our present observations of heart dimensions to published results (see Supplementary material online, *Figure S2B*). Left ventricle dilatation is severe in MLPKO mice—LVID in the present study and literature show a ∼1 mm increase relative to the published control diameter of ∼3.5 mm (see Supplementary material online, *Figure S2B*). As with FS (see Supplementary material online, *Figure S2A*), cRAD^Δ/Δ^-MLPKO returned LV chamber dimension to values comparable to that in health mice (see Supplementary material online, *Figure S2B*).

To further evaluate *in vivo* heart function, we performed cardiac magnetic resonance imaging (CMR). The same timeline was used as for echocardiography—after onset of DCM at 2.5-months tamoxifen was administered to induce RAD knockout. RAD knockout in healthy mice is sympathomimetic for heart function under basal conditions,^10,16^ and *in vivo* this is expected to be reflected as increased strain.^30^ CMR reveals similar structural phenotypes observed from echocardiography (*Figure 1F*) and CMR evaluation was extended to healthy (MLP^+/+^) mice (*Figure 1G*). Hearts in MLPKO mice 2-months after tamoxifen show wall thinning and dilation. In contrast, cRAD^Δ/Δ^-MLPKO show a phenotype of rescue from pathological remodelling (see Supplementary material online, *Videos S1*–S4). cRAD^Δ/Δ^ rescue of MLPKO was not different from that of healthy mice for circumferential, radial, and longitudinal strain, and end diastolic volume (*Figure 1G*).

Gross examination of harvested hearts corroborates echocardiographic and CMR findings (*Figure 1H*); 2.5-month hearts appeared thin-walled with dilated chambers, regardless of genotype. Two-months after tamoxifen MLPKO hearts were further dilated with thin walls. In contrast, cRAD^Δ/Δ^-MLPKO chambers were noticeably smaller with more muscular, thicker walls. Male heart weights (normalized to body weight) were significantly reduced 16 and 21% in cRAD^Δ/Δ^-MLPKO at 1- and 2-months post-tamoxifen, respectively, relative to control MLPKO (*Figure 1I*). Lung weight/body weight is a metric of congestive heart failure. There was a 13% reduction in wet lung weight (normalized to body weight) of cRAD^Δ/Δ^-MLPKO at 1-month post-tamoxifen (*Figure 1J*). Notably, 50% of MLPKO mice had lung weights greater than cRAD^Δ/Δ^-MLPKO’s upper-quartile. Male mice were significantly 39% (∼8 g) larger than female mice, but there was no significant main effect of genotype detected (see Supplementary material online, *Figure S3A* and *B*). Female mean heart and lung weights, normalized to body weight, were not significantly different, although 50% of female MLPKO mice had greater lung weights than cRAD^Δ/Δ^-MLPKO’s upper-quartile as in male (see Supplementary material online, *Figure S4A* and *B*). Histological examination (*Figure 1K* and Supplementary material online, *Figure S5A*) revealed that cRAD^Δ/Δ^-MLPKO ventricular cardiomyocyte short-axis cross-sectional area was significantly increased in males (*Figure 1K*) but not females (see Supplementary material online, *Figure S5B*). Of the >11 000 measured cells, there was a significant sub-population of wider cardiomyocytes in cRAD^Δ/Δ^-MLPKO with area >200 μm^2^ in male (*Figure 1K*) and female (see Supplementary material online, *Figure S5C* and *D*). Fibrosis was not altered by intervening with RAD knockout after onset of DCM (see Supplementary material online, *Figure S6A* and *B*).

We followed mice to 1-year of age. cRAD^Δ/Δ^-MLPKO had 70% increased FS and a 20% reduction in LVID;d relative to MLPKO (see Supplementary material online, *Figure S7A* and *B*). The wall thickness to LVID;d ratio trended similarly to be increased in cRAD^Δ/Δ^-MLPKO (see Supplementary material online, *Figure S7C*). Mouse weight did not vary significantly by genotype, but males were significantly heavier than females as at earlier time points (see Supplementary material online, *Figure S7D*). No deaths were noted in mice in 4.5-month and 1-year cohorts. Taken together these data show that within 4-weeks of induced RAD deletion, heart function and structure revert towards features of healthy hearts, and the treatment effect is preserved at 1 year.

### 3.2 RAD knockout modulates the cardiac L-type calcium channel in ventricular cardiomyocytes from MLPKO hearts

**Figure 2:**
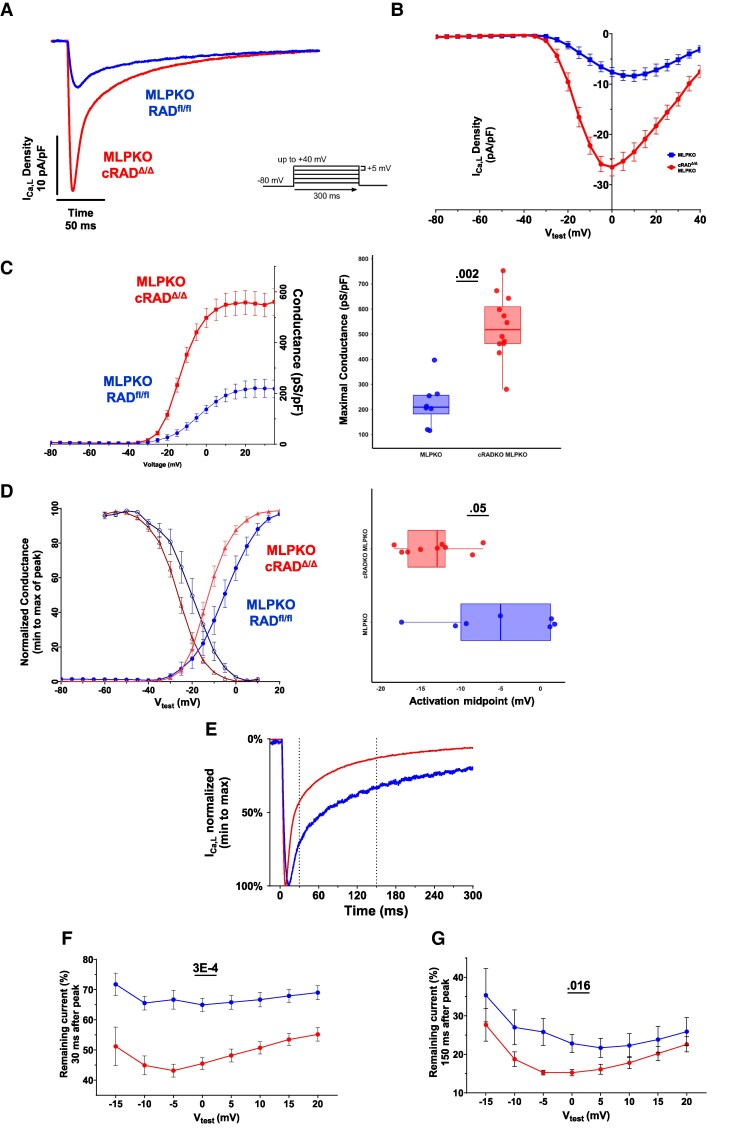
I_Ca,L_ is modulated in cRAD^Δ/δ^-MLPKO cardiomyocytes. (*A*) Representative traces of L-type calcium channel current density of cRAD^Δ/Δ^-MLPKO and MLPKO 1–2 months after tamoxifen (3.5 to 4.5-month-old mice; *V*_hold_ = −80 mV, *V*_test_ = −5 mV). (*B*) Current density-voltage relationship for peak I_Ca,L­_. Voltage protocol schematic (inset left). (*C*) Left panel: Conductance transforms for cRAD^Δ/Δ^-MLPKO and MLPKO. Smooth curves are Boltzmann distributions fitted to the data. Right panel: Mean maximal conductance was significantly increased 2.4-fold in cRAD^Δ/Δ^-MLPKO [531.4 (95% CI 450.9, 612.0)] compared to MLPKO [221.3 (146.9, 295.7)] (linear mixed model, genotype *P* = 0.002, *F* = 23.2). Statistical significance was determined by a linear mixed model, nesting cells into the random factor mouse; however, individual cells are shown in the plot. (*D*) Left panel: Normalized conductance transforms (brighter shades) and steady-state inactivation curves (darker shades) normalized to maximum and minimum conductance, from cRAD^Δ/Δ^-MLPKO and MLPKO. Right panel: Mean activation midpoint was significantly shifted −7.9 mV in cRAD^Δ/Δ^-MLPKO [−13.4 mV (−16.3, −10.4)] relative to MLPKO [−5.4 mV (−12.3, 1.4)] (linear mixed model, genotype *P* = 0.05, *F* = 5.39). (*E*) Representative I_Ca,L_ current recorded for *V*_hold_ = −80 mV stepped to *V*_test_ = −5 mV, normalized to maximum peak current to highlight kinetic differences. (*F*) Percent remaining current 30 ms after peak. cRAD^Δ/Δ^-MLPKO had significantly less remaining fast decaying current (linear mixed model; genotype *P* = 4E-5, *F* = 35.01; voltage *P* = 0.012, *F* = 2.75, genotype × voltage *P* = 0.542, *F* = 0.86). The remaining current was on average reduced 25% in cRAD^Δ/Δ^-MLPKO (49% remaining current) relative to MLPKO (67%). (*G*) Fractional remaining current 150 ms after peak across various test potentials. cRAD^Δ/Δ^-MLPKO had significantly less remaining current (faster decay) (linear mixed model; genotype *P* = 0.012, *F* = 8.20; voltage *P* = 4E-5, *F* = 5.23; interaction *P* = 0.812, *F* = 0.53). The remaining current was on average reduced 24% in cRAD^Δ/Δ^-MLPKO (19% remaining current) relative to MLPKO (26%). MLPKO, *N* = 5 mice, *n* = 8 cells); and cRAD^Δ/Δ^-MLPKO cRAD^Δ/Δ^ (*N* = 5 mice, *n* = 12 cells). Means with SEM shown in conductance transforms and I(V) curve. Box and whisker show cellular data. Individual cell data of kinetic analyses and *post hoc* results are shown in Supplementary material online, *Figure S7*.

Cardiomyocyte specific RAD knockout results in modulated I_Ca,L_.^8,10^ Modulated I_Ca,L_ is defined by increased amplitude, a negative-voltage shifted activation midpoint, and faster current decay. *Figure 2A* shows representative I_Ca,L_ traces from cardiomyocytes isolated from cRAD^Δ/Δ^-MLPKO and MLPKO hearts 1–2 months after tamoxifen (see Supplementary material online, *Figure S8* for families of traces). The I(V) relationship for cRAD^Δ/Δ^-MLPKO shows increased peak current density compared to MLPKO (*Figure 2B*). cRAD^Δ/Δ^-MLPKO showed a > 2.4-fold increase in mean maximal conductance (*Figure 2C*) and a hyperpolarizing shift of activation midpoint (shifted −8 mV) compared to MLPKO (*Figure 2D*). The hyperpolarizing shift of activation midpoint was paralleled by a hyperpolarizing shift in steady-state inactivation of cRAD^Δ/Δ^-MLPKO vs. MLPKO (darker shades, *Figure 2D*). Note the reduction in window current in cRAD^Δ/Δ^-MLPKO vs. MLPKO.

cRAD^Δ/Δ^-MLPKO cardiomyocytes also demonstrated faster decay kinetics of I_Ca,L_; representative traces normalized to maximum current are shown in *Figure 2E*. The early phase of decay (Ca^2+^ dependent inactivation) was assessed by measuring the fractional remaining current 30 milliseconds after peak current. cRAD^Δ/Δ^-MLPKO I_Ca,L_ decay was 25% faster than that of MLPKO cardiomyocytes (*Figure 2F* and Supplementary material online, *Figure S9A*). The late phase of decay (mainly voltage-dependent inactivation) was assessed 150-ms after peak current. Similarly, a significant 24% decrease in remaining current of cRAD^Δ/Δ^-MLPKO was observed (*Figure 2G* and Supplementary material online, *Figure S9B* and *C*). In summary, cRAD^Δ/Δ^ in the MLPKO mouse, recapitulated modulation of I_Ca,L_ under basal conditions. A prominent late I_Ca,L_ was present in MLPKO, but interventional RAD knockout abrogated this potentially arrhythmogenic late phase of I_Ca,L_.^31^

### 3.3 RAD knockout rescues dysfunctional Ca^2+^ handling and sarcomere dynamics in cardiomyocytes from MLPKO hearts

**Figure 3:**
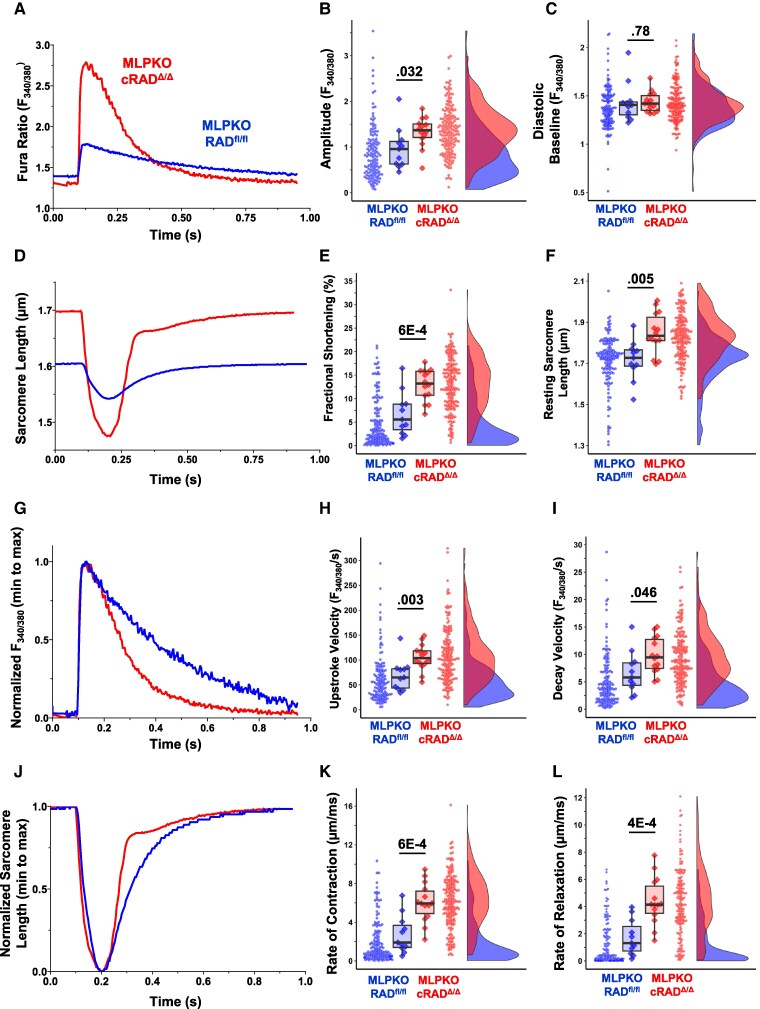
cRAD^Δ/δ^ rescues Ca^2+^ handling and contractility in MLPKO murine cardiomyocytes. (*A*) Representative Ca^2+^ transients recorded in cardiomyocytes cRAD^Δ/Δ^-MLPKO or MLPKO field stimulated at 1 Hz. Right panels show summary data show cells as dots and mice as diamonds, analysed by linear mixed models, nesting cells into mice (biological replicates). (*B*) Mean peak amplitude (Fura-2 ratio_340/380_) was significantly increased 54.1% in cRAD^Δ/Δ^-MLPKO (1.38, 95% CI 1.30–1.46) vs. MLPKO (0.89, 95% CI 0.79–1.00). Genotype as a main effect was significantly different (*P* = 0.032, *F* = 5.35), sex was not (*P* = 0.713, *F* = 0.139), and the interaction was not (*P* = 0.546, *F* = 0.378). (*C*) Mean diastolic baseline of Ca^2+^ transients (*F*_340/380_) were not significantly different between cRAD^Δ/Δ^-MLPKO (1.43, 1.40–1.46) and MLPKO (1.37, 1.34–1.41); genotype (*P* = 0.783, F = 0.078), sex (*P* = 0.488, *F* = 0.501), interaction (*P* = 0.220, *F* = 1.614). (*D*) Representative sarcomere length traces. (*E*) Mean sarcomere fractional shortening was significantly increased 167% in cRAD^Δ/Δ^-MLPKO (13.01%, 12.14–13.89%) vs. MLPKO (4.88%, 4.00–5.75%); genotype (*P* = 5.938e-4, *F* = 16.208), sex (*P* = 0.368, *F* = 0.847), and interaction (*P* = 0.447, *F* = 0.601). (*F*) Mean resting sarcomere length was significantly increased 7% in cRAD^Δ/Δ^-MLPKO (1.83 µm, 1.81–1.85 µm) vs. MLPKO (1.71 µm, 1.69–1.73 µm); genotype (*P* = 0.005, *F* = 9.567), sex (*P* = 0.984, *F* = 4.128e-4), interaction (*P* = 0.847, *F* = 0.038). (*G*) Representative Ca^2+^ transients normalized minimum to maximum to illustrate kinetic differences. (*H*) Mean Ca^2+^ transient maximum upstroke velocity (*F*_340/380_/s) was significantly increased 74.6% in cRAD^Δ/Δ^-MLPKO (108.80, 100.39–117.21) vs. MLPKO (62.33, 54.02–70.63); genotype (*P* = 0.003, *F* = 11.203), sex (*P* = 0.640, *F* = 0.225), and interaction (*P* = 0.930, *F* = 0.008). (*I*) Mean Ca^2+^ transient maximum decay velocity (*F*_340/380_/s) was significantly increased 75.0% in cRAD^Δ/Δ^-MLPKO (9.56, 8.79–10.33) vs. MLPKO (5.46, 4.52–6.41); genotype (*P* = 0.046, *F* = 4.485), sex (*P* = 0.869, *F* = 0.028), and interaction (*P* = 0.546, *F* = 0.376). (*J*) Representative sarcomere length tracings normalized minimum to maximum. (*K*) Mean sarcomere length maximum contraction velocity (µm/s) was significantly increased 192% in cRAD^Δ/Δ^-MLPKO (5.76, 4.86–6.65) vs. MLPKO (1.97, 1.59–2.35); genotype (*P* = 6.095e-4, *F* = 16.332), sex (*P* = 0.828, *F* = 0.049), and interaction (*P* = 0.911, *F* = 0.013). (*L*) Mean sarcomere length maximum relaxation velocity (µm/s) was significantly increased 247% in cRAD^Δ/Δ^-MLPKO (4.12, 3.70–4.55) vs. MLPKO (1.19, 0.92–1.46) genotype (*P* = 3.785e-4, *F* = 17.805), sex (*P* = 0.448, *F* = 0.597), and interaction (*P* = 0.766, *F* = 0.091). For cRAD^Δ/Δ^-MLPKO, *N* = 14 mice, 171 cells; and for MLPKO 11 mice, 132 cells.

Dysfunctional Ca^2+^ cycling and sarcomere function is well-documented in the MLPKO mouse and is a major feature of heart failure.^17,21,23,32^ To test whether modulated trigger I_Ca,L_ in the cRAD^Δ/Δ^-MLPKO heart corresponds with positive inotropy and rescues dysfunction at the cellular level, Ca^2+^ transients and sarcomere dynamics were assessed in isolated cardiomyocytes 1–2 months after tamoxifen (*Figure 3*). Mean Ca^2+^ transient amplitude of cRAD^Δ/Δ^-MLPKO cardiomyocytes increased 54% relative to MLPKO (*Figure 3A* and *B*), while the Ca^2+^ transient diastolic baseline was not significantly different (*Figure 3C*). Sarcomere fractional shortening of cRAD^Δ/Δ^-MLPKO was increased 167%; the MLPKO cellular data were right-skewed, with many cells showing severely compromised contractility (*Figure 3D* and *E*). The resting sarcomere length was significantly more relaxed in cRAD^Δ/Δ^-MLPKO with an average and a greater proportion of cells closer to a longer, healthier resting length of ≥1.8 µm (*Figure 3F*). Kinetics of Ca^2+^ transients and sarcomere contraction were also significantly impacted. cRAD^Δ/Δ^-MLPKO had a 74.6% faster mean upstroke velocity of the Ca^2+^ transient (*Figure 3G* and *H*). Calcium reuptake of cRAD^Δ/Δ^-MLPKO was 75% faster than that of MLPKO (*Figure 3G* and *I*). Similarly, rates of sarcomere contraction and relaxation of cRAD^Δ/Δ^-MLPKO cells were 192 and 247% faster (*Figure 3J–L*). As with contractility, the sarcomere kinetics of MLPKO were extremely skewed towards slower rates, suggesting severe dysfunction.

**Figure 4:**
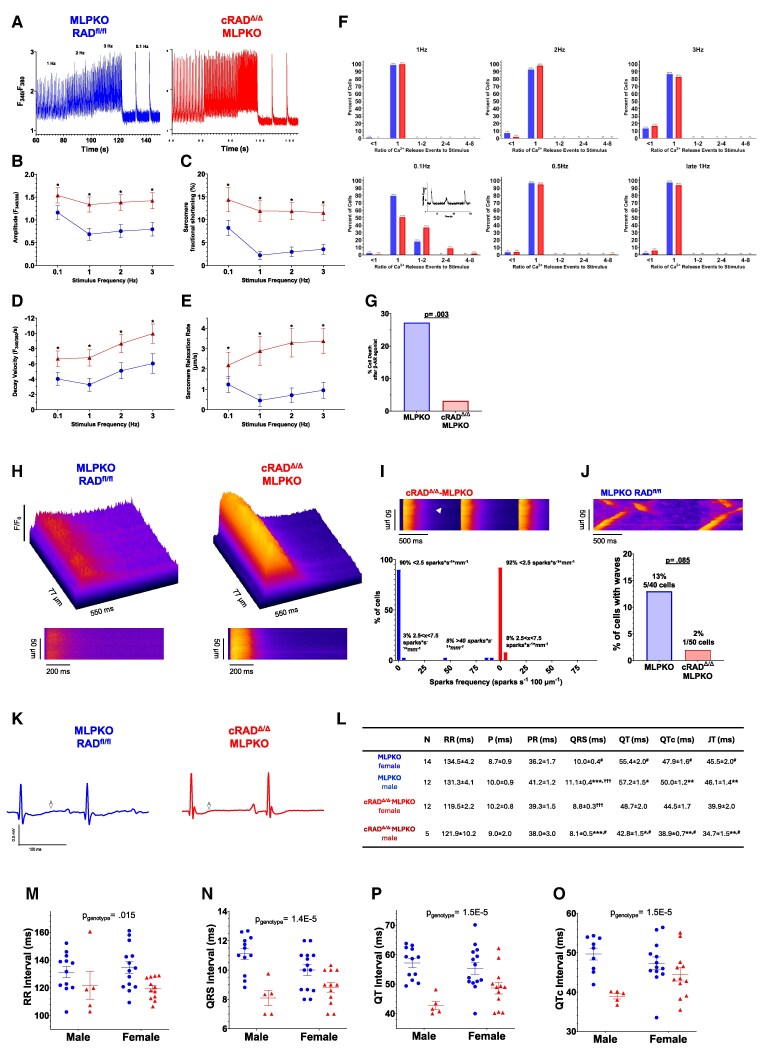
cRAD^Δ/δ^ does not instigate arrhythmogenic diastolic Ca^2+^ release and shortens QTc. (*A*) Representative Ca^2+^ transients recorded in cardiomyocytes from cRAD^Δ/Δ^-MLPKO or MLPKO hearts field stimulated at 1, 2, 3, and 0.1 Hz. (*B*) Mean peak amplitudes of Ca^2+^ transients (*F*_340/380_) were significantly increased 32% (0.1 Hz), 95% (1 Hz), 84% (2 Hz), and 79% (3 Hz) in cRAD^Δ/Δ^-MLPKO vs. MLPKO (mean difference = 0.571, 95% CI 0.357–0.785); genotype (*P* < 0.0001, *F* = 27.99), frequency (*P* < 0.0001, *F* = 110.6), and interaction (*P* < 0.0001, *F* = 21.43). (*C*) Mean maximum decay velocities of Ca^2+^ transients (*F*_340/380_/s) were significantly faster 66% (0.1 Hz), 108% (1 Hz), 70% (2 Hz), and 65% (3 Hz) in cRAD^Δ/Δ^-MLPKO vs. MLPKO (mean difference = 3.413, 95% CI 1.953–4.874); genotype (*P* < 0.0001, *F* = 21.54), frequency (*P* < 0.0001, *F* = 97.22), and interaction (*P* = 0.0131, *F* = 3.652). (*D*) Mean sarcomere fractional shortenings (%) were significantly increased 75% (0.1 Hz), 433% (1 Hz), 299% (2 Hz), and 225% (3 Hz) in cRAD^Δ/Δ^-MLPKO vs. MLPKO (mean difference = 8.173, 95% CI 6.133–10.21); genotype (*P* < 0.0001, F = 63.66), frequency (*P* < 0.0001, *F* = 57.02), and interaction (*P* = 0.0131, *F* = 8.391). (*E*) Mean sarcomere maximum relaxation rates (µm/s) were significantly increased 77% (0.1 Hz), 539% (1 Hz), 363% (2 Hz), and 253% (3 Hz) in cRAD^Δ/Δ^-MLPKO vs. MLPKO (mean difference = 2.094, 95% CI 1.457–2.730); genotype (*P* < 0.0001, *F* = 42.94), frequency (*P* < 0.0001, *F* = 11.36), and interaction (*P* = 0.0131, *F* = 30.16). For MLPKO cRAD^Δ/Δ^, *N* = 3 mice, 39 cells, and 156 observations; MLPKO, *N* = 5 mice, 55 cells, and 220 observations. (*F*) Non-stimulated Ca^2+^ release events for 1, 2, 3, 0.1, and 0.5 Hz sequential stimulation. Late 1 Hz refers to a second 1 Hz stimulation immediately following the 0.5 Hz challenge. Unstimulated Ca^2+^ release was not observed for all frequencies ≥0.5 Hz. Individual cell data are in Supplementary material online, *Figure S9*. (*G*) MLPKO cells showed calcium overload (fura-2 ratio diastolic baseline increased >2-fold and held), hypercontracted, and died significantly three times more than cRAD^Δ/Δ^-MLPKO cells when exposed to 1 µM isoproterenol (stimulated at 1 Hz) (Fisher’s exact test, *P* = 0.003). For cRAD^Δ/Δ^-MLPKO and MLPKO, *N* = 12 and 8 mice, and 64 and 22 cells. (*H*) Representative Ca^2+^ transients and their surface plots recorded from laser scan confocal of cRAD^Δ/Δ^-MLPKO or MLPKO cardiomyocytes, field stimulated at 1 Hz. (*I*) Representative spark (white arrow) in a cRAD^Δ/Δ^-MLPKO cardiomyocyte during 1 Hz pacing. Histogram of spark frequency among cells; 92% and 90% of cRAD^Δ/Δ^-MLPKO or MLPKO cardiomyocytes showed a frequency <2.5 sparks per second per millimetre. (*J*) MLPKO cardiomyocyte showing high frequency of sparks and waves during 1 Hz stimulation. The frequency of cells showing waves between MLPKO (5/40 cells) and cRAD^Δ/Δ^-MLPKO (1/50 cells) was not significantly different (Fisher’s exact test, *P* = 0.085). *N* = 3 mice per group. (*K*) Representative surface electrocardiograms from anesthetized cRAD^Δ/Δ^-MLPKO and MLPKO mice. (*L*) Surface ECG conduction intervals. P and PR did not differ significantly between cRAD^Δ/Δ^-MLPKO and MLPKO mice (two-way ANOVAs, respectively: genotype, *P* = 0.820 and 0.996, *F* = 0.052 and 3E-5; sex = 0.963 and 0.313, *F* = 2E-3 and 1.044; genotype*sex, *P* = 0.253 and 0.087, and *F* = 1.344 and 3.074). RR differed significantly by genotype (*P* = 0.015, *F* = 6.497) but not sex (*P* = 0.934, *F* = 7E-3) (interaction, *P* = 0.569, *F* = 0.330). QRS (*M*) differed significantly by genotype (*P* = 1E-5, *F* = 24.569) but not sex (*P* = 0.656, *F* = 0.201) and the interaction was significant (*P* = 0.037, *F* = 4.676). QT differed significantly by genotype (*P* = 1E-5, *F* = 24.417) but not sex (*P* = 0.341, *F* = 0.930) and the interaction was not significant (*P* = 0.081, *F* = 3.217). QTc (*N*) [using the formula, QTc = QT/sqrt(RR/100)] differed significantly by genotype (*P* = 1E-5, *F* = 17.589) but not sex (*P* = 0.321, *F* = 1.011) and the interaction was significant (*P* = 0.030, *F* = 5.045). JT differed significantly by genotype (*P* = 2E-4, *F* = 16.942) but not sex (*P* = 0.275, *F* = 1.223) and the interaction was not significant (*P* = 0.170, *F* = 1.950). *Post hoc* Tukey results are presented in the table where * denote significance between male MLPKO and cRAD^Δ/Δ^-MLPKO, # between female MLPKO and male cRAD^Δ/Δ^-MLPKO, and ^†^ for male MLPKO and female cRAD^Δ/Δ^-MLPKO. One symbol denotes *P* < 0.05, two *P* < 0.01, and three *P* < 0.001.

We next extended analysis to evaluate Ca^2+^ cycling and sarcomere dynamics over a range of stimulation frequencies (*Figure 4A* and Supplementary material online, *Figure S10*). The cRAD^Δ/Δ^-MLPKO genotype main effect persisted for all frequencies for Ca^2+^ transient amplitude (68% higher) and sarcomere fractional shortening (193% higher) (*Figure 4B* and *C*). Frequency dependent acceleration of relaxation (FDAR) is an adaptive mechanism to support ventricle filling at higher heart rates.^33,34,35^ Ca^2+^ transient decay velocity and sarcomere relaxation velocity were significantly increased 74 and 250% across all frequencies (*Figure 4D* and *E*). Ca^2+^ transient alternans was not present in either genotype. We also analysed the Ca^2+^ transients for unstimulated calcium release events—visualized in *Figure 4F* with histograms of the ratio of Ca^2+^ release events to stimulus events. A ratio of 1 indicates an entrained cell with no unstimulated releases. Neither group exhibited unstimulated release events at stimulation frequencies of 1, 2, or 3 Hz. Unstimulated release occurred in both genotypes for 0.1 Hz.

Increased Ca^2+^ transient reuptake in cRAD^Δ/Δ^-MLPKO cardiomyocytes suggested greater sarcoplasmic reticulum Ca^2+^-store load. Caffeine-releasable Ca^2+^ was significantly elevated in cRAD^Δ/Δ^-MLPKO cardiomyocytes (see Supplementary material online, *Figure S11*). As in healthy cardiomyocytes, the ratio of electrically stimulated cytosolic Ca^2+^ approached (82%) the caffeine-mobilizable SR Ca^2+^-load in cRAD^Δ/Δ^-MLPKO cardiomyocytes. In contrast, MLPKO twitch Ca^2+^ was only 27% of SR Ca^2+^ (see Supplementary material online, *Figure S11*). Taken together, these results suggest that increased trigger Ca^2+^ in cRAD^Δ/Δ^-MLPKO translates to improved Ca^2+^ homeostasis (more release and faster reuptake) and enhanced sarcomere dynamics.

Acute isoproterenol provoked hypercontracture death in 27% of cardiomyocytes from MLPKO compared to only 3% from cRAD^Δ/Δ^-MLPKO hearts (*Figure 4G*). To further evaluate arrhythmogenic vulnerability, we recorded local Ca^2+^ transients. Note the high degree of synchrony in cRAD^Δ/Δ^-MLPKO across the long-axis of the cell (*Figure 4H*, right) and protracted diastolic cytosolic Ca^2+^ (*Figure 4H*, left). Paralleling the lack of unstimulated Ca^2+^ release events at 1 Hz pacing in *Figure 4F*, the majority of MLPKO (90%) and cRAD^Δ/Δ^-MLPKO (92%) cells had no or rare occurrence of Ca^2+^ sparks (<2.5 sparks per second per millimetre during pacing) (*Figure 4I*). 13% of MLPKO cells showed waves and more than half of these cells had a high rate of sparks (>40 sparks per s per mm) (*Figure 4J*).

MLPKO had significant prolongation of RR (13 ms), QRS (2 ms), QT (9 ms), and QTc (6 ms) intervals compared to cRAD^Δ/Δ^-MLPKO (*Figure 4K–O*). For reference, Gardiwal *et al*.^19^ reported that MLPKO relative to healthy MLP^+/+^ controls had significant prolongations of RR (10 ms), QRS (4 ms), QT (21 ms), QTc (19 ms), and JT (17 ms).^19,36^ Consistent with previously published finding that in healthy mice cRadKO reduces QTc,^10^ in DCM rescue, cRAD^Δ/Δ^-MLPKO also enhances repolarization. The absence of elevated Ca^2+^ sparks and resistance to acute ISO hypercontracture along with absence of sudden cardiac death for 1-year suggests that cRadKO may not provoke arrhythmias in mice. Mechanistically cRadKO driving shortened QTc can be explained by faster I_Ca,L_ decay and significantly faster Ca^2+^ reuptake.^31^

### 3.4 In human dilated cardiomyopathy heart slices, RAD knockdown augments contractility and Ca^2+^ handling

**Figure 5:**
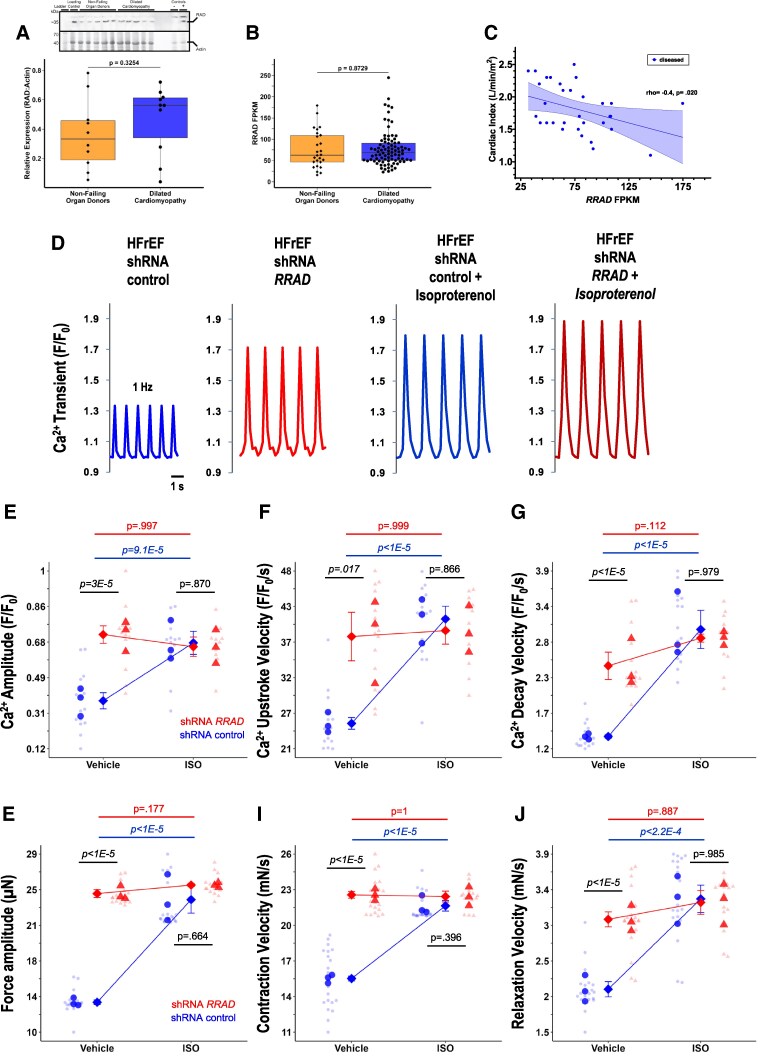
RAD and *RRAD* expression does not differ in human DCM relative to non-failing organ donors, *RRAD* expression is negatively correlated with cardiac index in human DCM patients, and RAD knockdown improves contractility and Ca^2+^ handling in human HFrEF heart. (*A*) Human RAD protein expression in the heart did not differ significantly between non-failing organ donors and donors with HFrEF and dilated cardiomyopathy (*t*-test, *t* = 1.011, *P* = 0.3254). A representative western blot is shown. Negative and positive controls were a cRAD^Δ/Δ^-MLPKO and MLPKO mouse. (*B*) Human *RRAD* transcript expression in the heart did not differ significantly between non-failing organ donors and humans with HFrEF and dilated cardiomyopathy (*t*-test, *t* = 0.1610, *P* = 0.8729). FPKM = fragments per kilobase per million bases. (*C*) Cardiac index (thermodilution method) was negatively correlated with *RRAD* expression in humans with HFrEF and dilated cardiomyopathy (spearman’s, rho = −0.4022, *P* = 0.0203). (*D*) Representative Ca^2+^ transients for shRNA control and shRNA *RRAD* knockdown measured as relative fluorescence of Fluo-4 with or without acute isoproterenol. (*E*) Mean Ca^2+^ transient amplitude (*F*/*F*_0_) was significantly elevated 93% by *RRAD* knockdown (control 0.371 ± 0.042 vs. 0.716 ± 0.046) [mixed model ANOVA, nesting each heart strip into the random factor, patient heart; shRNA treatment *F* = 190.128, *P* = 0.001; drug (isoproterenol) *F* = 7.128, *P* = 0.091; interaction *F* = 12.070, *P* = 0.060]. (*F*) Mean Ca^2+^ transient upstroke velocity (*F*/*F*_0_ · s^−1^) was significantly hastened 53% by *RRAD* knockdown (control 24.9 ± 0.9 vs. 38.3 ± 3.73) [mixed model ANOVA, shRNA treatment *F* = 351.277, *P* = 0.002; drug (isoproterenol) *F* = 7.686, *P* = 0.107; interaction *F* = 10.942, *P* = 0.071]. (*G*) Mean Ca^2+^ transient decay velocity (*F*/*F*_0_ · s^−1^) was significantly hastened 79% by *RRAD* knockdown (control 1.38 ± 0.026 vs. 2.47 ± 0.212) [mixed model ANOVA, shRNA treatment *F* = 163.751, *P* = 0.004; drug (isoproterenol) *F* = 108.573, *P* = 8.8e-4; interaction *F* = 7.174, *P* = 0.114]. (*H*) Force amplitude (µN) was significantly elevated 84% by *RRAD* knockdown (control 13.3 ± 0.24 vs. 24.6 ± 0.41) [mixed model ANOVA, shRNA treatment *F* = 5303.199, *P* = 6.1e-5; drug (isoproterenol) *F* = 62.502, *P* = 0.017; interaction *F* = 26.709, *P* = 0.037]. (*I*) Contraction max velocity (mN/s) was significantly hastened 45% by *RRAD* knockdown (control 15.8 ± 0.20 vs. 22.8 ± 0.28) [mixed model ANOVA, shRNA treatment *F* = 2386.917, *P* = 2.1e-6; drug (isoproterenol) *F* = 51.782, *P* = 0.005; interaction *F* = 71.171, *P* = 4e-11]. (*J*) Relaxation max velocity (mN/s) was significantly quickened 46% by *RRAD* knockdown (control 2.08 ± 0.104 vs. 3.03 ± 0.101) [mixed model ANOVA, shRNA treatment *F* = 251.340, *P* = 0.001; drug (isoproterenol) *F* = 6.703, *P* = 0.119; interaction *F* = 38.451, *P* = 3e-6]. The diamonds show means ± SEM. *N* = 3 biological replicates (hearts) (opaque circles/triangles); technical replicates of heart slices (transparent data points in background) treated with shRNA control (*n* = 21), shRNA *RRAD* (21), shRNA control with isoproterenol (21), shRNA *RRAD* with isoproterenol (16). Tukey’s was used for *post hoc* analysis—*P*-values are reported on the plots. Additional descriptive statistics are in Supplementary material online, *Table S6*.

RAD expression has not been reported in human dilated cardiomyopathy hearts. Therefore, we measured RAD protein and transcript abundance in human patients with non-ischaemic HFrEF and dilated cardiomyopathy in comparison to non-failing organ donors and were unable to detect a significant difference (*Figure 5A* and *B*). Of 58 additional clinical variables collected from diseased patients, 4 variables were significantly correlated with *RRAD* transcript expression: cardiac output (rho = −0.4, *P* = 0.013) and index (rho = −0.4, *P* = 0.020) (*Figure 5C*), end stroke volume (rho = −0.3, *P* = 0.030), and N-terminal pro-B-type natriuretic peptide (NT-proBNP) (rho = 0.3, *P* = 0.025). Thus, while expression levels were not significantly different between non-failing donors and diseased donors, more severely diseased patients with poorer cardiac index and more NT-proBNP had more *RRAD* expression.

To evaluate the translational potential of targeting RAD reduction as a treatment for systolic HF we evaluated the impact of RAD knockdown in *ex vivo* human heart slices from HFrEF patients (*Figure 5D*). *Ex vivo* heart slices were cultured and treated with lentivirus encoding shRNA targeting RAD or scrambled control, which achieved 70–80% knockdown of RAD expression in heart slices (see Supplementary material online, *Figure S12*). Ca^2+^ transients in RAD knockdown *ex vivo* heart slices were elevated 93%, and RAD-knockdown Ca^2+^ transient levels were not significantly different from acute ISO-treated control levels (*Figure 5E*). Similarly, Ca^2+^ upstroke velocity increased 53% in RAD knockdown to levels not significantly different from ISO-treated control levels (*Figure 5F*). RAD reduction accelerated Ca^2+^ transient decay rate under basal conditions by 79% (*Figure 5G*). In parallel, force tracked with Ca^2+^ transients in failing human heart. RNA knockdown increased basal force by 84% (*Figure 5H*), and the kinetics of force development by 45% (*Figure 5I*) and relaxation by 46% (*Figure 5J*). Force levels in basal RAD knockdown were not different from those in control or RAD knockdown with acute ISO challenge (*Figure 5H*). Taken together, these results show that positive inotropic functional effects of RAD reduction in failing heart translate from mouse to human heart.

### 3.5 RAD knockout rescue of DCM attenuates pathological remodelling signalling

**Figure 6:**
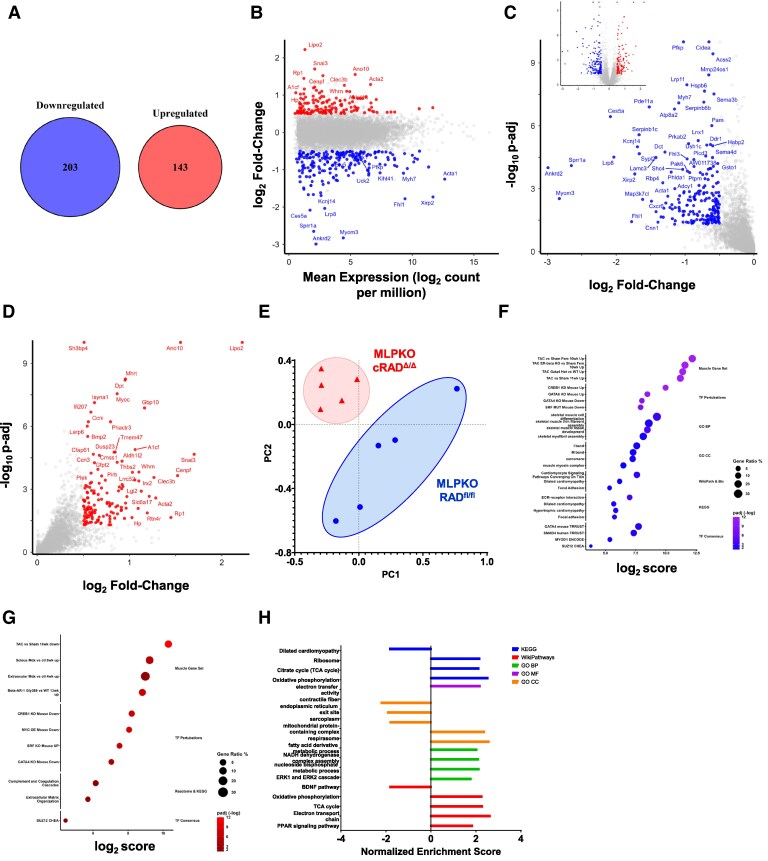
cRAD^Δ/δ^-MLPKO attenuates gene expression changes associated with pathological remodelling in the MLPKO mouse. Bulk RNAseq results of hearts from five MLPKO and five cRAD^Δ/Δ^-MLPKO 3.5-month-old male mice (1 month after cRAD^Δ/Δ^ induction). (*A*) DESeq2 revealed 346 differentially expressed (DE) genes (0.5 log_2_ fold-change and *P*-adj < 0.05). Two-hundred three genes were down-regulated and 143 up-regulated in cRAD^Δ/Δ^-MLPKO relative to MLPKO. (*B*) Bland–Altman plot of down-regulated (blue) and up-regulated (red) DE genes. Volcano plots of down-regulated (*C*) and up-regulated (*D*) DE genes. (*E*) Principal component analysis—each point represents a mouse. Singular enrichment analysis using the down-regulated (*F*) and up-regulated (*G*) differentially expressed genes, that included experimental model databases (Muscle Gene Set, TF Pertubations), gene ontology (GO Biological Processes and Cellular Compartments), transcription factors (TF Consensus of ENCODE & CHEA, and TRRUST), and pathway databases (Wiki Pathways, BioPlanet, KEGG). All terms shown have *P*-adj <0.05. (*H*) Gene set enrichment analysis on genes ranked by log_2_ fold change. Only significant (*P*-adj <0.05) terms are included and the highest scoring terms within the five databases are shown (KEGG, Wiki Pathways, GO biological processes, GO molecular functions, and GO cellular compartments).

Bulk RNA-seq analysis of left ventricle myocardium from 3.5-month-old mice comparing MLPKO to cRAD^Δ/Δ^-MLPKO showed gene expression changes consistent with attenuation of pathological remodelling signalling caused by RAD knockout (*Figure 6A*). There were 1327 genes whose expression was significantly different (adjusted *P*-value <0.05). 346 transcripts were differentially expressed (0.5 log_2_ fold-change; *Figure 6A–D*). Principle component analysis clustered cRAD^Δ/Δ^-MLPKO consistent with a similar rescue transcriptome profile (*Figure 6E*). Singular enrichment analysis (SEA) was performed on the down-regulated (*Figure 6F*) and up-regulated (*Figure 6G*) differentially expressed genes using Enrichr.^37–39^ The Muscle Gene Set database^40^ (which includes mouse models of heart failure) enriched terms suggest that cRAD^Δ/Δ^-MLPKO transcriptional profile was inversely correlated with disease models and positively correlated with healthy controls. Genes normally up-regulated in heart failure, such as foetal gene programme genes, were down-regulated in cRAD^Δ/Δ−^MLPKO and vice versa, suggesting that RAD knockout attenuated pathological remodelling signalling cascades. Moreover, the Transcription Factors Perturbations^39^ database shows inverse correlation of genes associated with perturbation of transcription factors known to be highly linked to pathological remodelling and the foetal gene programme, such as GATA4, SRF, and CREB1. GATA4 is an upstream regulator of the foetal gene programme and pro-hypertrophic genes and was the highest scoring transcription factor identified among the transcriptional regulatory network databases, TRRUST, ENCODE, and CHEA.^39,41–44^ The Gene Ontology^45,46^ databases contained terms enriched with genes involved in sarcomere and striated muscle. The significant halving of sarcomere genes associated with disease *Ankrd1*, *Ankrd2*, *Acta1*, *Myh7*, *Myom2*, and *Myom3* expression in cRAD^Δ/Δ^-MLPKO are major contributors to the enriched GO terms regarding amelioration of sarcomere hypertrophy in cRAD^Δ/Δ−^MLPKO.

Pathway databases (WikiPathways,^47^ BioPlanet,^48^ KEGG,^49,50^ and Reactome^51^) enriched terms were related to cardiomyopathy, inflammatory response, focal adhesion, and the extracellular matrix. Terms related to cardiomyopathy are enriched in genes of the foetal gene programme (*Acta1* and *Myh7*) and sarcomere genes. Focal adhesion and the ECM-receptor interaction terms were enriched with integrin, laminin, filamin C, collagen, and other activated-myofibroblasts associated genes. Accordingly, metalloproteinases (e.g. *Adamts8, Mmp23*) and serpin family genes (e.g. *Serpine1, Serpinb1c*)—matrisome genes associated with extracellular matrix organization and fibrosis—were significantly halved in cRAD^Δ/Δ^-MLPKO. A transcription factor involved in fibroblast signalling cascades upstream of these genes, SMAD4, was an enriched term in the TRRUST database. Together, the ECM organization, sarcomere hypertrophy, foetal gene programme terms constitute many of the up-regulated genes associated with disease models in the Muscle Gene Set and Transcription Factor Perturbation databases; thus, down-regulation of these genes in cRAD^Δ/Δ^-MLPKO suggests attenuation of pathological remodelling signalling.

Gene set enrichment analysis (GSEA) utilizes a ranked gene list of all genes not dependent on user thresholds for input and was performed using WebGestalt^52^ (*Figure 6H*). Oxidative phosphorylation and fatty acid oxidation were down-regulated in MLPKO relative to cRAD^Δ/Δ^-MLPKO, corroborating the SEA results showing that cRAD^Δ/Δ^-MLPKO has less induction of the foetal gene programme. Failing myocardium includes post-natal fatty acid oxidation down-regulation and up-regulated glycolysis and carbohydrate fuel usage; relatedly, the PPAR signalling pathway was up-regulated in cRAD^Δ/Δ^-MLPKO, indicating mitochondrial biogenesis and fatty acid substrate usage.^53^

**Figure 7:**
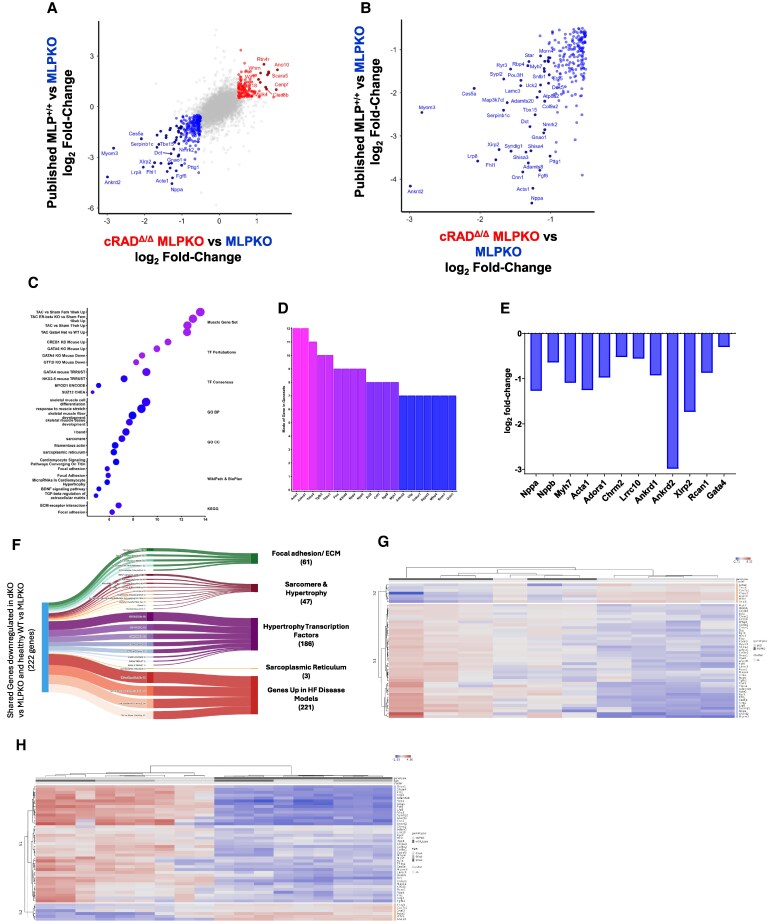
cRAD^Δ/δ^-MLPKO exhibits a transcriptomic profile that trends towards healthy MLP^+/+^ vs. MLPKO. (*A*) Correlation of differentially expressed genes between published MLP^+/+^ vs. MLPKO^31^ and the present study of cRAD^Δ/Δ^-MLPKO vs. MLPKO. Blue dots demark log_2_ fold-change < −0.5 and dark blue, log_2_ FC < −1. Red dots demark log_2_ FC > 0.5 and dark red, log_2_ FC > 1. (*B*) A zoomed-in section of A, focusing on the 222 shared down-regulated genes (log_2_ fold-change < −0.5). (*C*) Singular enrichment analysis (SEA) of shared down-regulated genes (log_2_ FC < −0.5). (*D*) Genes that appeared the most in enriched SEA terms. (*E*) Foetal gene programme, sarcomere hypertrophy, and GATA4 transcription factor target genes expression was down-regulated in cRAD^Δ/Δ^-MLPKO vs. MLPKO. (*F*) Alluvial diagram showing the categories of SEA annotations of the 222 down-regulated genes of published healthy shared between MLP^+/+^ and cRAD^Δ/Δ^-MLPKO vs. MLPKO. The middle column shows the SEA terms (from *C*) and number of genes constituting them, along with curated annotation in the rightmost column. (*G*) Top genes representing each category or typically screened for in other MLPKO and HF disease models (e.g. foetal gene programme) were subjected to unsupervised hierarchical clustering in the cRAD^Δ/Δ^-MLPKO and MLPKO datasets. (*H*) Unsupervised hierarchical clustering algorithm was performed using the same genes as in G on published MLP^+/+^ and MLPKO time series (3, 6, and 10 week) data (Holmes *et al*. 2023). Each row is a gene, and each column represents a mouse, with values being z-scores.

We next evaluated our findings comparing RAD-reduction rescued DCM in MLPKO to a recent bulk RNA-seq analysis of 3, 6, and 10 weeks of age of MLPKO in comparison to WT mice (MLP^+/+^) (*Figure 7*).^29^ Holmes *et al*. reported that mice at 3-weeks had not developed significant systolic dysfunction or dilation but demonstrated diastolic dysfunction; however, the transcriptomic profiles of 3-week-old MLPKO vs. MLP^+/+^ mice already showed hallmarks of heart failure and disease models including up-regulation of sarcomere hypertrophy, inflammatory response, extracellular matrix pathways, and activated-fibroblast related pathways. By 6 and 10 weeks, Holmes *et al*. MLPKO^29^ showed significant systolic function (LV FS% 22 vs. 39; 16 vs. 36%) and LV dilation (3.8 vs. 3.4; 4.2 vs. 3.4 mm) vs. MLP^+/+^, and the up-regulation of these pathways became more severe, along with the two other typical HF alterations: down-regulation of fatty acid oxidation and up-regulation of apoptosis. To analyse the correlation between cRAD^Δ/Δ^ effects on MLPKO (our new data) and MLP^+/+^ vs. MLPKO,^29^ the top correlated genes in each experiment were further analysed (*Figure 7A* and *B*). Singular enrichment analysis of correlated down-regulated genes demonstrated significant down-regulation of HF hallmarks in cRAD^Δ/Δ^-MLPKO (*Figure 7C*). GATA4 was again identified as the highest scoring transcription factor. The genes with the highest mode in the singular enrichment analysis (*Figure 7D*) and highest magnitude of down-regulation (see Supplementary material online, *Figure S13*), consisted of foetal cardiac and sarcomere hypertrophy genes (*Acta1, Ankrd1, Nppa, Nppb, Myh7,* and *Ankrd2*), and activated-fibroblast genes. *Figure 7E* highlights the down-regulation of foetal gene programme and sarcomere hypertrophy genes in the myocardium of cRAD^Δ/Δ^-MLPKO indicative of pro-hypertrophic gene expression that have been reported up-regulated in disease models such as MLPKO. These genes along with *Adora1 and Chrm2,* are linked to GATA4, SRF, and NKX2-5 transcription factor activity.

The 222 genes that shared down-regulation in cRAD^Δ/Δ^-MLPKO and published MLP^+/+^ vs. MLPKO (*Figure 7B*) and the resulting enrichment terms (*Figure 7C*) were categorized into 5 groups: mechanical sensing of focal adhesion/extracellular matrix (61 genes), sarcomere and hypertrophy (47 genes), hypertrophy transcription factors (186 genes), sarcoplasmic reticulum (3 genes), and genes up-regulated in HF disease models (221 genes) (*Figure 7F*). More detailed alluvial diagrams that break down constituent genes of the enrichment terms and their parent annotation categories are visualized in Supplementary material online, *Figure S14*. Unsupervised hierarchal clustering was performed on MLPKO and cRAD^Δ/Δ^-MLPKO, and from Holmes *et al*.^29^ MLP^+/+^ and MLPKO, using a subset of genes representing each category (*Figure 7G* and *H*). Four of the cRAD^Δ/Δ^-MLPKO clustered together and showed down-regulation of hallmark HF genes, correlating with the Holmes *et al*.^29^ profiles of healthy MLP^+/+^ vs. MLPKO (*Figure 7H*). The present study’s MLPKO mice displayed a correlated profile (see Supplementary material online, *Figure S15A–D*) corresponding with the Holmes *et al*.^29^ MLPKO profile (see Supplementary material online, *Figure S15E–F*). Taken together, the transcriptomic signature suggests that RAD reduction of existing DCM attenuates pathological signal transduction and cellular changes that underly eccentric hypertrophy and failing myocardium.

## 4. Discussion

The main finding of this study is that targeting LTCC modulation is an effective therapeutic to reverse heart failure. Echocardiography and CMR imaging show that RAD reduction after the onset of DCM restores cardiac function and structure. Histological analysis confirms wall thickening accompanied by increased cross-sectional area of cardiomyocytes. Mechanistically, RAD reduction increases trigger Ca^2+^ provided by the LTCC. In the absence of RAD, cardiomyocyte I_Ca,L_ is modulated without a requirement for β-AR/cAMP stimulation.^10^ We coin the term ‘deRADulated’ I_Ca,L_ to describe the direct LTCC modulation that is independent of β-AR-cAMP-signalling axis. In turn, deRADulated I_Ca,L_ promotes enhanced myocardial Ca^2+^-cycling. In DCM cardiomyocytes, Ca^2+^ transients exhibit reduced amplitudes and slower decay rates. In contrast deRADulated Ca^2+^ transients show increased amplitudes and faster cytosolic Ca^2+^ reuptake rates. This translates to sarcomere dynamics whereby shortening is increased, and relaxation is faster. Taken together, RAD-reduction acutely addresses a fundamental defect in HFrEF—reduced contractility.

In the MLPKO mouse, Ca^2+^-handling is central to pathogenesis^18,54^ and targeting Ca^2+^ re-uptake is an effective strategy to blunt the appearance of DCM in MLPKO mice. In this vein, induction of RAD knockout after onset of DCM rescued heart structure and function comparably to pre-protection displayed in MLPKO-PLNKO^55^ mice and transgenic mice over-expressing the micropeptide DWORF.^56^ De-RADulation rescue of DCM was accompanied by I_Ca,L_ with significant increases in the fast component of current decay as in healthy cRAD^Δ/Δ^ mice.^10^ The fast phase of I_Ca,L_ decay is known to be a calcium-dependent inactivation regulated by calmodulin and calcium/calmodulin kinase II (CaMKII). A CaMKII contribution to regulating Ca^2+^ reuptake and relaxation also appears as FDAR.^33,35^ Thus, rescue of lusitropy by RAD deletion is likely the result of increased activated dyadic CaMKII and by faster I_Ca,L_ decay due to increased calcium-dependent inactivation. Troponin I and its phosphorylation status also impact sarcomere relaxation velocity, and its gene expression is down-regulated in MLPKO mice.

RAD knockdown offers a novel approach to target cardiomyocyte L-type calcium channels in a highly specific manner while bypassing cAMP. Interventional cRAD^Δ/Δ^ achieved a similar amelioration of pathological remodelling as observed in protective studies of the MLPKO mouse targeting angiotensin II (AT1aKO MLPKO)^28^ and β-AR signalling (βARKct MLPKO).^18^ These models showed reduced excitation-transcription signalling that targets pro-hypertrophic and pathological remodelling. In this vein, downstream excitation-transcription effectors’ (e.g. GATA4 and CREB) target genes’ expression were attenuated in cRAD^Δ/Δ^, notably TNNT2, NPPA, NPPB, ACTA1, LRRC10, and MYH7 are among transcripts down-regulated by RAD knockout intervention of DCM. DeRADulated LTCC intervention of DCM and DWORF pre-protection^23,56^ show similar transcriptional changes. This suggests that signalling intermediates converge downstream of the LTCC and Ca^2+^-homeostasis. Taken together, our data are consistent with the formulation that the tension integral is a key final common endpoint from which cardiomyocytes sense and adapt structure-function.^57^

In summary, this study introduces a new potential therapeutic approach for treatment of systolic heart failure that acts via direct LTCC modulation independent of cAMP. A priori, we postulated that deRADulated LTCC would provide enhanced trigger Ca^2+^, with larger peak but faster decaying Ca^2+^ transients, to restore systolic function in the setting of existing DCM. We show as proof of principle, the ability to rescue cardiac dysfunction after adult-onset DCM in the MLPKO mouse, and in parallel demonstrate the positive inotropic benefit of RAD reduction in human non-ischaemic failing heart. Gene transcription changes, including reversal of foetal gene programme, highlight the complex nature of deRADulated LTCC. Future studies will dissect relevant signalling pathways recently associated with RAD in the heart.^58^ Lastly, safety concerns, including potential arrhythmogenesis were not noted in mice but require additional studies.

Translational Perspective Heart failure with reduced ejection fraction (HFrEF) is prevalent and treatment options are not optimal. This study provides evidence that targeting L-type calcium channel (LTCC) regulation provides restoration of contractile function and reverse remodelling of dilated cardiomyopathy. Reduction of the protein RRAD promoted increased contractility in the absence of sympathetic adrenergic system activation. Studies were conducted on human HFrEF myocardial ventricular strips and in a mouse model of dilated cardiomyopathy. The key new insight from this work is to introduce LTCC modulation as a nexus for reverse remodelling HFrEF.

## Supplementary Material

cvaf169_Supplementary_Data

## Data Availability

The bulk RNA-seq data have been uploaded and will be available on the GEO database (GSE304830). The data, methods used in the analysis, and materials will be made available to any researcher for purposes of reproducing the results or replicating the procedure on reasonable request to the corresponding author.
