# Supplementary material for: RRAD-reduction reveals efficacy of targeting L-type calcium channel regulation for treatment of heart failure
Source: Cardiovasc Res. 2025 Oct 1;121(14):2204–21. doi: 10.1093/cvr/cvaf169 (PMC12638741; doi:10.1093/cvr/cvaf169)
Supplement: cvaf169_Supplementary_Data [file cvaf169_supplementary_data.zip › CVR_2025_03_26_Supp Fig Legends V5.docx]

**Supplemental Figure Legends**

**Supplemental Figure 1.** Cardiomyocyte-specific RAD deletion rescues cardiac dysfunction of the muscle lim protein knockout mouse model of dilated cardiomyopathy. **(A)** Representative M-mode echocardiographic tracings from female mice. **(B)** Individual mice plotted for left ventricular fractional shortening shown in summary plots in Fig. 1 before and 1- and 2-months after cRAD^Δ/Δ^ induction. **(C)** Individual mice plotted for left ventricular internal diameter; diastolic shown in summary plots in Fig. 1 before and 1- and 2-months after cRAD^Δ/Δ^ induction. **(D)** Mean and individual mouse HR ratio (left ventricular wall to chamber ratio). cRAD^Δ/Δ^ had a significant treatment effect (genotype*timepoint, p= 1E-4, F= 9.59) on wall to chamber ratio (linear mixed model accounting for genotype [p=6E-12, F=54.91], and timepoint [repeated measures] [p= 3E-3, F=6.04] as fixed factors. The sex fixed factor was not significant (p= 0.065, F= 3.44). The means of male pre-tamoxifen MLPKO RAD^fl/fl^ (0.18 [95% CI 0.16, 0.20], N= 27) and MLPKO RAD^fl/fl^ MYH6-MerCreMer (0.19 [0.17, 0.20], N= 21) were not significantly different. The means of male 1- and 2-months post-tamoxifen cRAD^Δ/Δ^-MLPKO (0.25 [0.23, 0.27], N= 37 and 0.31 [0.25, 0.37], N= 8) were significantly greater than MLPKO (0.17 [0.15, 0.18], N= 34 and 0.14 [0.12, 0.16], N= 8). The means of female pre-tamoxifen MLPKO RAD^fl/fl^ (0.19 [0.16, 0.22], N= 14) and MLPKO RAD^fl/fl^ MYH6-MerCreMer (0.22 [0.16, 0.27], N= 21) were not significantly different. The means of 1- and 2-months post-tamoxifen cRAD^Δ/Δ^-MLPKO (0.28 [0.25, 0.30], N= 37 and 0.28 [0.24, 0.32], N= 16) were significantly 38% and 50% greater than MLPKO (0.20 [0.18, 0.22], N= 31 and 0.19 [0.16, 0.21], N= 9). The wall to chamber ratio was calculated as the average of the left ventricular posterior and anterior walls divided by internal diameter (diastolic). A post-hoc Tukey test was used for pairwise comparisons reported at each timepoint. Significant differences are reported as percent difference of the treatment group increases or decreases relative to the control at the same time point.

**Supplemental Figure 2.** The effect size of cRAD^Δ/Δ^ rescue of cardiac dysfunction of the MLPKO mouse in comparison to published MLPKO studies. Aggregated means ±SEM of fractional shortening **(A)** and left ventricular internal diameter; diastolic **(B)** of M-mode echocardiography from published (labeled “literature”) studies of MLPKO, their same-strain wild type control MLP^+/+^, double transgenic protection (constitutive knockout/overexpression) mice, plotted with the present study control MLPKO and interventional cRAD^Δ/Δ^-MLPKO (1-month post-tamoxifen timepoint). The data represents pooled sex, as most published studies did not report separate male and female quantification. SEM for the published data was calculated from considering each study as a sample. Detailed statistics and information on each study is in Supp. Table 5.

**Supplemental Figure 3.** Mouse body weights for longitudinally tracked mice (**A**) and at endpoint (**B**). **(A)** Longitudinal male (left) and female (right) mean and individual body weight (below). There was not a significant main effect of genotype, but there were significant sex (p= 2E-51) and timepoint effects (p= 4E-6, F= 13.161) and those terms’ interaction (p= 0.002, F= 6.283). The estimated marginal means for male weight at 2.5 months (29.0±0.34 g), 3.5 months (31.3±0.31 g), and 4.5 months (33.4±0.42 g) months were significantly higher than female weight at 2.5 months (22.4±0.37 g), 3.5 months (23.9±0.31 g), and 4.5 months (24.1±0.39 g). A post-hoc Tukey test was used for pairwise comparisons reported at each timepoint. For the timepoints (2.5, 3.5, 4.5 months), N= 34, 53, 17 for MLPKO; 33, 62, 20 for cRAD^Δ/Δ^-MLPKO. **(B)** Mouse weights of male (left) and female (right) at takedown (~2 months post treatment, mean age= 19.1 weeks). Mouse weight did not differ by genotype (p= 0.091, F= 2.900) but did differ by sex (p= 2E-36, F= 355.317) (two-way ANOVA, interaction p= 0.144, F= 2.167). The marginal mean of male weight (32.2±0.33 g) was significantly higher than female (23.1±0.35 g).

**Supplemental Figure 4.** **(A)** Female heart weights normalized to body weights (mg/g) at 1- (Left) and 2-months post tamoxifen (Right), cRAD^Δ/Δ^-MLPKO (mean 7.19 [95% CI 5.80, 8.58], N= 10, and median 8.59 [Q1,Q3 7.39, 9.62], N= 8) and MLPKO (8.42 [7.80, 9.05], N= 13, and 6.45 [6.41, 7.30], N= 11) were not significantly different (independent samples t-test, t= 1.96, p= 0.063; Mann-Whitney U= 31.0, p= 0.310 ). **(B)** Female wet lung weights normalized to body weights (mg/g). Respectively at 1- (Left) and 2-months post tamoxifen (Right), cRAD^Δ/Δ^-MLPKO (median 7.12 [ Q1, Q3 6.29, 7.94], N= 10, and mean 6.85 [6.08, 7.61], N= 10) and MLPKO (7.97 [7.26, 8.68], N= 15, and 7.02 [5.53, 8.50], N= 7) were not significantly different (Mann-Whitney U test, U= 107.0, p= 0.080; independent samples t-test, t= 0.27, p= 0.795). Mann-Whitney U tests were used for data which Shapiro-Wilk normality tests suggested a deviation from normality, and medians and the lower (Q1) and upper (Q3) were reported accordingly.

**Supplemental Figure 5.** **(A)** Representative short axis micrographs of wheat germ agglutinin (WGA) stained 2-months-post-treatment MLPKO and cRAD^Δ/Δ^-MLPKO (4.5 months old) hearts. (Right) 40X micrographs of left ventricular wall showing cardiomyocytes in cross-section. Scale bars are 2 mm (whole heart) and 50 µm (40X images). **(B)** Mean cardiomyocyte cross-sectional area was significantly increased in cRAD^Δ/Δ^-MLPKO mice (linear mixed model, cells nested into mice, genotype F= 12.733, p= 0.001, sex F= 15.992, p= 5E-4, genotype*sex F= 3.953, p= 0.058). Mean female area (148.4 µm^2^ [132.4, 164.4]) was not significantly different from MLPKO (136.4 µm^2^ [123.4, 149.5]) (post-hoc Holm p= 0.258). **(C)** The probability density function (PDF) reveals a larger proportion of cRAD^Δ/Δ^-MLPKO cardiomyocytes had increased cross-sectional area. **(D)** The proportion of female cells with area ≥200 µm^2^ was significantly higher in cRAD^Δ/Δ^-MLPKO (21% versus 18%) than MLPKO. (Fisher’s exact test, p<0.0001). N= 29 total mice (15 female), 30 technical replicate images per heart from 2 sections, and 110,060 total cells (57,177 female).\

**Supplemental Figure 6.** cRAD^Δ/Δ^-MLPKO does not affect fibrosis. (A) Picrosirius Red stained short axis murine heart sections. (B) The degree of fibrosis was not significantly different considering genotype or sex (two-way ANOVA, genotype F= 0.084, p= 0.774; sex F= 1.612, p= 0.2175; interaction F= 0.3910, p= 0.5382). Four blinded researchers scored 2 technical replicate sections per mouse heart (5= extremely fibrotic). N= 6 male, 9 female MLPKO; 8 male, 3 female cRAD^Δ/Δ^-MLPKO.

**Supplemental Figure 7.** Preservation of cRAD^Δ/Δ^ rescue of cardiac dysfunction in MLPKO in long-term study of 1-year old mice (45 weeks post cRAD^Δ/Δ^ induction). **(A)** Mean left ventricular fractional shortening of cRAD^Δ/Δ^-MLPKO (50.7±3.3 % SEM) was significantly 70% higher than that of MLPKO (29.8±2.4 %) (two-way ANOVA: genotype p= 5.4E-5, F= 22.595; sex p= .040, F= 4.632; interaction p= .084, F= 3.218). **(B)** Mean left ventricular internal diameter; diastolic (mm) of cRAD^Δ/Δ^-MLPKO (3.37±0.20 mm) was significantly reduced 20% relative to MLPKO (4.21±0.21 mm) (two-way ANOVA: genotype p= .005, F= 9.413; sex p= .006, F= 8.756; interaction p= .355, F= .885). **(C)** Mean HR ratio (mean wall thickness to LV inner chamber diameter ratio, at diastole) of cRAD^Δ/Δ^-MLPKO (0.371±.039) was significantly 51% higher than that of MLPKO (.245±.019) (two-way ANOVA: genotype p= .030, F= 5.218; sex p= .218, F= 1.586; interaction p= .080, F= 3.306). **(D)** Weight of the mice did not differ significantly by genotype but did by sex (two-way ANOVA: genotype p= .058, F= 3.906; sex p= 2E-10, F= 90.257; interaction p= .512, F= .441); males were 46% heavier (40.5±1.8 g) versus female 27.7±0.5 g.

**Supplemental Figure 8.** Representative family of traces of L-type calcium channel current density of cRAD^Δ/Δ^-MLPKO and MLPKO 1-2 months after tamoxifen (3.5-4.5 mo. old). V_hold_ = 80mV, V_test_, 300ms duration in increments of +5 mV from -75mV to +40 mV at 5 s intervals.

**Supplemental Figure 9**. Individual cell data for kinetic analysis of I_Ca,L_. (A) Percent remaining current 30 ms after peak. cRAD^Δ/Δ^-MLPKO had significantly less remaining current (faster decay) (linear mixed model; genotype p= 4E-5, F= 35.01; voltage p= 0.012, F=2.75, genotype x voltage p= 0.542, F= 0.86). The remaining current was on average reduced 25% in cRAD^Δ/Δ^-MLPKO (49% remaining current) relative to MLPKO (67%). (B) Fractional remaining current 150 ms after peak across various test potentials. cRAD^Δ/Δ^-MLPKO had significantly less remaining current (faster decay) (linear mixed model; genotype p= 0.012, F= 8.20; voltage p= 4E-5, F= 5.23; interaction p= 0.812, F= 0.53). The remaining current was on average reduced 24% in cRAD^Δ/Δ^-MLPKO (19% remaining current) relative to MLPKO (26%). For MLPKO (N=5 mice, n= 8 cells); for cRAD^Δ/Δ^-MLPKO (N= 5 mice, n= 12 cells). (C) Post-hoc Holm-Sidak results for each V_test_.

**Supplemental Figure 10**. Individual paired cell data for live cell Ca^2+^ and sarcomere imaging at different pacing frequencies. Note each cell was paired and was exposed to the same regimen of pacing (1, 2, 3, 0.1 Hz). For clarity, connecting lines have been omitted in these plots. For cRAD^Δ/Δ^-MLPKO, N= 3 mice, 39 cells, and 156 observations; MLPKO, N= 5 mice, 55 cells, and 220 observations.

**Supplemental Figure 11**. Sarcoplasmic reticulum Ca^2+^ load is increased in cRAD^Δ/Δ^-MLPKO. (A) Summary data of the fura-2-AM ratio of calcium transients of twitch (1 Hz pacing in vehicle solution) (black) and after caffeine (purple). There were significant main effects of genotype (F= 7.441, p= 0.0149) and caffeine (F= 57.65, p< 0.0001) in addition to a significant interaction (F= 9.223, p= 0.0078). The caffeine mean amplitude was significantly higher in cRAD^Δ/Δ^-MLPKO (1.44 [95% CI, 1.01, 1.87]) versus MLPKO (0.83 [0.58, 1.08]) (post-hoc Holm-Sidak, p= 0.0459).

**Supplemental Figure 12**. shRNA.RRAD reduces RRAD RNA in human strips. RNA transcript for RRAD was reduced > 5-fold by sh.RNA treatment compared to control. (t-test, t=23.33; P<10^-4^). N=9 control and N=9 sh.RNA.RRAD

**Supplemental Figure 13.** Shared downregulated genes of MLP^+/+^ and cRAD^Δ/Δ^-MLPKO, versus MLPKO ranked by magnitude. Individual genes that appeared in the correlation plot (**Figure 8B**) are ranked by magnitude of log_2_ fold-change.

**Supplemental Figure 14.** Alluvial diagrams of shared downregulated genes, their enriched gene set terms, and curated annotations. This graph supplements the alluvial diagram in **Figure 8F,** showing annotation of specific genes (e.g. what enriched gene sets the gene *Nppa* is in or what enriched gene sets make up the “Sarcomere & Hypertrophy” term).

**Supplemental Figure 15.** Clustered heatmaps of transcriptomic profiles. **(A)** The top 500 genes ranked by variance were used in an unsupervised hierarchical clustering algorithm that clustered each sample (N= 5 MLPKO, 5 cRAD^Δ/Δ^-MLPKO) and genes into modules. Each gene module was annotated using the (B) pathway, (C) transcription factors, and (D) GO databases. This analysis was also performed on published MLP^+/+^ versus MLPKO at 3, 6 and 10 weeks old (N= 3 mice per group). In the heatmaps, blue= downregulated and red= upregulated. Expressions are z-scores.
