# Supplementary material for: RRAD-reduction reveals efficacy of targeting L-type calcium channel regulation for treatment of heart failure
Source: Cardiovasc Res. 2025 Oct 1;121(14):2204–21. doi: 10.1093/cvr/cvaf169 (PMC12638741; doi:10.1093/cvr/cvaf169)
Supplement: cvaf169_Supplementary_Data [file cvaf169_supplementary_data.zip › Suppl Fig_2025_04_16_2025 V5 R1.pptx]

## Slide 1
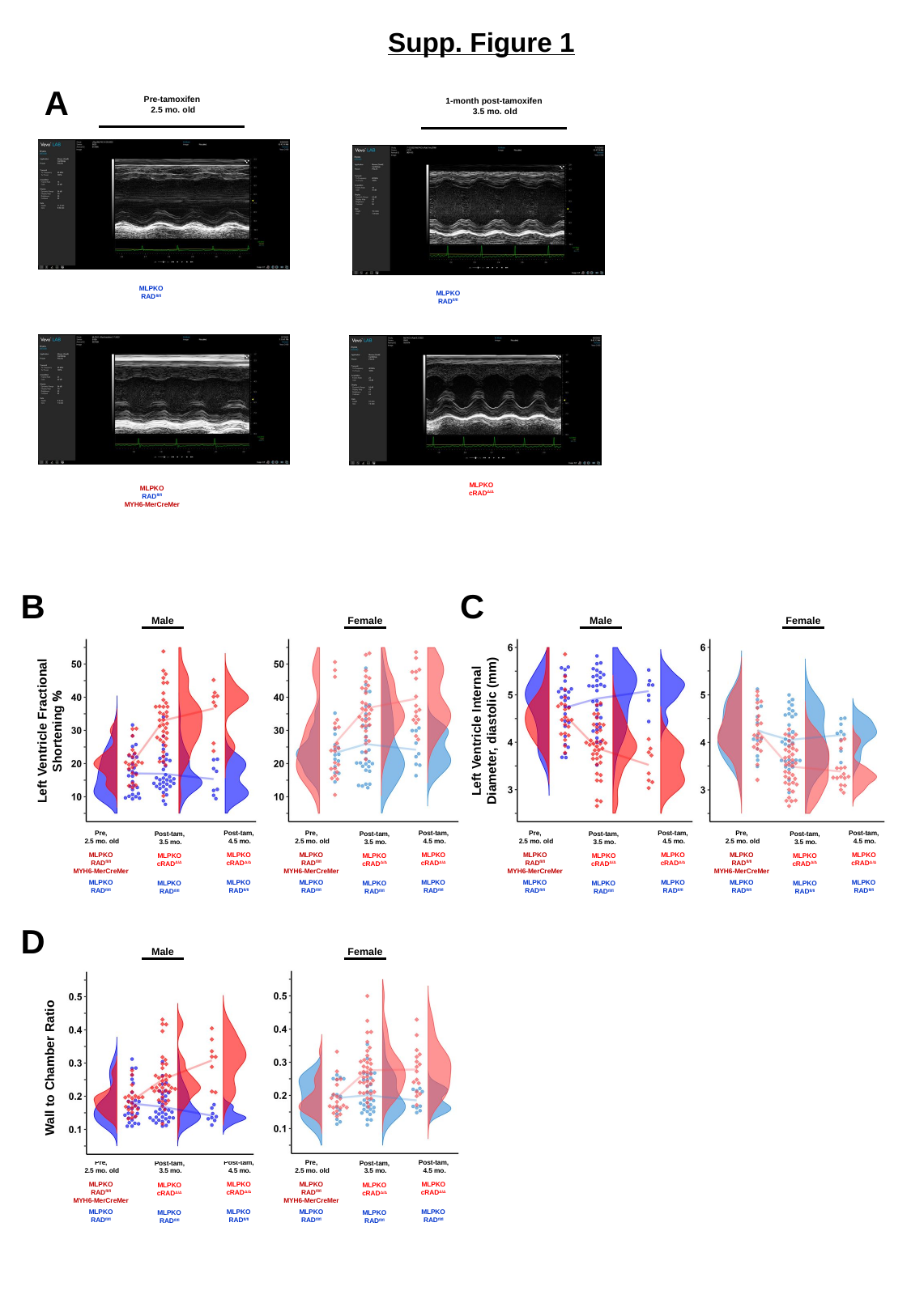

Supp. Figure 1
A
Pre-tamoxifen
 2.5 mo. old
1-month post-tamoxifen
 3.5 mo. old
MLPKO
RADfl/fl
MLPKO
RADfl/fl
MLPKO
cRADΔ/Δ
MLPKO
RADfl/fl
MYH6-MerCreMer
B
C
Male
Female
Male
Female
Left Ventricle Fractional Shortening %
Left Ventricle Internal Diameter, diastolic (mm)
Pre,
 2.5 mo. old
Post-tam,
 4.5 mo.
Post-tam,
 3.5 mo.
MLPKO
RADfl/fl
MYH6-MerCreMer
MLPKO
cRADΔ/Δ
MLPKO
cRADΔ/Δ
MLPKO
RADfl/fl
MLPKO
RADfl/fl
MLPKO
RADfl/fl
Pre,
 2.5 mo. old
Post-tam,
 4.5 mo.
Post-tam,
 3.5 mo.
MLPKO
RADfl/fl
MYH6-MerCreMer
MLPKO
cRADΔ/Δ
MLPKO
cRADΔ/Δ
MLPKO
RADfl/fl
MLPKO
RADfl/fl
MLPKO
RADfl/fl
Pre,
 2.5 mo. old
Post-tam,
 4.5 mo.
Post-tam,
 3.5 mo.
MLPKO
RADfl/fl
MYH6-MerCreMer
MLPKO
cRADΔ/Δ
MLPKO
cRADΔ/Δ
MLPKO
RADfl/fl
MLPKO
RADfl/fl
MLPKO
RADfl/fl
Pre,
 2.5 mo. old
Post-tam,
 4.5 mo.
Post-tam,
 3.5 mo.
MLPKO
RADfl/fl
MYH6-MerCreMer
MLPKO
cRADΔ/Δ
MLPKO
cRADΔ/Δ
MLPKO
RADfl/fl
MLPKO
RADfl/fl
MLPKO
RADfl/fl
D
Male
Female
Wall to Chamber Ratio
Pre,
 2.5 mo. old
Post-tam,
 4.5 mo.
Post-tam,
 3.5 mo.
MLPKO
RADfl/fl
MYH6-MerCreMer
MLPKO
cRADΔ/Δ
MLPKO
cRADΔ/Δ
MLPKO
RADfl/fl
MLPKO
RADfl/fl
MLPKO
RADfl/fl
Pre,
 2.5 mo. old
Post-tam,
 4.5 mo.
Post-tam,
 3.5 mo.
MLPKO
RADfl/fl
MYH6-MerCreMer
MLPKO
cRADΔ/Δ
MLPKO
cRADΔ/Δ
MLPKO
RADfl/fl
MLPKO
RADfl/fl
MLPKO
RADfl/fl

## Slide 2
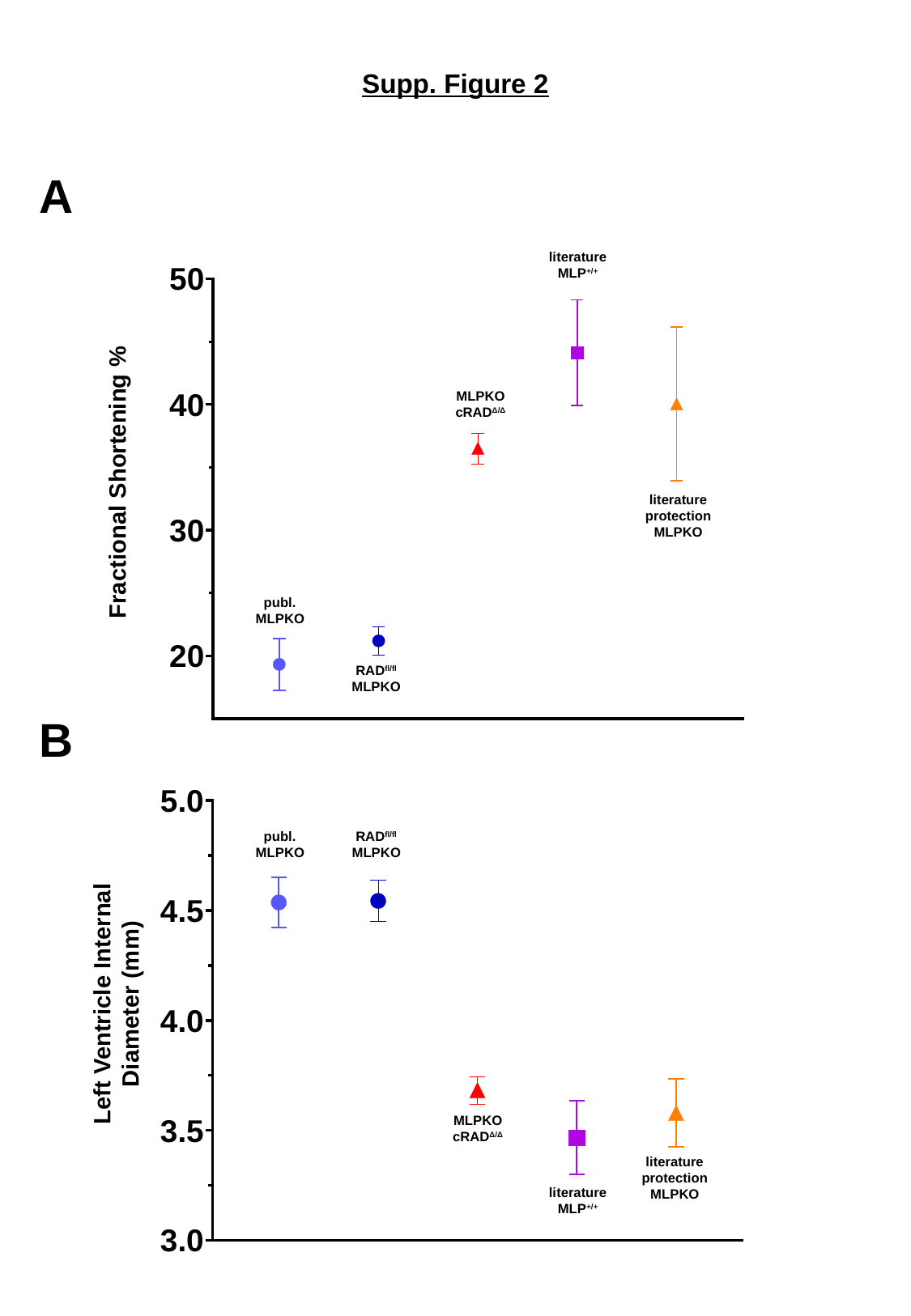

Supp. Figure 2
A
literature
MLP+/+
MLPKO
cRADΔ/Δ
Fractional Shortening %
literature
protection
MLPKO
publ.
MLPKO
RADfl/fl
MLPKO
B
publ.
MLPKO
RADfl/fl
MLPKO
Left Ventricle Internal Diameter (mm)
MLPKO
cRADΔ/Δ
literature
protection
MLPKO
literature
MLP+/+

## Slide 3
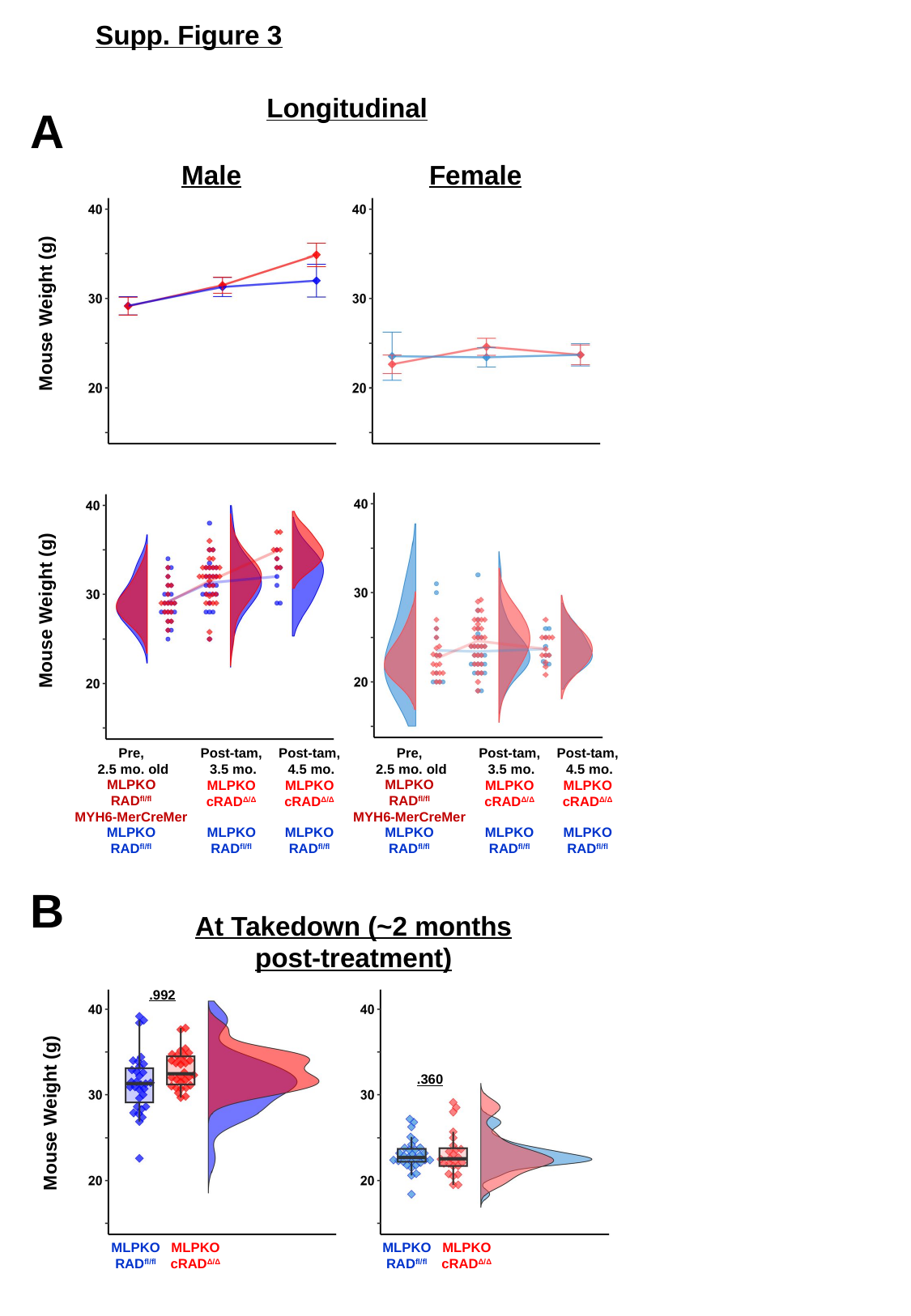

Supp. Figure 3
Longitudinal
A
Male
Female
Mouse Weight (g)
Mouse Weight (g)
Pre,
 2.5 mo. old
Post-tam,
 3.5 mo.
Post-tam,
 4.5 mo.
MLPKO
RADfl/fl
MYH6-MerCreMer
MLPKO
cRADΔ/Δ
MLPKO
cRADΔ/Δ
MLPKO
RADfl/fl
MLPKO
RADfl/fl
MLPKO
RADfl/fl
Pre,
 2.5 mo. old
Post-tam,
 3.5 mo.
Post-tam,
 4.5 mo.
MLPKO
RADfl/fl
MYH6-MerCreMer
MLPKO
cRADΔ/Δ
MLPKO
cRADΔ/Δ
MLPKO
RADfl/fl
MLPKO
RADfl/fl
MLPKO
RADfl/fl
B
At Takedown (~2 months post-treatment)
.992
.360
Mouse Weight (g)
MLPKO
RADfl/fl
MLPKO
cRADΔ/Δ
MLPKO
RADfl/fl
MLPKO
cRADΔ/Δ

## Slide 4
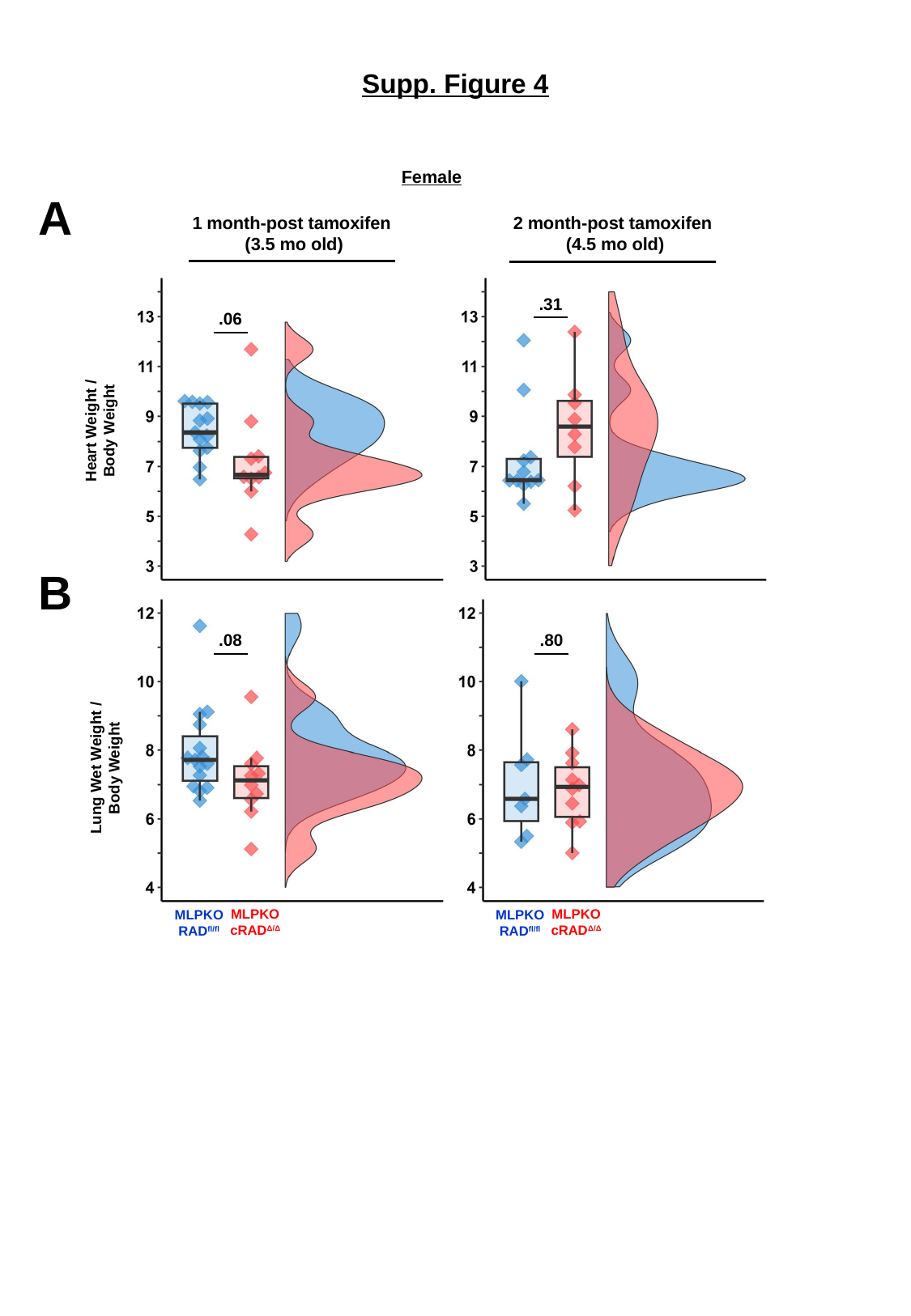

Supp. Figure 4
Female
A
1 month-post tamoxifen
 (3.5 mo old)
2 month-post tamoxifen
 (4.5 mo old)
.06
.31
Heart Weight / Body Weight
B
.08
MLPKO
cRADΔ/Δ
MLPKO
RADfl/fl
.80
MLPKO
cRADΔ/Δ
MLPKO
RADfl/fl
Lung Wet Weight / Body Weight

## Slide 5
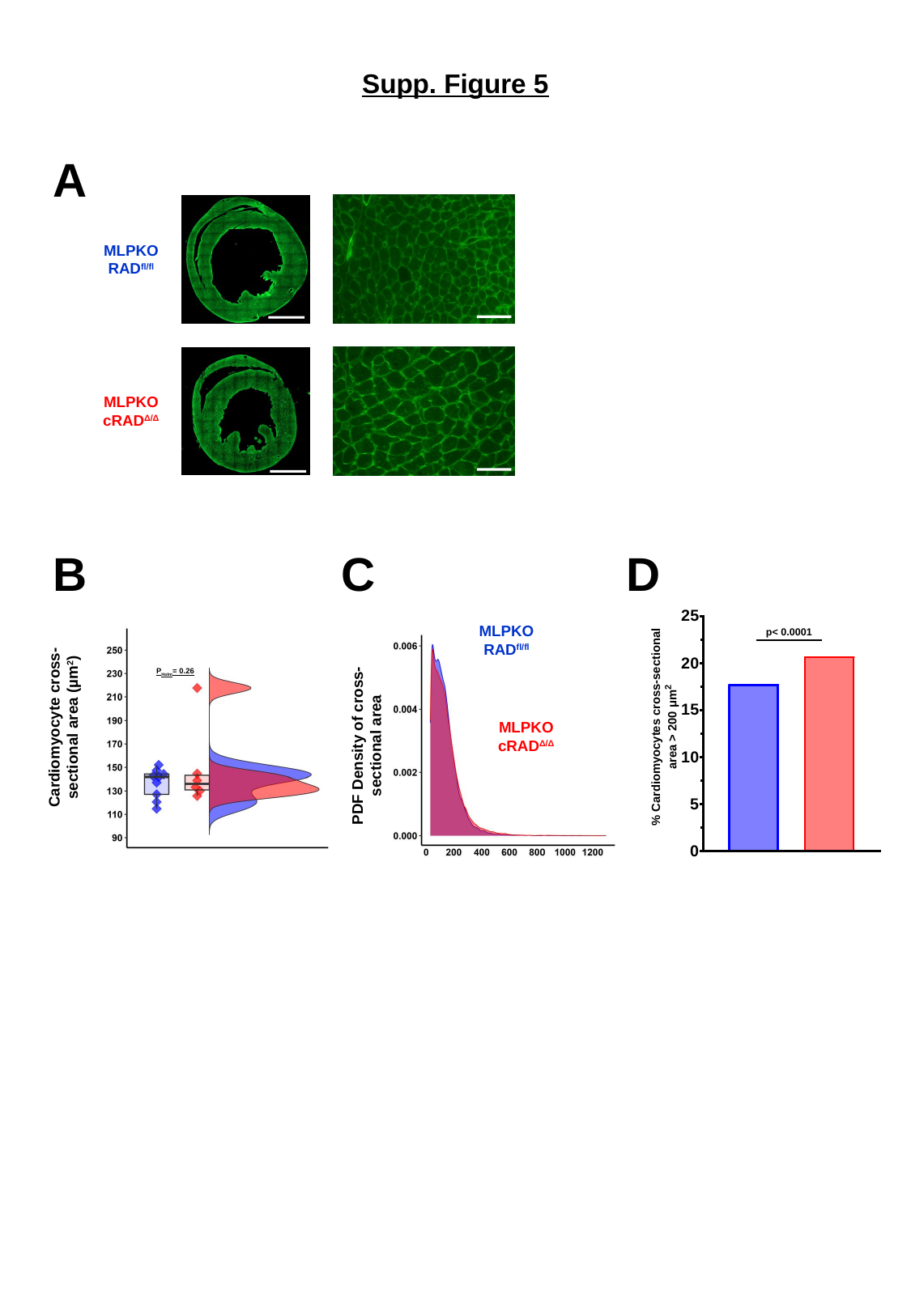

Supp. Figure 5
A
MLPKO
RADfl/fl
MLPKO
cRADΔ/Δ
B
C
D
MLPKO
RADfl/fl
MLPKO
cRADΔ/Δ
PDF Density of cross-sectional area
Cardiomyocyte cross-sectional area (µm2)
PHolm= 0.26

## Slide 6
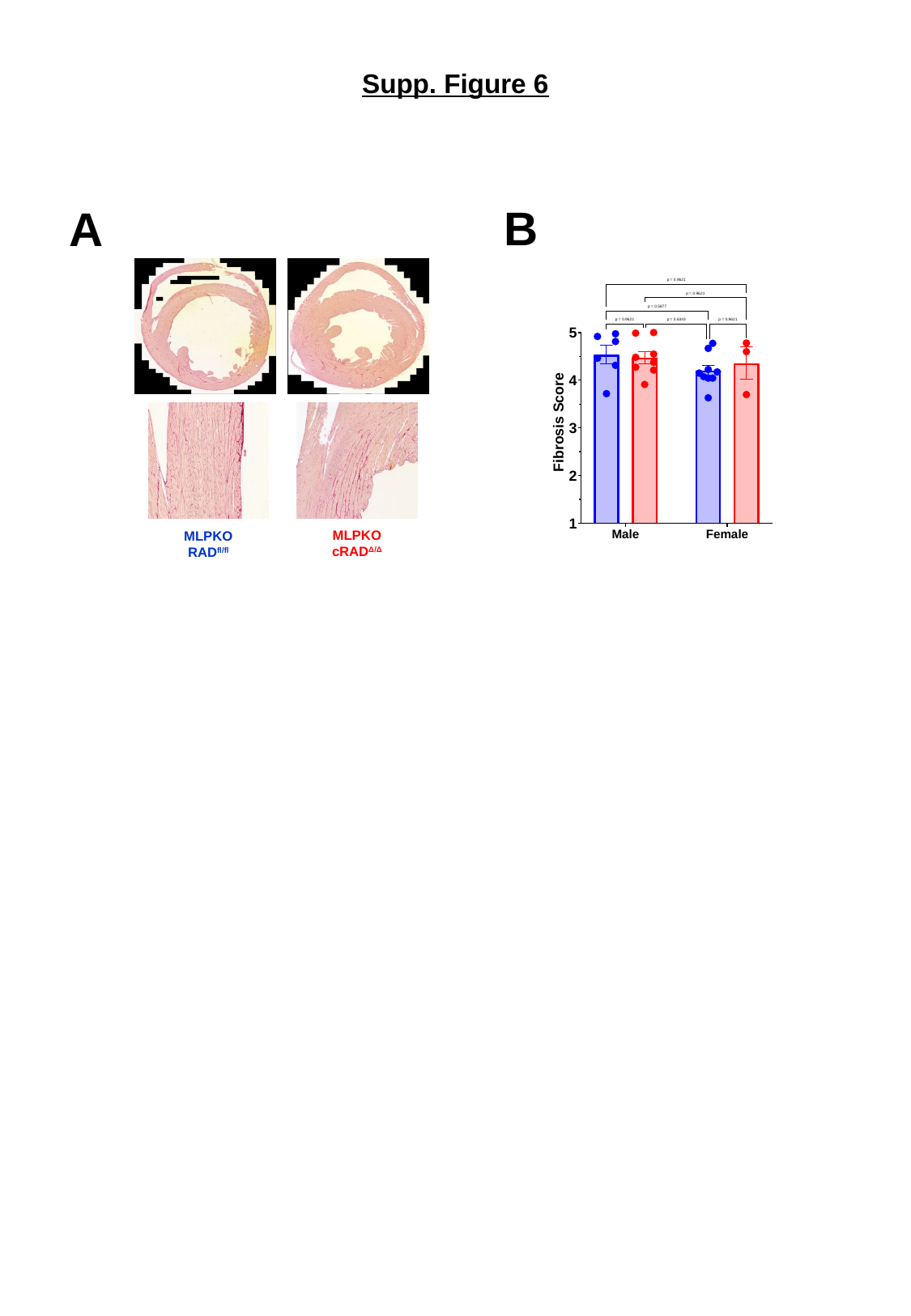

Supp. Figure 6
B
A
MLPKO
cRADΔ/Δ
MLPKO
RADfl/fl

## Slide 7
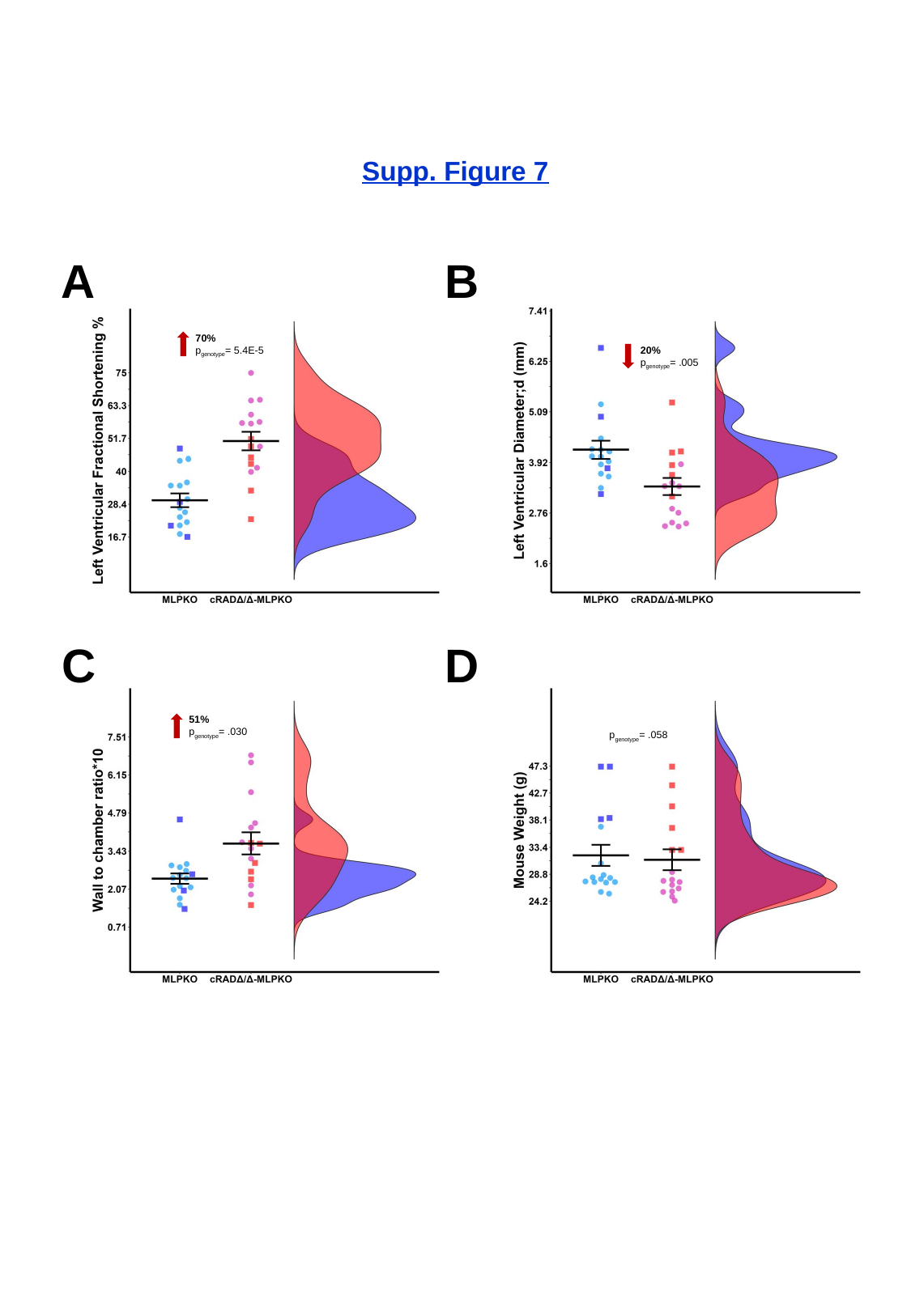

Supp. Figure 7
A
B
20%
pgenotype= .005
70%
pgenotype= 5.4E-5
C
D
 pgenotype= .058
51%
pgenotype= .030

## Slide 8
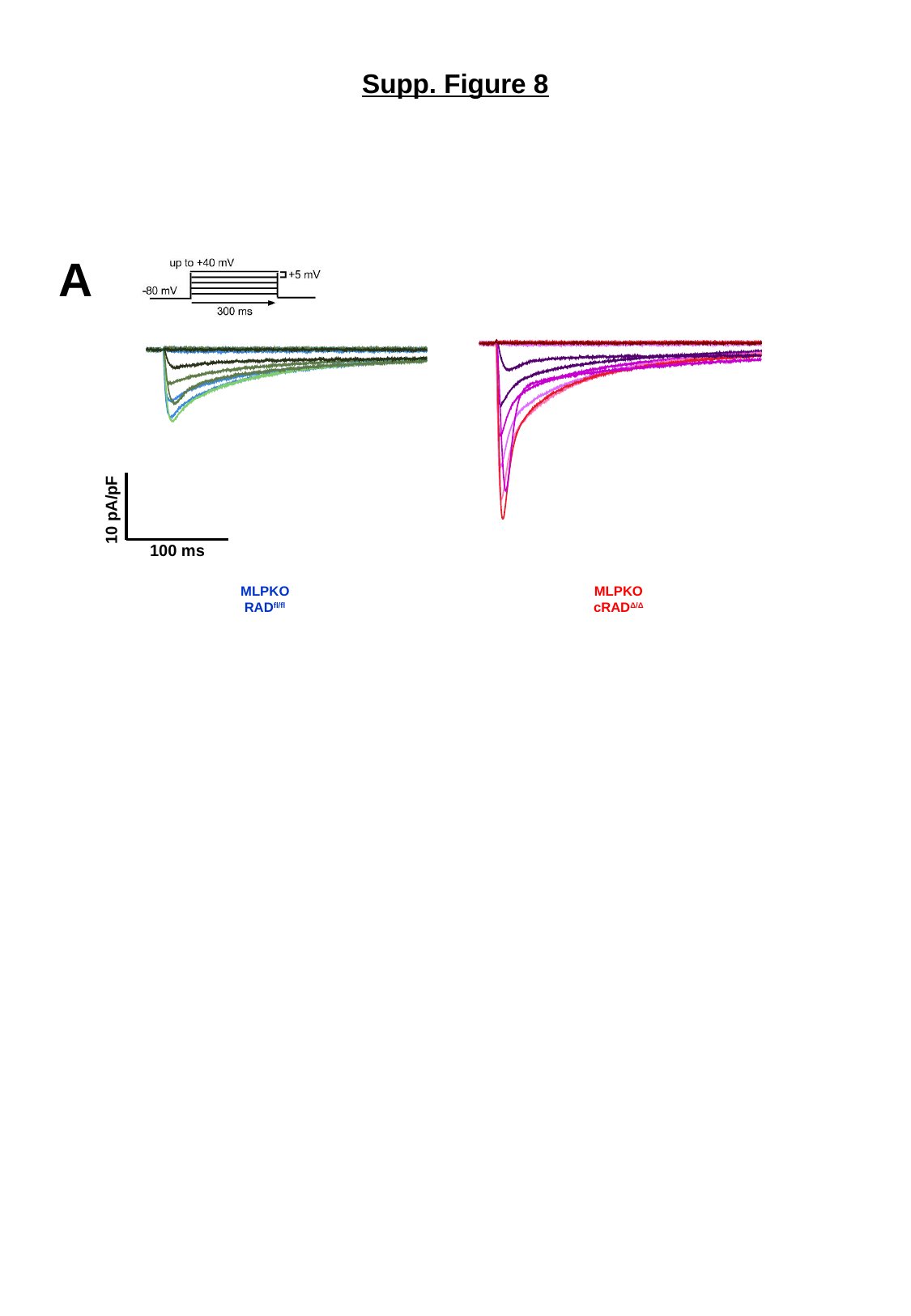

Supp. Figure 8
A
MLPKO
RADfl/fl
MLPKO
cRADΔ/Δ

## Slide 9
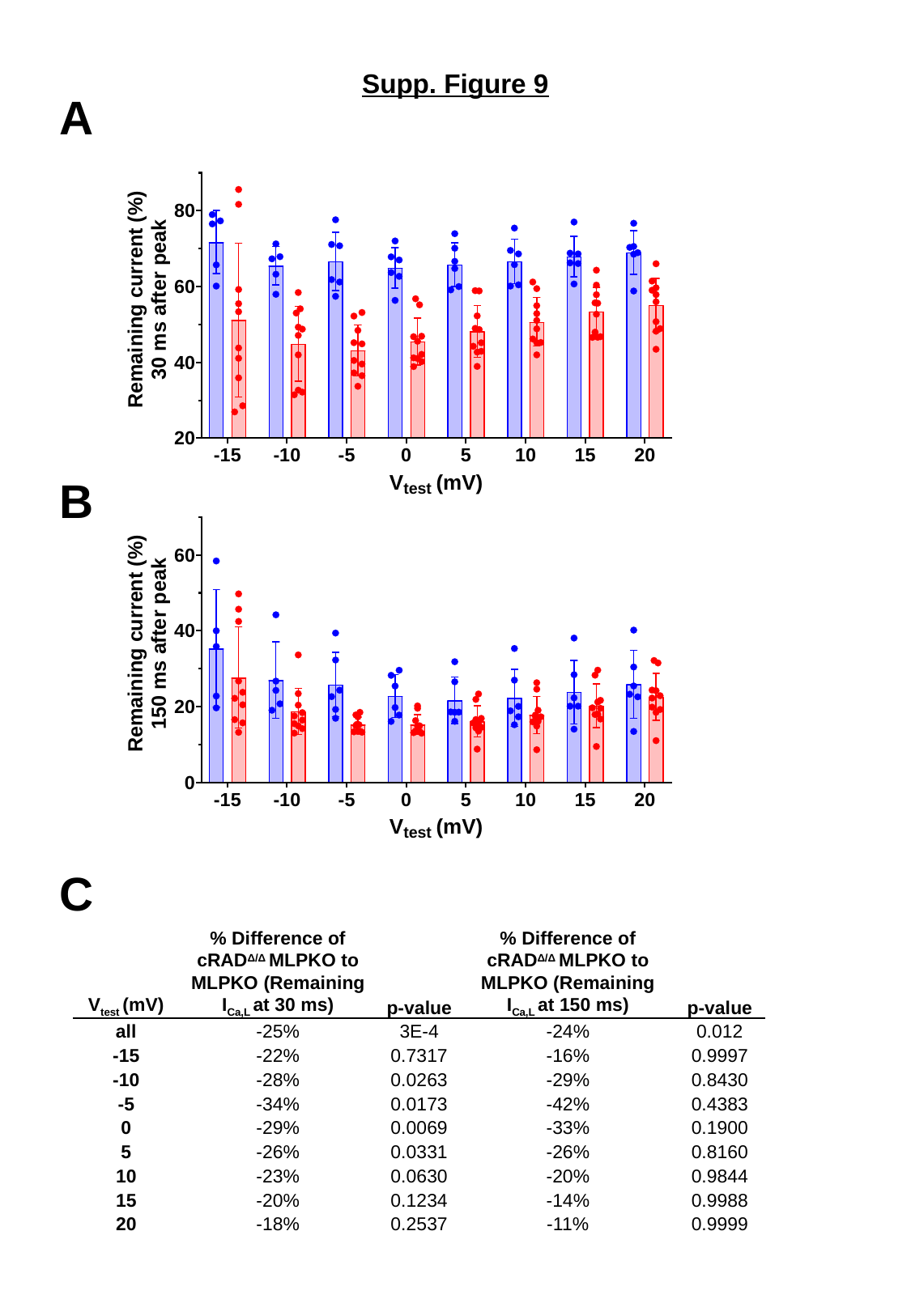

Supp. Figure 9
A
B
C
| Vtest (mV) | % Difference of cRADΔ/Δ MLPKO to MLPKO (Remaining ICa,L at 30 ms) | p-value | % Difference of cRADΔ/Δ MLPKO to MLPKO (Remaining ICa,L at 150 ms) | p-value |
| --- | --- | --- | --- | --- |
| all | -25% | 3E-4 | -24% | 0.012 |
| -15 | -22% | 0.7317 | -16% | 0.9997 |
| -10 | -28% | 0.0263 | -29% | 0.8430 |
| -5 | -34% | 0.0173 | -42% | 0.4383 |
| 0 | -29% | 0.0069 | -33% | 0.1900 |
| 5 | -26% | 0.0331 | -26% | 0.8160 |
| 10 | -23% | 0.0630 | -20% | 0.9844 |
| 15 | -20% | 0.1234 | -14% | 0.9988 |
| 20 | -18% | 0.2537 | -11% | 0.9999 |

## Slide 10
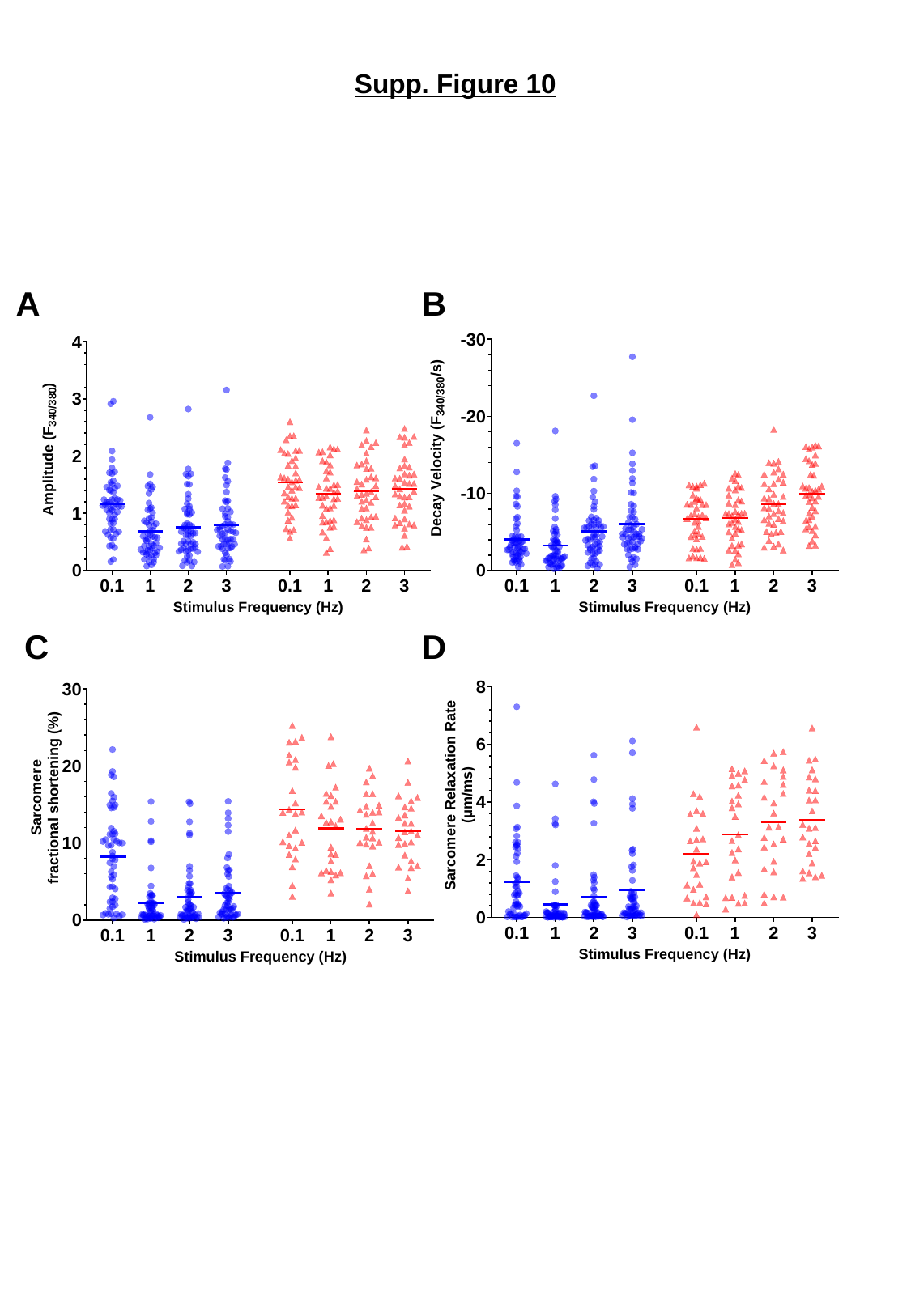

Supp. Figure 10
A
B
C
D

## Slide 11
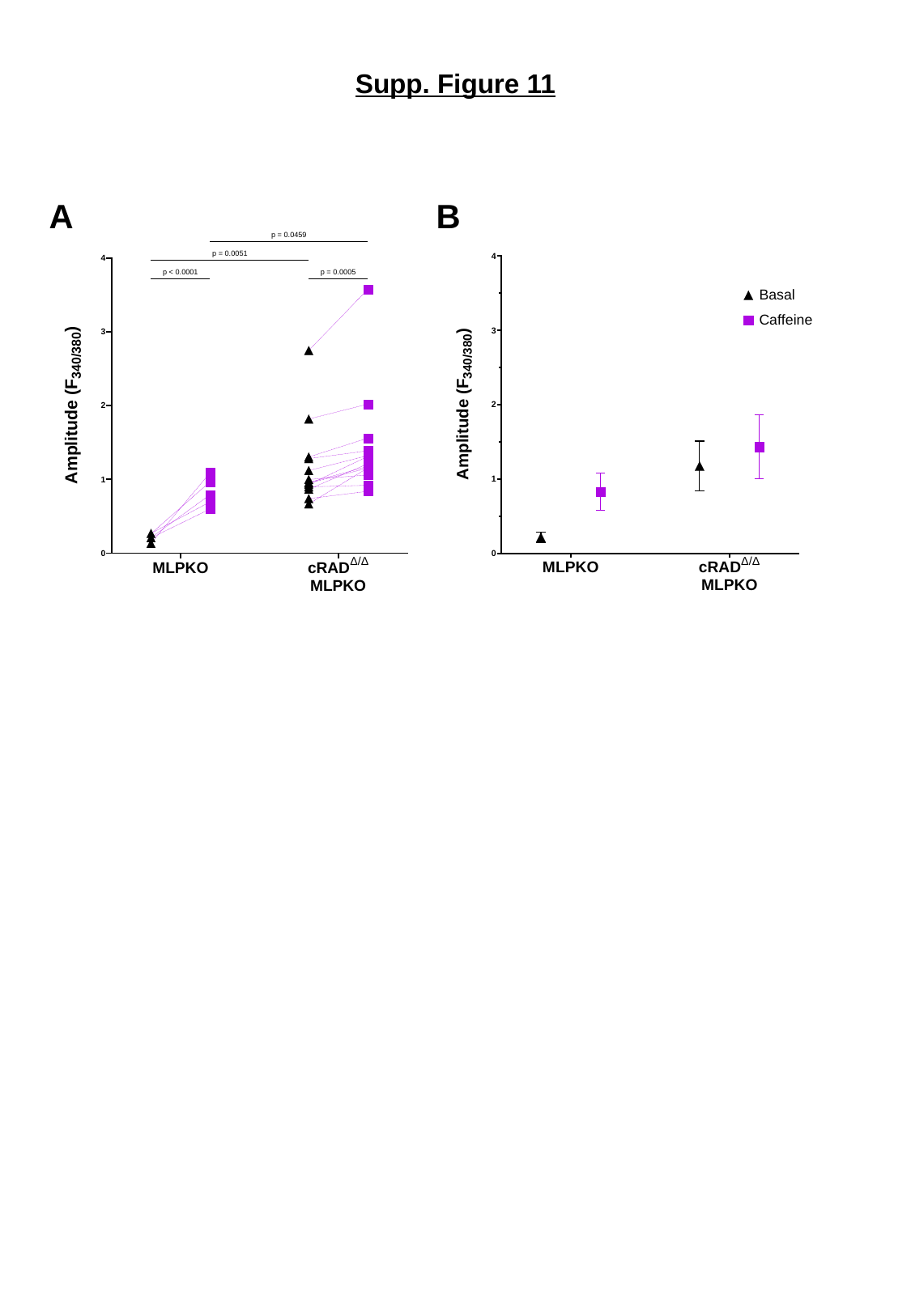

Supp. Figure 11
A
B

## Slide 12
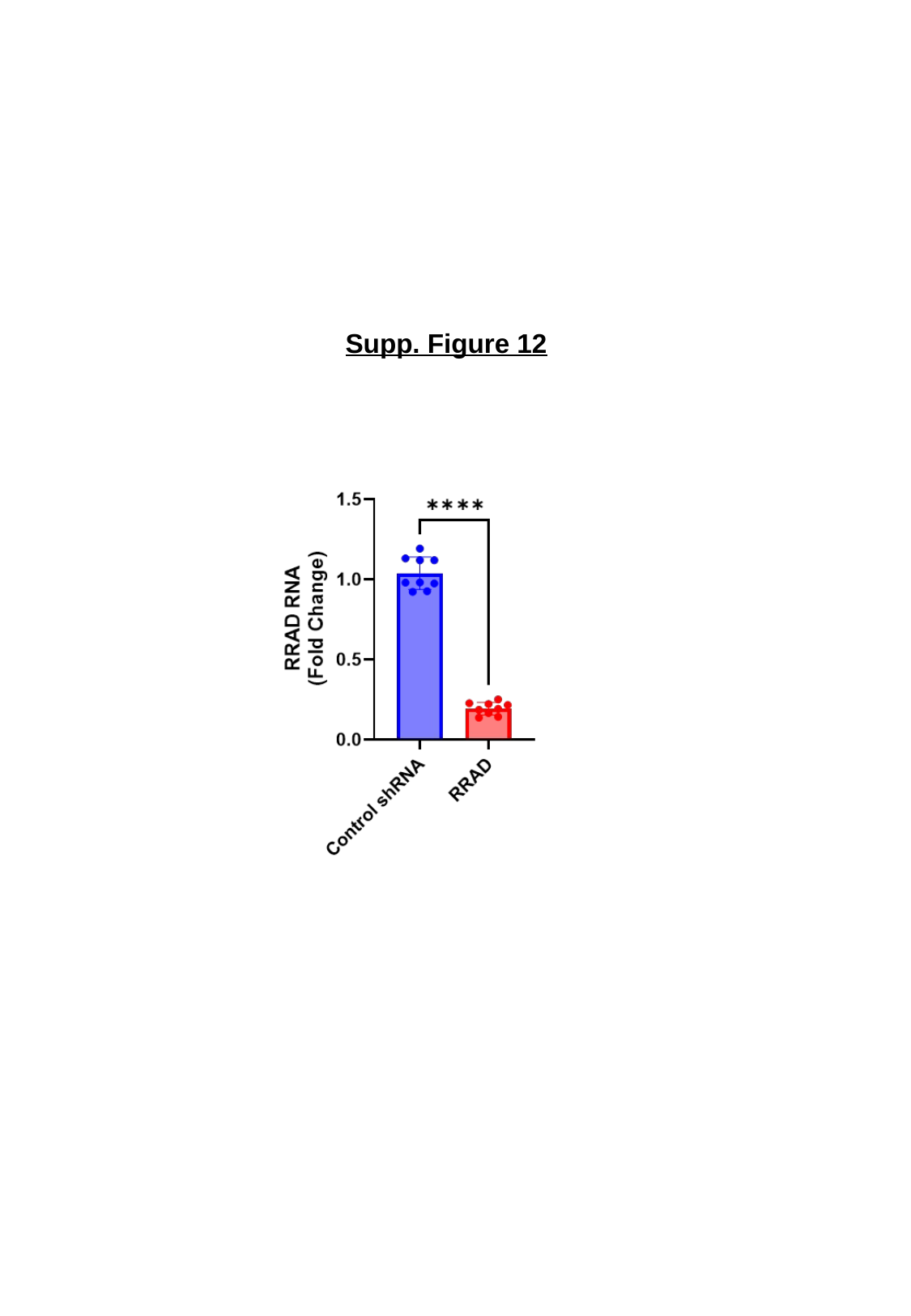

Supp. Figure 12

## Slide 13
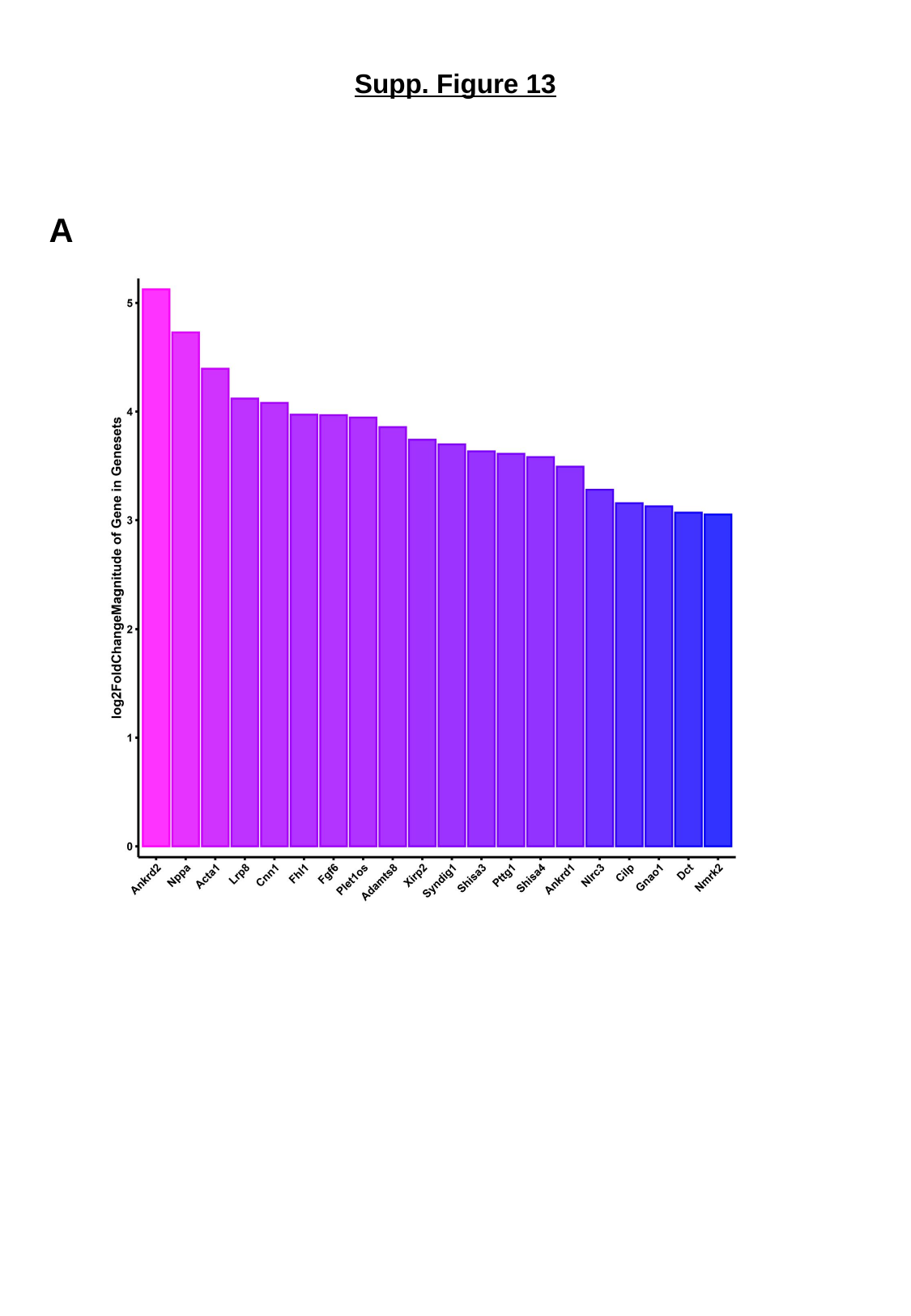

Supp. Figure 13
A

## Slide 14
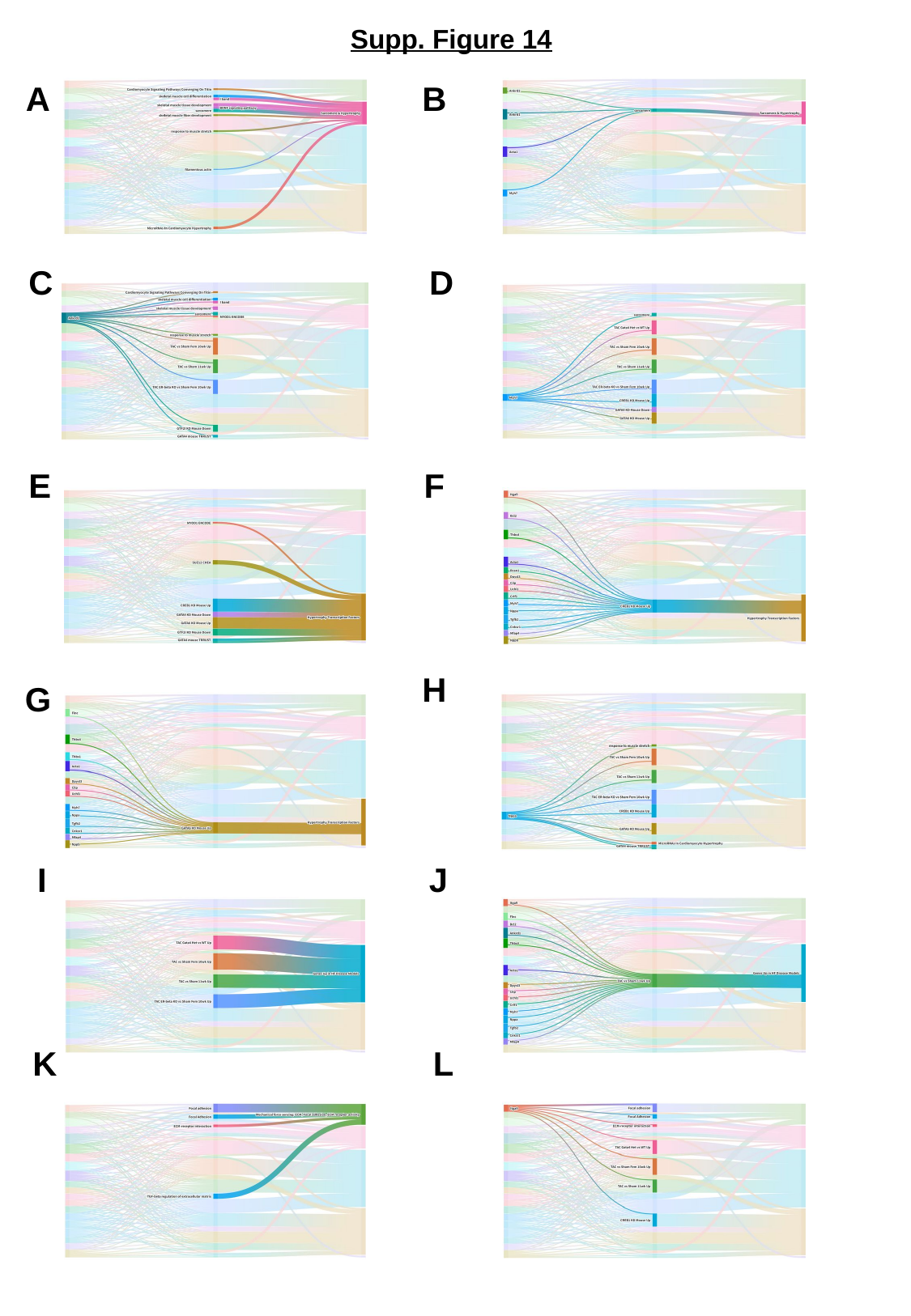

Supp. Figure 14
A
B
C
D
E
F
H
G
I
J
K
L

## Slide 15
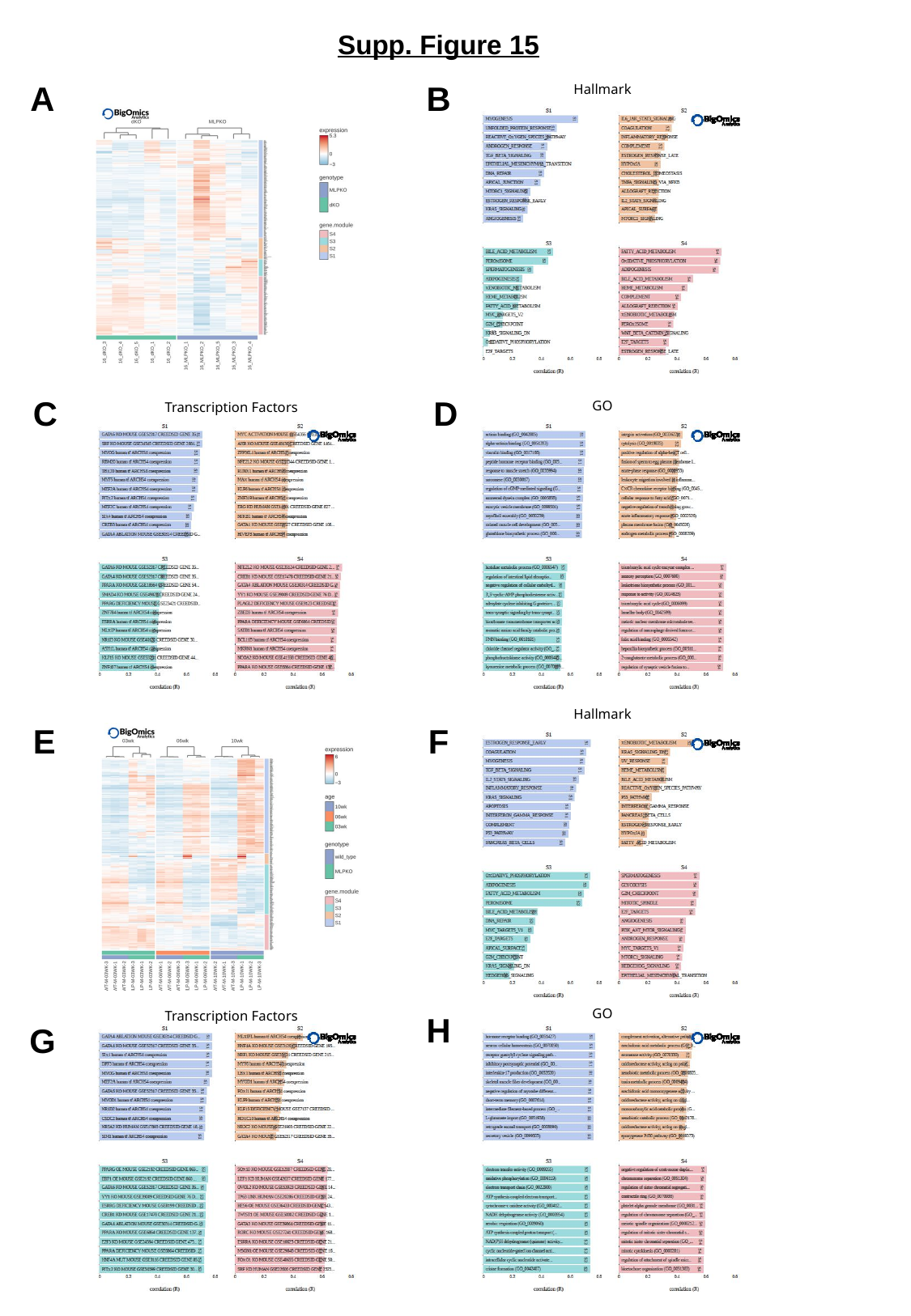

Supp. Figure 15
A
B
Hallmark
C
D
GO
Transcription Factors
Hallmark
E
F
GO
Transcription Factors
H
G
