# Supplementary material for: RRAD-reduction reveals efficacy of targeting L-type calcium channel regulation for treatment of heart failure
Source: Cardiovasc Res. 2025 Oct 1;121(14):2204–21. doi: 10.1093/cvr/cvaf169 (PMC12638741; doi:10.1093/cvr/cvaf169)
Supplement: cvaf169_Supplementary_Data [file cvaf169_supplementary_data.zip › supplemental tables_11_22_2024.pdf]

		Mean Difference	SE	t	Cohen's d	p_{tukey}
dKO F 1	MLPKO F 1	2.885	2.88	1.002	0.346	0.998
	dKO M 1	5.766	2.58	2.239	0.691	0.523
	MLPKO M 1	9.027	2.43	3.718	1.082	0.013
	dKO F 2	-13.96	2.28	-6.122	-1.67	2.313×10^{-7}
	MLPKO F 2	0.19	2.36	0.081	0.023	1
	dKO M 2	-6.925	2.28	-3.038	-0.83	0.104
	MLPKO M 2	9.148	2.32	3.95	1.096	0.006
	dKO F 3	-13.47	2.77	-4.863	-1.61	1.278×10^{-4}
	MLPKO F 3	1.91	3.32	0.575	0.229	1
	dKO M 3	-10.53	3.47	-3.037	-1.26	0.104
	MLPKO M 3	10.745	3.47	3.1	1.288	0.088
MLPKO F 1	dKO M 1	2.88	2.88	1.001	0.345	0.998
	MLPKO M 1	6.141	2.75	2.235	0.736	0.526
	dKO F 2	-16.84	2.62	-6.433	-2.02	4.129×10^{-8}
	MLPKO F 2	-2.695	2.69	-1.003	-0.32	0.998
	dKO M 2	-9.811	2.62	-3.747	-1.18	0.012
	MLPKO M 2	6.262	2.65	2.363	0.751	0.435
	dKO F 3	-16.35	3.05	-5.355	-1.96	1.244×10^{-5}
	MLPKO F 3	-0.975	3.57	-0.273	-0.12	1
	dKO M 3	-13.41	3.7	-3.627	-1.61	0.018
	MLPKO M 3	7.86	3.7	2.126	0.942	0.605
dKO M 1	MLPKO M 1	3.261	2.43	1.343	0.391	0.972
	dKO F 2	-19.72	2.28	-8.652	-2.36	2.044×10^{-13}
	MLPKO F 2	-5.576	2.36	-2.365	-0.67	0.434
	dKO M 2	-12.69	2.28	-5.567	-1.52	4.297×10^{-6}
	MLPKO M 2	3.382	2.32	1.46	0.405	0.95
	dKO F 3	-19.23	2.77	-6.946	-2.31	2.118×10^{-9}
	MLPKO F 3	-3.855	3.32	-1.16	-0.46	0.991
	dKO M 3	-16.29	3.47	-4.7	-1.95	2.655×10^{-4}
	MLPKO M 3	4.979	3.47	1.436	0.597	0.955
MLPKO M 1	dKO F 2	-22.98	2.11	-10.88	-2.76	1.222×10^{-13}
	MLPKO F 2	-8.837	2.2	-4.023	-1.06	0.004
	dKO M 2	-15.95	2.11	-7.554	-1.91	5.206×10^{-11}
	MLPKO M 2	0.121	2.15	0.056	0.014	1
	dKO F 3	-22.49	2.63	-8.544	-2.7	2.461×10^{-13}
	MLPKO F 3	-7.116	3.21	-2.216	-0.85	0.54
	dKO M 3	-19.55	3.36	-5.822	-2.34	1.154×10^{-6}
	MLPKO M 3	1.719	3.36	0.512	0.206	1
dKO F 2	MLPKO F 2	14.146	2.03	6.963	1.695	1.909×10^{-9}
	dKO M 2	7.031	1.94	3.624	0.843	0.018
	MLPKO M 2	23.103	1.98	11.656	2.769	6.284×10^{-14}
	dKO F 3	0.491	2.5	0.197	0.059	1
	MLPKO F 3	15.866	3.1	5.117	1.902	3.939×10^{-5}
	dKO M 3	3.429	3.25	1.054	0.411	0.996
	MLPKO M 3	24.701	3.25	7.593	2.961	4.073×10^{-11}
MLPKO F 2	dKO M 2	-7.115	2.03	-3.502	-0.85	0.027
	MLPKO M 2	8.958	2.07	4.323	1.074	0.001
	dKO F 3	-13.66	2.57	-5.317	-1.64	1.501×10^{-5}
	MLPKO F 3	1.72	3.16	0.545	0.206	1
	dKO M 3	-10.72	3.31	-3.239	-1.28	0.06
	MLPKO M 3	10.555	3.31	3.19	1.265	0.069
dKO M 2	MLPKO M 2	16.073	1.98	8.109	1.926	1.668×10^{-12}
	dKO F 3	-6.54	2.5	-2.62	-0.78	0.274
	MLPKO F 3	8.836	3.1	2.849	1.059	0.166
	dKO M 3	-3.601	3.25	-1.107	-0.43	0.994
	MLPKO M 3	17.671	3.25	5.432	2.118	8.498×10^{-6}
MLPKO M 2	dKO F 3	-22.61	2.53	-8.94	-2.71	1.638×10^{-13}
	MLPKO F 3	-7.237	3.13	-2.314	-0.87	0.47
	dKO M 3	-19.67	3.28	-6.001	-2.36	4.463×10^{-7}
	MLPKO M 3	1.598	3.28	0.487	0.192	1
dKO F 3	MLPKO F 3	15.376	3.48	4.423	1.843	8.722×10^{-4}
	dKO M 3	2.938	3.61	0.813	0.352	1
	MLPKO M 3	24.21	3.61	6.701	2.902	8.899×10^{-9}
MLPKO F 3	dKO M 3	-12.44	4.05	-3.068	-1.49	0.096
	MLPKO M 3	8.835	4.05	2.179	1.059	0.566
dKO M 3	MLPKO M 3	21.272	4.17	5.099	2.55	4.279×10^{-5}

Supplemental Table 1. Post-hoc Tukey's results of longitudinal echocardiography left ventricular fractional shortening (%). The timepoints are 1= 2.5 months of age, 2= 3.5 months (1-month post tamoxifen), and 3= 4.5 months. dKO= double knockout an abbreviated term for the cRAD$^{\Delta/\Delta}$-MLPKO. Note at timepoint 1, dKO is RAD$^{fl/fl}$-MYH6-MerCreMer-MLPKO since tamoxifen was administered after the echo.

		Mean Difference	SE	t	Cohen's d	p_{tukey}
dKO F 1	MLPKO F 1	-0.09	0.19	-0.48	-0.166	1
	dKO M 1	-0.412	0.17	-2.467	-0.761	0.366
	MLPKO M 1	-0.625	0.16	-3.967	-1.154	0.005
	dKO F 2	0.668	0.15	4.515	1.234	5.907×10^{-4}
	MLPKO F 2	0.104	0.15	0.679	0.192	1
	dKO M 2	0.28	0.15	1.894	0.518	0.762
	MLPKO M 2	-0.833	0.15	-5.54	-1.537	4.947×10^{-6}
	dKO F 3	0.776	0.18	4.315	1.432	0.001
	MLPKO F 3	0.001	0.22	0.006	0.002	1
	dKO M 3	0.636	0.23	2.824	1.173	0.177
	MLPKO M 3	-1.067	0.23	-4.742	-1.97	2.201×10^{-4}
MLPKO F 1	dKO M 1	-0.323	0.19	-1.726	-0.596	0.855
	MLPKO M 1	-0.535	0.18	-3.001	-0.988	0.114
	dKO F 2	0.758	0.17	4.46	1.399	7.478×10^{-4}
	MLPKO F 2	0.194	0.17	1.11	0.358	0.994
	dKO M 2	0.37	0.17	2.177	0.683	0.568
	MLPKO M 2	-0.743	0.17	-4.32	-1.372	0.001
	dKO F 3	0.865	0.2	4.366	1.598	0.001
	MLPKO F 3	0.091	0.23	0.393	0.168	1
	dKO M 3	0.725	0.24	3.021	1.339	0.109
	MLPKO M 3	-0.978	0.24	-4.072	-1.805	0.004
dKO M 1	MLPKO M 1	-0.213	0.16	-1.35	-0.393	0.971
	dKO F 2	1.081	0.15	7.301	1.995	2.478×10^{-10}
	MLPKO F 2	0.516	0.15	3.372	0.953	0.04
	dKO M 2	0.693	0.15	4.681	1.279	2.892×10^{-4}
	MLPKO M 2	-0.42	0.15	-2.797	-0.776	0.188
	dKO F 3	1.188	0.18	6.609	2.193	1.514×10^{-8}
	MLPKO F 3	0.414	0.22	1.916	0.764	0.748
	dKO M 3	1.048	0.23	4.656	1.934	3.221×10^{-4}
	MLPKO M 3	-0.655	0.23	-2.91	-1.209	0.144
MLPKO M 1	dKO F 2	1.293	0.14	9.434	2.388	1.604×10^{-13}
	MLPKO F 2	0.729	0.14	5.113	1.346	4.002×10^{-5}
	dKO M 2	0.906	0.14	6.605	1.672	1.553×10^{-8}
	MLPKO M 2	-0.208	0.14	-1.487	-0.383	0.943
	dKO F 3	1.401	0.17	8.197	2.586	1.006×10^{-12}
	MLPKO F 3	0.626	0.21	3.005	1.156	0.113
	dKO M 3	1.261	0.22	5.782	2.327	1.425×10^{-6}
	MLPKO M 3	-0.442	0.22	-2.028	-0.816	0.674
dKO F 2	MLPKO F 2	-0.564	0.13	-4.279	-1.042	0.002
	dKO M 2	-0.388	0.13	-3.08	-0.716	0.093
	MLPKO M 2	-1.501	0.13	-11.67	-2.771	6.239×10^{-14}
	dKO F 3	0.107	0.16	0.663	0.198	1
	MLPKO F 3	-0.667	0.2	-3.313	-1.231	0.048
	dKO M 3	-0.033	0.21	-0.155	-0.06	1
	MLPKO M 3	-1.736	0.21	-8.217	-3.204	9.003×10^{-13}
MLPKO F 2	dKO M 2	0.176	0.13	1.338	0.326	0.973
	MLPKO M 2	-0.937	0.14	-6.964	-1.729	1.903×10^{-9}
	dKO F 3	0.672	0.17	4.029	1.24	0.004
	MLPKO F 3	-0.103	0.21	-0.501	-0.19	1
	dKO M 3	0.532	0.22	2.475	0.981	0.361
	MLPKO M 3	-1.171	0.22	-5.452	-2.162	7.670×10^{-6}
dKO M 2	MLPKO M 2	-1.113	0.13	-8.65	-2.055	2.043×10^{-13}
	dKO F 3	0.495	0.16	3.056	0.914	0.099
	MLPKO F 3	-0.279	0.2	-1.386	-0.515	0.965
	dKO M 3	0.355	0.21	1.682	0.656	0.875
	MLPKO M 3	-1.348	0.21	-6.381	-2.488	5.540×10^{-8}
MLPKO M 2	dKO F 3	1.609	0.16	9.795	2.969	1.587×10^{-13}
	MLPKO F 3	0.834	0.2	4.108	1.54	0.003
	dKO M 3	1.468	0.21	6.898	2.711	2.807×10^{-9}
	MLPKO M 3	-0.234	0.21	-1.101	-0.433	0.994
dKO F 3	MLPKO F 3	-0.774	0.23	-3.431	-1.43	0.033
	dKO M 3	-0.14	0.24	-0.598	-0.259	1
	MLPKO M 3	-1.843	0.24	-7.857	-3.402	7.755×10^{-12}
MLPKO F 3	dKO M 3	0.634	0.26	2.41	1.171	0.403
	MLPKO M 3	-1.069	0.26	-4.06	-1.973	0.004
dKO M 3	MLPKO M 3	-1.703	0.27	-6.287	-3.144	9.345×10^{-8}

Supplemental Table 2. Post-hoc Tukey's results of longitudinal echocardiography left ventricular internal diameter; diastolic (mm). The timepoints are 1= 2.5 months of age, 2= 3.5 months (1-month post tamoxifen), and 3= 4.5 months. dKO= double knockout an abbreviated term for the cRAD$^{\Delta/\Delta}$-MLPKO. Note at timepoint 1, dKO is RAD$^{fl/fl}$-MYH6-MerCreMer-MLPKO since tamoxifen was administered after the echo.

		Mean Difference	SE	t	Cohen's d	p_{tukey}
dKO F 1	MLPKO F 1	0.025	0.02	1.109	0.383	0.994
	dKO M 1	0.031	0.02	1.511	0.466	0.936
	MLPKO M 1	0.04	0.02	2.043	0.594	0.664
	dKO F 2	-0.058	0.02	-3.195	-0.873	0.068
	MLPKO F 2	0.017	0.02	0.911	0.257	0.999
	dKO M 2	-0.032	0.02	-1.768	-0.483	0.834
	MLPKO M 2	0.05	0.02	2.688	0.746	0.239
	dKO F 3	-0.06	0.02	-2.731	-0.906	0.218
	MLPKO F 3	0.033	0.03	1.233	0.491	0.986
	dKO M 3	-0.09	0.03	-3.26	-1.355	0.056
	MLPKO M 3	0.076	0.03	2.748	1.142	0.209
MLPKO F 1	dKO M 1	0.006	0.02	0.243	0.084	1
	MLPKO M 1	0.014	0.02	0.643	0.212	1
	dKO F 2	-0.084	0.02	-4.002	-1.256	0.005
	MLPKO F 2	-0.008	0.02	-0.389	-0.125	1
	dKO M 2	-0.058	0.02	-2.759	-0.866	0.205
	MLPKO M 2	0.024	0.02	1.144	0.363	0.992
	dKO F 3	-0.086	0.02	-3.522	-1.289	0.025
	MLPKO F 3	0.007	0.03	0.254	0.109	1
	dKO M 3	-0.116	0.03	-3.919	-1.737	0.006
	MLPKO M 3	0.051	0.03	1.713	0.759	0.861
dKO M 1	MLPKO M 1	0.009	0.02	0.44	0.128	1
	dKO F 2	-0.089	0.02	-4.903	-1.339	1.068×10^{-4}
	MLPKO F 2	-0.014	0.02	-0.74	-0.209	1
	dKO M 2	-0.063	0.02	-3.475	-0.95	0.029
	MLPKO M 2	0.019	0.02	1.007	0.28	0.997
	dKO F 3	-0.091	0.02	-4.136	-1.373	0.003
	MLPKO F 3	0.002	0.03	0.062	0.025	1
	dKO M 3	-0.121	0.03	-4.383	-1.821	0.001
	MLPKO M 3	0.045	0.03	1.626	0.675	0.898
MLPKO M 1	dKO F 2	-0.098	0.02	-5.797	-1.467	1.312×10^{-6}
	MLPKO F 2	-0.022	0.02	-1.28	-0.337	0.981
	dKO M 2	-0.072	0.02	-4.257	-1.077	0.002
	MLPKO M 2	0.01	0.02	0.588	0.152	1
	dKO F 3	-0.1	0.02	-4.756	-1.501	2.070×10^{-4}
	MLPKO F 3	-0.007	0.03	-0.268	-0.103	1
	dKO M 3	-0.13	0.03	-4.841	-1.949	1.411×10^{-4}
	MLPKO M 3	0.036	0.03	1.36	0.548	0.97
dKO F 2	MLPKO F 2	0.075	0.02	4.643	1.13	3.412×10^{-4}
	dKO M 2	0.026	0.02	1.677	0.39	0.877
	MLPKO M 2	0.108	0.02	6.815	1.619	4.590×10^{-9}
	dKO F 3	-0.002	0.02	-0.111	-0.033	1
	MLPKO F 3	0.091	0.03	3.67	1.364	0.015
	dKO M 3	-0.032	0.03	-1.235	-0.481	0.986
	MLPKO M 3	0.134	0.03	5.168	2.015	3.087×10^{-5}
MLPKO F 2	dKO M 2	-0.049	0.02	-3.041	-0.741	0.103
	MLPKO M 2	0.033	0.02	1.967	0.489	0.715
	dKO F 3	-0.078	0.02	-3.78	-1.164	0.01
	MLPKO F 3	0.016	0.03	0.617	0.234	1
	dKO M 3	-0.107	0.03	-4.065	-1.612	0.004
	MLPKO M 3	0.059	0.03	2.23	0.884	0.529
dKO M 2	MLPKO M 2	0.082	0.02	5.173	1.229	3.003×10^{-5}
	dKO F 3	-0.028	0.02	-1.414	-0.423	0.96
	MLPKO F 3	0.065	0.03	2.621	0.974	0.274
	dKO M 3	-0.058	0.03	-2.235	-0.871	0.526
	MLPKO M 3	0.108	0.03	4.168	1.625	0.002
MLPKO M 2	dKO F 3	-0.11	0.02	-5.45	-1.652	7.780×10^{-6}
	MLPKO F 3	-0.017	0.03	-0.68	-0.255	1
	dKO M 3	-0.14	0.03	-5.345	-2.1	1.304×10^{-5}
	MLPKO M 3	0.026	0.03	1.008	0.396	0.997
dKO F 3	MLPKO F 3	0.093	0.03	3.354	1.397	0.042
	dKO M 3	-0.03	0.03	-1.035	-0.448	0.997
	MLPKO M 3	0.136	0.03	4.73	2.048	2.326×10^{-4}
MLPKO F 3	dKO M 3	-0.123	0.03	-3.798	-1.846	0.01
	MLPKO M 3	0.043	0.03	1.339	0.651	0.973
dKO M 3	MLPKO M 3	0.166	0.03	4.993	2.496	7.043×10^{-5}

Supplemental Table 3. Post-hoc Tukey's results of longitudinal echocardiography wall to chamber ratio (hr ratio). The timepoints are 1= 2.5 months of age, 2= 3.5 months (1-month post tamoxifen), and 3= 4.5 months. dKO= double knockout an abbreviated term for the cRAD$^{\Delta/\Delta}$-MLPKO. Note at timepoint 1, dKO is RAD$^{fl/fl}$-MYH6-MerCreMer-MLPKO since tamoxifen was administered after the echo. The anterior and posterior wall were averaged and divided by left ventricular internal diameter (diastolic).

genotype	MLPKO	MLPKO	MLPKO	cRADΔ/Δ MLPKO	cRADΔ/Δ MLPKO	cRADΔ/Δ MLPKO
timepoint	2.5 mo.	3.5 mo.	4.5 mo.	2.5 mo.	3.5 mo.	4.5 mo.
Weight_mean	27.35	27.28	27.61	25.78	28.05	28.17
Weight_median	28.00	28.00	26.00	26.00	28.50	25.00
Weight_sem	0.68	0.66	1.13	0.66	0.54	1.31
FractionalShortening_mean	19.15	21.20	20.01	23.19	36.52	38.56
FractionalShortening_median	18.70	19.27	19.80	20.31	35.18	37.95
FractionalShortening_sem	1.04	1.14	1.62	1.39	1.22	1.65
Diameter_s_mean	3.75	3.64	3.76	3.39	2.38	2.12
Diameter_s_median	3.77	3.68	3.50	3.41	2.45	2.12
Diameter_s_sem	0.12	0.12	0.20	0.12	0.08	0.08
Diameter_d_mean	4.60	4.54	4.66	4.36	3.68	3.43
Diameter_d_median	4.73	4.57	4.50	4.38	3.75	3.30
Diameter_d_sem	0.10	0.09	0.17	0.09	0.06	0.07
Volume_s_mean	64.28	61.61	65.01	50.77	22.68	15.83
Volume_s_median	60.80	57.55	51.23	47.93	21.30	14.73
Volume_s_sem	4.86	4.21	8.51	3.74	1.76	1.63
Volume_d_mean	100.15	98.53	103.38	88.47	59.46	49.32
Volume_d_median	103.88	96.08	92.51	87.05	60.01	44.32
Volume_d_sem	5.09	4.64	8.87	4.17	2.31	2.58
StrokeVolume_mean	35.87	36.91	38.37	37.70	36.78	33.49
StrokeVolume_median	34.32	35.35	37.50	37.94	37.14	31.57
StrokeVolume_sem	1.13	0.99	1.66	1.35	0.97	1.59
EjectionFraction_mean	38.98	42.07	40.47	45.60	65.52	68.95
EjectionFraction_median	38.79	39.84	40.91	41.99	65.40	69.54
EjectionFraction_sem	1.89	1.87	2.93	2.14	1.58	2.07
HeartRate_mean	451.05	462.81	439.51	447.06	499.09	528.58
HeartRate_median	448.88	464.61	421.99	441.22	498.40	522.31
HeartRate_sem	10.48	9.46	17.75	10.44	7.72	11.68
CardiacOutput_mean	16.06	17.15	16.67	17.03	18.26	17.62
CardiacOutput_median	14.78	17.05	16.01	16.55	18.32	16.43
CardiacOutput_sem	0.57	0.59	0.75	0.82	0.54	0.89
LVMass_mean	150.24	146.56	140.07	143.88	128.34	121.95
LVMass_median	147.83	148.18	134.49	146.88	123.01	124.71
LVMass_sem	5.18	4.95	7.94	4.10	3.49	6.57
LVMassCor_mean	120.19	117.25	112.05	115.10	102.67	97.56
LVMassCor_median	118.27	118.55	107.59	117.51	98.41	99.77
LVMassCor_sem	4.14	3.96	6.35	3.28	2.79	5.25
LVAW_s_mean	1.15	1.14	1.07	1.24	1.50	1.52
LVAW_s_median	1.22	1.13	1.00	1.21	1.53	1.54
LVAW_s_sem	0.05	0.04	0.05	0.05	0.03	0.06
LVAW_d_mean	0.87	0.86	0.78	0.91	0.99	0.98
LVAW_d_median	0.90	0.85	0.78	0.91	0.97	1.01
LVAW_d_sem	0.03	0.02	0.02	0.03	0.02	0.04
LVPW_s_mean	0.92	0.93	0.90	0.99	1.24	1.37
LVPW_s_median	0.89	0.87	0.90	0.95	1.23	1.42
LVPW_s_sem	0.03	0.03	0.03	0.04	0.03	0.05
LVPW_d_mean	0.75	0.74	0.72	0.77	0.88	0.96
LVPW_d_median	0.73	0.74	0.75	0.76	0.86	0.97
LVPW_d_sem	0.02	0.02	0.02	0.03	0.02	0.04
HrRatio_mean	0.18	0.18	0.16	0.20	0.26	0.29
HrRatio_median	0.17	0.17	0.16	0.18	0.25	0.29
HrRatio_sem	0.01	0.01	0.01	0.01	0.01	0.01
LVMass_BW_mean	5.56	5.22	5.07	5.50	4.71	4.42
LVMass_BW_median	5.57	5.21	4.97	5.64	4.71	4.17
LVMass_BW_sem	0.14	0.13	0.18	0.13	0.12	0.22
N (mice)	41	65	17	42	74	24

Supplemental Table 4. Descriptive statistics for longitudinal echocardiography at each timepoint. Mice were born either MLPKO-RAD$^{fl/fl}$ or MLPKO-RAD$^{fl/fl}$ MYH6-MerCreMer. A single dose of tamoxifen induced cardiomyocyte-specific RAD deletion (cRAD$^{\Delta/\Delta}$) at 2.5-months in mice with the MYH6-MerCreMer transgene. Longitudinal echocardiography on pre-tamoxifen (2.5 mo old) and 1- and 2-months post-tamoxifen (3.5- and 4.5-month-old) mice. These numbers represent pooled sex.

	FS (%)	LVID;d (mm)	LVPW;d (mm)	Age at measurement (months)	Sex	N
**Published MLP$^{+/+}$**						
**mean**	44.1 ±4.2	3.47 ±0.17	0.58 ±0.15			
Makarewich et al.	59	2.8		1.8	male	5-15
Li et al.	36.6 ±0.9	3.43 ±0.09	0.93 ±0.05	2.3	pooled	7
Arber et al.		3.9 ±0.1	0.64 ±0.02	2.3	n.r.	10
Holmes et al.	36.3 ±1.8	3.42 ±0.06	0.77 ±.03	2.3	pooled	16
Esposito et al.	47 ±2.8	3.6 ±0.04	0.6 ±0.03	5-7	pooled	10
Yamamoto et al.	41.7 ±1.4	3.66 ±0.08	0.52 ±0.03	5.5	n.r.	16
**published MLPKO**						
**mean**	19.3 ±2.1	4.54 ±0.11	0.616 ±0.05			
Makarewich et al.	18	4.3		1.8	male	5-15
Li et al.	14.5 ±1.7	4.52 ±0.12	0.71 ±0.03	2.3	pooled	7
Arber et al.		4.97 ±0.2	0.51 ±0.02	2.3	n.r.	9
Holmes et al.	16.4 ±1.5	4.19 ±0.07	0.74 ±0.03	2.3	pooled	10
Esposito et al.	26 ±2	4.7 ±0.19	0.6 ±0.03	5-7	pooled	10
Yamamoto et al.	21.8 ±1.3	4.54 ±0.1	0.52 ±0.04	5.5	n.r.	16
**published protection MLPKO**						
**mean**	40.0 ±1.8	3.58 ±0.15	0.84 ±0.19			
Makarewich et al. (DWORF MLPKO)	48	3.3		1.8	male	5-15
Li et al. (MYBPC3KO MLPKO)	29.0 ±1.9	3.36 ±0.16	1.18 ±0.05	2.3	pooled	7
Esposito et al. (βARKct MLPKO)	53 ±3.1	3.7 ±0.13	0.8 ±0.07	5-7	pooled	10
Yamamoto et al. (AT1aKO MLPKO)	30.2 ±1.1	3.96 ±0.09	0.54 ±0.03	5.5	n.r.	16

Supplemental Table 5. Summary data for published muscle lim protein knockout (MLPKO) studies. The means of each group were used in Supp. Figure 2. The third grouping represents transgenic overexpression or constitutive knockout mice. Means with SEM are reported. “n.r.” for sex indicates the study did not report which sex was used nor if mice were pooled. Arber et al. 1997 (DOI: 10.1016/S0092-8674(00)81878-4), Esposito et al. 2000 (DOI: 10.1152/ajpheart.2000.279.6.H3101), Yamamoto et al. 2007 (DOI: 10.1253/circj.71.1958), Makarewich et al. 2018 (DOI: 10.7554/eLife.38319), Li et al. 2018 (DOI: 10.1016/j.ijcard.2018.09.073), Holmes et al. 2023 (DOI: 10.1152/ajpheart.00130.2023).

CaT Amplitude

Primary Factor	Secondary Factor	*N*	Mean	Standard Error
vehicle	control	16	0.369	0.037
	anti-RRAD	16	0.708	0.038
iso.	control	16	0.657	0.035
	anti-RRAD	12	0.667	0.030

Note. N_{Total} = 60.

Estimated Marginal Means ▼

Row	shRNA treatment	drug treatment	Estimate	SE	95% CI	
					Lower	Upper
1	control	vehicle	0.371	0.049	0.275	0.467
2	anti-RRAD	vehicle	0.714	0.050	0.615	0.812
3	control	iso.	0.669	0.062	0.547	0.792
4	anti-RRAD	iso.	0.655	0.058	0.541	0.769

CaT Upstroke Velocity

Primary Factor	Secondary Factor	*N*	Mean	Standard Error
vehicle	control	16	25.103	1.070
	anti-RRAD	16	38.016	1.760
iso.	control	16	40.567	1.438
	anti-RRAD	12	39.526	1.560

Note. N_{Total} = 60.

Estimated Marginal Means ▼

Row	shRNA treatment	drug treatment	Estimate	SE	95% CI	
					Lower	Upper
1	control	vehicle	24.978	1.409	22.216	27.740
2	anti-RRAD	vehicle	38.206	3.774	30.809	45.603
3	control	iso.	40.848	2.110	36.713	44.982
4	anti-RRAD	iso.	39.253	2.319	34.708	43.798

CaT Decay Velocity

Primary Factor	Secondary Factor	*N*	Mean	Standard Error
vehicle	control	16	1.386	0.047
	anti-RRAD	16	2.494	0.120
iso.	control	16	3.065	0.133
	anti-RRAD	12	2.893	0.117

Note. N_{Total} = 60.

Estimated Marginal Means ▼

Row	shRNA treatment	drug treatment	Estimate	SE	95% CI	
					Lower	Upper
1	control	vehicle	1.384	0.081	1.225	1.543
2	anti-RRAD	vehicle	2.474	0.223	2.038	2.911
3	control	iso.	3.038	0.300	2.450	3.625
4	anti-RRAD	iso.	2.905	0.105	2.699	3.111

Force Amplitude ▼

Primary Factor	Secondary Factor	*N*	Mean	95% CI for Mean Difference	
				Lower	Upper
vehicle	control	21	13.378	12.863	13.893
	anti-RRAD	21	24.393	23.968	24.817
iso.	control	21	23.412	22.319	24.505
	anti-RRAD	16	25.428	24.878	25.978

Note. N_{Total} = 79.
Interval around mean represents 95% confidence interval.

Estimated Marginal Means

Row	shRNA treatment	drug treatment	Estimate	SE	95% CI	
					Lower	Upper
1	control	vehicle	13.297	0.344	12.623	13.971
2	anti-RRAD	vehicle	24.529	0.455	23.636	25.421
3	control	iso.	23.909	1.379	21.207	26.612
4	anti-RRAD	iso.	25.442	0.338	24.779	26.105

Contraction Max Velocity

Primary Factor	Secondary Factor	*N*	Mean	95% CI for Mean Difference	
				Lower	Upper
vehicle	control	21	15.712	14.595	16.830
	anti-RRAD	21	22.722	22.048	23.397
iso.	control	21	21.728	21.237	22.219
	anti-RRAD	16	22.652	22.127	23.177

Note. N_{Total} = 79.
Interval around mean represents 95% confidence interval.

Estimated Marginal Means ▼

Row	shRNA treatment	drug treatment	Estimate	SE	95% CI	
					Lower	Upper
1	control	vehicle	15.742	0.363	15.030	16.454
2	anti-RRAD	vehicle	22.777	0.389	22.015	23.538
3	control	iso.	21.832	0.475	20.901	22.763
4	anti-RRAD	iso.	22.671	0.527	21.639	23.704

Relaxation Max Velocity

Primary Factor	Secondary Factor	*N*	Mean	95% CI for Mean Difference	
				Lower	Upper
vehicle	control	21	2.075	1.940	2.210
	anti-RRAD	21	3.033	2.844	3.222
iso.	control	21	3.323	3.080	3.565
	anti-RRAD	16	3.245	3.056	3.435

Note. N_{Total} = 79.

Estimated Marginal Means ▼

Row	shRNA treatment	drug treatment	Estimate	SE	95% CI	
					Lower	Upper
1	control	vehicle	2.078	0.128	1.827	2.329
2	anti-RRAD	vehicle	3.036	0.126	2.790	3.282
3	control	iso.	3.317	0.201	2.924	3.711
4	anti-RRAD	iso.	3.263	0.180	2.911	3.615

Supplemental Table 6. Descriptive statistics and mixed model ANOVA estimated marginal means of ShRNA *RRAD* knockdown of human heart failure with reduced ejection fraction ex vivo heart slices calcium (CaT) transients and force recordings acutely treated with or without acute isoproterenol (iso.).

Term	hits	size	p	q	Combined .Score	Genes	database	Gene_Ratio	log2_score	original DB	full_term
Dilated cardiomyopathy	7	100	7E-05	4E-02	72.2	CACNB1;PRKAB2;ITGA3;CACNA2D1;ADCY1;ITGA9;MYH7	WikiPath & BioPlan	0.07	6.17	BioPlanet	Dilated cardiomyopathy
Stress induction of HSP regulation	3	15	4E-04	7E-02	191.8	ACTA1;BCL2;HSPB1	WikiPath & BioPlan	0.20	7.58	BioPlanet	Stress induction of HSP regulation
Prion pathway	3	21	1E-03	8E-02	110.9	PRNP;LAMC3;BCL2	WikiPath & BioPlan	0.14	6.79	BioPlanet	Prion pathway
Opening of calcium channels triggered by depolarization of the presynaptic terminal	2	12	6E-03	2E-01	99.7	CACNB1;CACNA2D1	WikiPath & BioPlan	0.17	6.64	BioPlanet	Opening of calcium channels triggered by depolarization of the presynaptic terminal
MYOD1 ENCODE	8	166	3E-04	3E-02	41.3	CACNB1;SYNPO2L;TNNT1;MEIG1;ANKRD1;HSPB1;TRIM16;KLHL40	TF Consensus	0.05	5.37	ENCODE_an	MYOD1 ENCODE
SUZ12 CHEA	31	1684	9E-04	4E-02	13.9	SLC22A4;SHC4;SEMA3C;ABCB4;SIX1;HR;ADCY1;LRP8;ADORA1;KLRG2;FLNC;ADAMTS8;DUSP4;EGR3;CACNA2D1;SHISA3;SYPL2;POU3F1;KLHL40;ADAMTS20;CNKSR2;GNAO1;TBX15;RBP4;FRAS1;SYT12;DOK5;BCL2;ASTN2;SERINC2;SNTB1	TF Consensus	0.02	3.80	ENCODE_an	SUZ12 CHEA
FOSL2 ENCODE	6	196	2E-02	4E-01	13.2	SLC22A4;SHC4;NUAK1;ANKRD1;KLHL41;AZIN1	TF Consensus	0.03	3.72	ENCODE_an	FOSL2 ENCODE
SMAD4 CHEA	13	584	7E-03	2E-01	11.5	BLK;ATP8A2;NRG1;CES5A;AMOT;GNAO1;UCK2;FRAS1;BAMBI;KIF5B;SLC38A2;MAP3K14;ITGA9	TF Consensus	0.02	3.52	ENCODE_an	SMAD4 CHEA
skeletal muscle cell differentiation	4	12	5E-06	2E-03	609.1	ANKRD1;ANKRD2;KLHL40;KLHL41	GO BP	0.33	9.25	GO_Biolog	skeletal muscle cell differentiation (GO:0035914)
skeletal muscle thin filament assembly	3	10	1E-04	2E-02	383.8	ACTA1;MYOM2;MYOM3	GO BP	0.30	8.58	GO_Biolog	skeletal muscle thin filament assembly (GO:0030240)
skeletal muscle tissue development	7	39	1E-07	2E-04	352.1	ACTA1;CSRP3;ANKRD1;SIX1;ANKRD2;KLHL40;KLHL41	GO BP	0.18	8.46	GO_Biolog	skeletal muscle tissue development (GO:0007519)
skeletal myofibril assembly	3	12	2E-04	2E-02	279.0	ACTA1;MYOM2;MYOM3	GO BP	0.25	8.12	GO_Biolog	skeletal myofibril assembly (GO:0014866)
I band	3	15	4E-04	3E-02	191.8	ANKRD1;ANKRD2;KLHL40	GO CC	0.20	7.58	GO_Cellul	I band (GO:0031674)
M band	3	17	6E-04	3E-02	156.2	MYOM2;KLHL41;MYOM3	GO CC	0.18	7.29	GO_Cellul	M band (GO:0031430)
sarcomere	4	27	2E-04	2E-02	152.1	ACTA1;ANKRD1;ANKRD2;MYH7	GO CC	0.15	7.25	GO_Cellul	sarcomere (GO:0030017)
muscle myosin complex	3	24	2E-03	5E-02	89.4	MYOM2;MYOM3;MYH7	GO CC	0.13	6.48	GO_Cellul	muscle myosin complex (GO:0005859)
ECM-receptor interaction	8	88	3E-06	6E-04	128.1	FRAS1;SV2B;LAMC3;ITGA3;COL4A3;COL9A2;FREM1;ITGA9	KEGG	0.09	7.00	KEGG_2021	ECM-receptor interaction
Hypertrophic cardiomyopathy	6	90	3E-04	2E-02	57.7	CACNB1;PRKAB2;ITGA3;CACNA2D1;ITGA9;MYH7	KEGG	0.07	5.85	KEGG_2021	Hypertrophic cardiomyopathy
Focal adhesion	10	201	4E-05	4E-03	53.7	SHC4;LAMC3;ITGA3;COL4A3;BCL2;PAK6;COL9A2;FLNC;MYL12A;ITGA9	KEGG	0.05	5.75	KEGG_2021	Focal adhesion
Dilated cardiomyopathy	6	96	4E-04	2E-02	51.5	CACNB1;ITGA3;CACNA2D1;ADCY1;ITGA9;MYH7	KEGG	0.06	5.69	KEGG_2021	Dilated cardiomyopathy
MGS717 Murine age10wk left ventricle female transverse aortic constriction v sham GSE18224 up	45	183	5E-50	5E-47	4604.3	SLC22A4;CLIC5;HSPB6;CCDC68;ABCB4;PTPRN;PRUNE2;NLRC3;MYL12A;IL18BP;FSTL3;MMP23;FGF6;NUAK1;UCHL1;KIF5B;NRAP;ANKRD1;NUDT18;RRP12;FLNC;PLET1OS;GAS2L3;LMAN1L;MEIG1;SHISA4;TMEM62;KLHL40;SORL1;HSP25-PS1;ENAH;TTLL7;RCAN1;ACTA1;TBX15;UCK2;SYNPO2L;DCT;BAMBI;COL4A3;OTULIN;BCL2;LRP11;ITGA9;MYH7	SysMyo Muscle	0.25	12.17	SysMyo_Mu	MGS717 Murine age10wk left ventricle female transverse aortic constriction v sham GSE18224 up
MGS715 Murine age10wk left ventricle female transverse aortic constriction estrogen receptor beta KO v sham GSE18224 up	39	181	8E-41	3E-38	3039.8	HSPB6;CCDC68;PTPRN;SETD7;PRUNE2;NLRC3;MYL12A;IL18BP;FSTL3;MMP23;FGF6;NUAK1;UCHL1;NRAP;ANKRD1;NUDT18;RRP12;GAS2L3;PMM1;LMAN1L;GDF15;SHISA4;KLHL40;SORL1;HSP25-PS1;ENAH;RCAN1;ACTA1;TBX15;UCK2;TUBB2A;SYNPO2L;BAMBI;OTULIN;MAFK;SPRR1A;PFKP;LRP11;MYH7	SysMyo Muscle	0.22	11.57	SysMyo_Mu	MGS715 Murine age10wk left ventricle female transverse aortic constriction estrogen receptor beta KO v sham GSE18224 up
MGS1503 Murine ventricle transverse aortic constriction Gata4 heterozygotes v WT GSE5500 up	40	204	4E-40	1E-37	2664.7	SLC22A4;SEMA3C;CCDC68;FHL1;SEMA3B;FSTL3;AMOT;FGF6;NUAK1;UCHL1;NMRK2;NUDT18;ANKRD2;RRP12;FLNC;PLET1OS;PHLDA1;SCN1B;PRKAB2;LMAN1L;GSTO1;GDF15;TMEM62;SHISA4;KLHL40;HSP25-PS1;ENAH;SPCS3;RCAN1;ACTA1;UCK2;TUBB2A;SYNPO2L;OTULIN;BCL2;MAFK;ATP13A3;PFKP;ITGA9;MYH7	SysMyo Muscle	0.20	11.38	SysMyo_Mu	MGS1503 Murine ventricle transverse aortic constriction Gata4 heterozygotes v WT GSE5500 up
MGS731 Murine age11wk myocardium aortic banding failing v sham GSE36074 up	33	159	5E-34	1E-31	2321.7	CLIC5;HSPB6;SERPINE1;FHL1;NLRC3;MYL12A;UCHL1;NMRK2;KIF5B;NRAP;ANKRD1;FLNC;PRKAB2;LMAN1L;GDF15;SHISA3;ENAH;TTLL7;ACTA1;TBX15;SPCS3;UCK2;STAR;SYNPO2L;OTULIN;NAPEPLD;BCL2;MAFK;TRIM16;PFKP;LRP11;ITGA9;MYH7	SysMyo Muscle	0.21	11.18	SysMyo_Mu	MGS731 Murine age11wk myocardium aortic banding failing v sham GSE36074 up
CREB1 KD Mouse Up	39	346	2E-29	3E-26	999.8	SLC22A4;SHC4;TFRC;CCDC68;PTPRN;FSTL3;AMOT;FGF6;NUAK1;UCHL1;NMRK2;NUDT18;RRP12;PHLDA1;SCN1B;GAS2L3;EGR3;LMAN1L;GDF15;SLC10A3;KCNJ14;RRAS2;TMEM62;RAB27B;SHISA3;ETV5;ENAH;SLC7A5;RCAN1;ACTA1;SYNPO2L;COL4A3;BCL2;LNX1;POLR1E;MAFK;LRP11;ITGA9;MYH7	TF Pertubations	0.11	9.97	TF_Pertur	CREB1 KD MOUSE GSE17478 CREEDSID GENE 2158 UP
GATA6 KO Mouse Up	21	225	1E-14	1E-11	353.0	ATP8A2;CCDC68;ABCB4;SHISA4;HSPB1;FHL3;SIX1;NLRC3;LRRC55;AMOT;ADAMTS20;RIMS2;ACTA1;UCHL1;XIRP2;FLNC;ATP13A3;ASTN2;SPRR1A;PHLDA1;MYH7	TF Pertubations	0.09	8.46	TF_Pertur	GATA6 KO MOUSE GSE52317 CREEDSID GENE 357 UP
GATA4 KO Mouse Down	19	229	2E-12	1E-09	258.1	SLC22A4;PRKAB2;GSTO1;SERPINE1;PRUNE2;SEMA3B;SHISA4;DAPP1;SHISA3;LRP8;FGF6;CNN1;ACTA1;UCK2;STAR;ANKRD2;MUC15;SERINC2;MYH7	TF Pertubations	0.08	8.01	TF_Pertur	GATA4 ABLATION MOUSE GSE30314 CREEDSID GENE 838 DOWN
SRF MUT Mouse Down	18	219	1E-11	5E-09	239.8	DDR1;HSPB6;SERPINE1;ANKRD23;FHL1;FHL3;MYOM2;SPCS3;RCAN1;CNN1;ACTA1;CSRP3;SYNPO2L;ANKRD1;PKP2;PHLDA1;SLC38A2;MYH7	TF Pertubations	0.08	7.91	TF_Pertur	SRF MUT MOUSE GSE3181 CREEDSID GENE 851 DOWN
GATA4 mouse TRRUST	4	22	7E-05	1E-02	212.8	STAR;ANKRD1;ADORA1;LRRC10	TF Consensus	0.18	7.73	TRRUST_Tr	GATA4 mouse
SMAD4 human TRRUST	4	26	1E-04	1E-02	161.8	BAMBI;SERPINE1;BCL2;FSTL3	TF Consensus	0.15	7.34	TRRUST_Tr	SMAD4 human
NR4A1 human	2	15	1E-02	2E-01	69.9	STAR;SERPINE1	TF Consensus	0.13	6.13	TRRUST_Tr	NR4A1 human
SMAD3 human	3	31	4E-03	2E-01	59.1	BAMBI;SERPINE1;FSTL3	TF Consensus	0.10	5.89	TRRUST_Tr	SMAD3 human
Cardiomyocyte Signaling Pathways Converging On Titin	5	33	2E-05	5E-03	193.5	CSRP3;FHL1;ANKRD23;ANKRD1;ANKRD2	WikiPath & BioPlan	0.15	7.60	WikiPathw	Cardiomyocyte Signaling Pathways Converging On Titin WP5344
Focal Adhesion	9	199	2E-04	3E-02	40.7	BLK;SHC4;LAMC3;ITGA3;BCL2;PAK6;FLNC;MYL12A;ITGA9	WikiPath & BioPlan	0.05	5.35	WikiPathw	Focal Adhesion WP306
Fas Ligand Pathway And Stress Induction Of Heat Shock Proteins	4	43	9E-04	6E-02	71.1	ACTA1;PRKDC;BCL2;HSPB1	WikiPath & BioPlan	0.09	6.15	WikiPathw	Fas Ligand Pathway And Stress Induction Of Heat Shock Proteins WP314
Iron Metabolism Disorders	2	17	1E-02	3E-01	57.4	TFRC;CYBRD1	WikiPath & BioPlan	0.12	5.84	WikiPathw	Iron Metabolism Disorders WP5172

Supplemental Table 7. Singular enrichment analysis of bulk RNAseq differentially expressed downregulated genes of hearts from 5 MLPKO and 5 cRAD$^{\Delta/\Delta}$-MLPKO 3.5-month old male mice (1 month after cRAD$^{\Delta/\Delta}$ induction). Databases queried included experimental model databases (Muscle Gene Set, TF Pertubations), GO, transcription factors (TF Consensus of ENCODE & CHEA, and TRRUST) and pathway databases (Wiki Pathways, BioPlanet, KEGG). Analysis was performed using Enrichr. The genes columns includes all hits of DE downregulated genes for a given term (p-adj <0.05, log_2 fold-change < -0.5) (DESeq2).

Term	hits	size	P.value	Adjusted.P .value	Combined. Score	Genes	database	Gene_Ratio	log2_score	Original database	full_term
SUZ12 CHEA	26	1684	1E-04	1E-02	21.6	RTN4R;PTPRU;TNXB;RGS2;RASSF2;FGF9;PENK;ANO10;TPPP;DRD2;IRX1;IRX2;SLC6A17;BCL11B;LGI2;ISLR2;GFRA1;GRIN2C;BMP7;HS3ST1;SHISA6;APLN;BMP2;PTX3;RGS7BP;VGLL2	TF_Consensus	0.02	4.43	ENCODE_an	SUZ12 CHEA
GATA1 CHEA	13	807	5E-03	2E-01	12.6	GBP6;PLEK;RFX2;AFF3;RAP1GAP;MTSS1;ADAM19;TG;BDH1;SLCO2B1;KIT;ANO10;ACSS1	TF_Consensus	0.02	3.65	ENCODE_an	GATA1 CHEA
ZC3H11A ENCODE	3	129	7E-02	1E+00	9.2	DUSP23;CMSS1;RNF166	TF_Consensus	0.02	3.19	ENCODE_an	ZC3H11A ENCODE
ESR1 CHEA	3	154	1E-01	1E+00	6.5	SH3BP4;GFRA1;BMP7	TF_Consensus	0.02	2.70	ENCODE_an	ESR1 CHEA
Complement and coagulation cascades	5	85	4E-04	4E-02	71.0	C4B;ITGAM;C7;ITGB2;VSIG4	KEGG	0.06	6.15	KEGG_2021	Complement and coagulation cascades
Synthesis and degradation of ketone bodies	1	10	7E-02	5E-01	41.5	BDH1	KEGG	0.10	5.37	KEGG_2021	Synthesis and degradation of ketone bodies
Viral protein interaction with cytokine and cytokine receptor	4	100	6E-03	2E-01	30.5	CCL8;CCR5;CCR2;PF4	KEGG	0.04	4.93	KEGG_2021	Viral protein interaction with cytokine and cytokine receptor
Pertussis	3	76	2E-02	3E-01	23.6	C4B;ITGAM;ITGB2	KEGG	0.04	4.56	KEGG_2021	Pertussis
Extracellular Matrix Organization R-HSA-1474244	10	291	5E-05	2E-02	52.1	ADAM19;MMP12;BMP2;ITGAM;TNXB;LAMB3;ITGB2;FBLN1;BMP7;CTSS	Reactome	0.03	5.70	Reactome_	Extracellular Matrix Organization R-HSA-1474244
Molecules Associated With Elastic Fibres R-HSA-2129379	3	28	1E-03	2E-01	116.9	BMP2;FBLN1;BMP7	Reactome	0.11	6.87	Reactome_	Molecules Associated With Elastic Fibres R-HSA-2129379
Signaling To P38 Via RIT And RIN R-HSA-187706	1	5	4E-02	4E-01	116.9	BCL11B	Reactome	0.20	6.87	Reactome_	Signaling To P38 Via RIT And RIN R-HSA-187706
Abacavir Transmembrane Transport R-HSA-2161517	1	5	4E-02	4E-01	116.9	SLC22A1	Reactome	0.20	6.87	Reactome_	Abacavir Transmembrane Transport R-HSA-2161517
MGS724 Murine age10wk left ventricle male transverse aortic constriction v sham GSE18224 down	18	115	2E-19	2E-16	1261.6	SNED1;ABRACL;THBS2;ALDH1L2;TMPRSS13;MRGPRH;CEP128;TMEM47;RGS2;CENPF;TBC1D4;PHKG1;PENK;ENPP2;TBC1D10C;SLC16A7;ANO10;RGS7BP	SysMyo Muscle	0.16	10.30	SysMyo_Mu	MGS724 Murine age10wk left ventricle male transverse aortic constriction v sham GSE18224 down
MGS177 Murine soleus age8wk mdx v ctl PMID 16261416 up	6	30	6E-08	2E-06	600.7	CCL9;CCL8;LYZ2;CTSS;MPEG1;CCR2	SysMyo Muscle	0.20	9.23	SysMyo_Mu	MGS177 Murine soleus age8wk mdx v ctl PMID 16261416 up
MGS32 Murine extraocular age28d mdx v ctl PMID 12874102 up	2	6	7E-04	6E-03	506.8	CCL9;LYZ1	SysMyo Muscle	0.33	8.99	SysMyo_Mu	MGS32 Murine extraocular age28d mdx v ctl PMID 12874102 up
MGS781 Murine age13wk heart beta1 Gly389 transgenic v WT GSE11887 up	9	69	1E-09	9E-08	450.4	ACTA2;IRX2;RGS2;CCL8;PHKG1;MT1;GRIN2C;TMPRSS13;LRRC52	SysMyo Muscle	0.13	8.82	SysMyo_Mu	MGS781 Murine age13wk heart beta1 Gly389 transgenic v WT GSE11887 up
CREB1 KD MOUSE GSE17478 CREEDSID GENE 2158 DOWN	14	184	6E-11	5E-08	295.9	GBP6;IRX2;BEX1;SLC41A3;DUSP18;CEP128;TMEM47;RGS2;CENPF;TBC1D4;PHKG1;TBC1D10C;SLC16A7;ANO10	TF_Pertubations	0.08	8.21	TF_Pertur	CREB1 KD MOUSE GSE17478 CREEDSID GENE 2158 DOWN
MYC OE MOUSE GSE55272 CREEDSID GENE 1824 DOWN	19	323	2E-12	3E-09	266.3	ITGAM;CLEC12A;HP;CFP;MPEG1;CTSS;C4B;ADGRE1;RGS2;CCDC80;CCL8;GPNMB;ENPP2;SLC16A7;VSIG4;IGFALS;CCR5;FOLR2;CCR2	TF_Pertubations	0.06	8.06	TF_Pertur	MYC OE MOUSE GSE55272 CREEDSID GENE 1824 DOWN
SRF KO MOUSE GSE34545 CREEDSID GENE 2884 UP	18	367	1E-10	8E-08	182.3	GBP6;SNED1;GFPT2;ITGB2;LGI2;DPT;FBLN1;MPEG1;CTSS;HS3ST1;C4B;ADGRE1;CLEC3B;CCL8;PENK;MMRN2;FOLR2;PF4	TF_Pertubations	0.05	7.51	TF_Pertur	SRF KO MOUSE GSE34545 CREEDSID GENE 2884 UP
GATA4 KO MOUSE GSE52317 CREEDSID GENE 356 DOWN	12	231	1E-07	4E-05	131.9	MMP12;RP1;CENPF;ITGAM;PHKG1;KCNIP2;IGFALS;DUSP18;ABCA12;TMPRSS13;CTSC;MTSS1	TF_Pertubations	0.05	7.04	TF_Pertur	GATA4 KO MOUSE GSE52317 CREEDSID GENE 356 DOWN

Supplemental Table 8. Singular enrichment analysis of bulk RNAseq differentially expressed upregulated genes of hearts from 5 MLPKO and 5 cRAD$^{\Delta/\Delta}$-MLPKO 3.5-month old male mice (1 month after cRAD$^{\Delta/\Delta}$ induction). Databases queried included experimental model databases (Muscle Gene Set, TF Pertubations), GO, transcription factors (TF Consensus of ENCODE & CHEA, and TRRUST) and pathway databases (Wiki Pathways, BioPlanet, KEGG). Analysis was performed using Enrichr. The genes columns includes all hits of DE upregulated genes for a given term (p-adj <0.05, $\log_2$ fold-change > 0.5) (DESeq2).

id	term	size	leading edge #	ES	NES	p value	FDR	category
WP295	Electron transport chain	78	61	0.70465	2.6573	<2.2e-16	<2.2e-16	pathway_WikiPathways
GO:0070469	respirasome	73	54	0.70422	2.6142	<2.2e-16	<2.2e-16	geneontology_Cellular_Component_noRedundant
mmu00190	Oxidative phosphorylation	110	74	0.64716	2.5598	<2.2e-16	<2.2e-16	pathway_KEGG
GO:0098798	mitochondrial protein-containing complex	260	139	0.5424	2.4109	<2.2e-16	<2.2e-16	geneontology_Cellular_Component_noRedundant
WP434	TCA cycle	30	27	0.75979	2.3208	<2.2e-16	<2.2e-16	pathway_WikiPathways
WP1248	Oxidative phosphorylation	44	34	0.69088	2.3019	<2.2e-16	<2.2e-16	pathway_WikiPathways
GO:1990204	oxidoreductase complex	96	64	0.59858	2.287	<2.2e-16	<2.2e-16	geneontology_Cellular_Component_noRedundant
mmu05150	Staphylococcus aureus infection	30	23	0.73707	2.2832	<2.2e-16	<2.2e-16	pathway_KEGG
mmu05415	Diabetic cardiomyopathy	177	100	0.53877	2.2775	<2.2e-16	<2.2e-16	pathway_KEGG
GO:0009055	electron transfer activity	37	29	0.67077	2.2161	<2.2e-16	<2.2e-16	geneontology_Molecular_Function_noRedundant
mmu03010	Ribosome	126	83	0.5378	2.2007	<2.2e-16	<2.2e-16	pathway_KEGG
mmu04932	Non-alcoholic fatty liver disease	136	76	0.53532	2.176	<2.2e-16	<2.2e-16	pathway_KEGG
GO:0033865	nucleoside bisphosphate metabolic process	89	49	0.57097	2.1657	<2.2e-16	<2.2e-16	geneontology_Biological_Process_noRedundant
mmu00020	Citrate cycle (TCA cycle)	29	23	0.70523	2.1608	<2.2e-16	<2.2e-16	pathway_KEGG
mmu05208	Chemical carcinogenesis	189	103	0.50702	2.1528	<2.2e-16	<2.2e-16	pathway_KEGG
GO:0010257	NADH dehydrogenase complex assembly	53	31	0.61805	2.1403	<2.2e-16	0.00059294	geneontology_Biological_Process_noRedundant
GO:0070069	cytochrome complex	28	24	0.71901	2.1354	<2.2e-16	<2.2e-16	geneontology_Cellular_Component_noRedundant
GO:0003735	structural constituent of ribosome	145	90	0.51428	2.1254	<2.2e-16	0.00053297	geneontology_Molecular_Function_noRedundant
mmu04610	Complement and coagulation cascades	41	21	0.64549	2.121	<2.2e-16	0.00012931	pathway_KEGG
GO:0001848	complement binding	15	8	0.81602	2.1001	<2.2e-16	0.0010659	geneontology_Molecular_Function_noRedundant
GO:1901568	fatty acid derivative metabolic process	36	18	0.66087	2.0714	<2.2e-16	0.0023718	geneontology_Biological_Process_noRedundant
WP163	Cytoplasmic ribosomal proteins	76	53	0.55457	2.0366	<2.2e-16	0.0026531	pathway_WikiPathways
mmu04061	Viral protein interaction with cytokine and cytokine receptor	40	18	0.61327	2.0006	<2.2e-16	0.0014943	pathway_KEGG
mmu01212	Fatty acid metabolism	52	32	0.57663	1.9995	<2.2e-16	0.0013448	pathway_KEGG
GO:0045239	tricarboxylic acid cycle enzyme complex	20	15	0.7187	1.9874	<2.2e-16	0.0018665	geneontology_Cellular_Component_noRedundant
GO:0006959	humoral immune response	89	35	0.51157	1.9531	<2.2e-16	0.017195	geneontology_Biological_Process_noRedundant
GO:0009897	external side of plasma membrane	239	99	0.44107	1.9455	<2.2e-16	0.0029553	geneontology_Cellular_Component_noRedundant
GO:0072522	purine-containing compound biosynthetic process	197	98	0.44407	1.9185	<2.2e-16	0.022769	geneontology_Biological_Process_noRedundant
GO:0006091	generation of precursor metabolites and energy	380	155	0.40389	1.8883	<2.2e-16	0.027275	geneontology_Biological_Process_noRedundant
GO:0034694	response to prostaglandin	19	8	0.65988	1.8819	<2.2e-16	0.025412	geneontology_Biological_Process_noRedundant
WP2316	PPAR signaling pathway	47	19	0.5579	1.877	<2.2e-16	0.017245	pathway_WikiPathways
WP4348	Eicosanoid metabolism via lipoxygenases LOX	17	8	0.69494	1.8633	0.002004	0.017466	pathway_WikiPathways
GO:1901681	sulfur compound binding	174	56	0.43461	1.8595	<2.2e-16	0.02505	geneontology_Molecular_Function_noRedundant
GO:0019865	immunoglobulin binding	8	6	0.81564	1.851	0.0020408	0.021958	geneontology_Molecular_Function_noRedundant
GO:0016614	oxidoreductase activity, acting on CH-OH group of donors	90	37	0.47868	1.8386	<2.2e-16	0.021141	geneontology_Molecular_Function_noRedundant
GO:0016651	oxidoreductase activity, acting on NAD(P)H	43	25	0.54095	1.8383	0.002045	0.018121	geneontology_Molecular_Function_noRedundant
WP441	Matrix metalloproteinases	18	8	0.67705	1.8359	<2.2e-16	0.020656	pathway_WikiPathways
GO:0043394	proteoglycan binding	28	13	0.60566	1.8296	<2.2e-16	0.017988	geneontology_Molecular_Function_noRedundant
GO:0008217	regulation of blood pressure	129	52	0.44091	1.8247	<2.2e-16	0.041901	geneontology_Biological_Process_noRedundant
GO:0015078	proton transmembrane transporter activity	84	33	0.47848	1.8226	<2.2e-16	0.017647	geneontology_Molecular_Function_noRedundant
WP2271	Macrophage markers	10	6	0.80705	1.8224	0.0039841	0.022054	pathway_WikiPathways
GO:0006790	sulfur compound metabolic process	225	82	0.41534	1.8203	<2.2e-16	0.040083	geneontology_Biological_Process_noRedundant
WP1596	Iron homeostasis	8	5	0.84197	1.819	0.002	0.021078	pathway_WikiPathways
GO:0140375	immune receptor activity	74	33	0.48313	1.8069	<2.2e-16	0.01876	geneontology_Molecular_Function_noRedundant
GO:0070371	ERK1 and ERK2 cascade	234	82	0.41084	1.8051	<2.2e-16	0.04111	geneontology_Biological_Process_noRedundant
WP1269	Fatty acid beta oxidation	30	18	0.55549	1.7213	0.0083333	0.042627	pathway_WikiPathways
GO:0031983	vesicle lumen	8	4	0.77514	1.7196	0.004158	0.03933	geneontology_Cellular_Component_noRedundant
mmu04927	Cortisol synthesis and secretion	48	17	-0.54469	-1.8204	0.0037313	0.046756	pathway_KEGG
GO:0016528	sarcoplasm	86	18	-0.50148	-1.8405	0.0018657	0.026191	geneontology_Cellular_Component_noRedundant
GO:1905368	peptidase complex	108	49	-0.48305	-1.8431	<2.2e-16	0.03351	geneontology_Cellular_Component_noRedundant
mmu05412	Arrhythmogenic right ventricular cardiomyopathy	65	24	-0.53185	-1.8466	0.0018727	0.040014	pathway_KEGG
WP2152	BDNF pathway	14	4	-0.7279	-1.8501	0.0020121	0.045637	pathway_WikiPathways
mmu05414	Dilated cardiomyopathy	82	22	-0.51061	-1.8586	0.0018349	0.045592	pathway_KEGG
GO:0070971	endoplasmic reticulum exit site	25	13	-0.66773	-1.9552	0.0019531	0.0089949	geneontology_Cellular_Component_noRedundant
GO:0043292	contractile fiber	208	56	-0.53819	-2.2385	<2.2e-16	<2.2e-16	geneontology_Cellular_Component_noRedundant

Supplemental Table 9. Gene set enrichment analysis of bulk RNAseq of hearts from 5 MLPKO and 5 cRAD$^{\Delta/\Delta}$-MLPKO 3.5-month old male mice (1 month after cRAD$^{\Delta/\Delta}$ induction). The input genes were ranked by $\log_2$ fold change. WebGestalt was used for the analysis.

Term	hits	size	P.value	Adjusted.P.value	Combined.Score	Genes	database	Gene_Ratio	log2_score	originalDB	full_term
Focal adhesion	12	233	1E-05	7E-03	57.2	SHC4;BLK;LAMC3;PDGFC;BCL2;PAK6;FLNC;THBS1;MYL12A;THBS4;MAPK4;ITGA9	WikiPath & BioPlan	0.05	5.84	BioPlanet	Focal adhesion
BDNF signaling pathway	11	261	2E-04	3E-02	35.4	DUSP4;SLC7A5;NUAK1;EGR3;GDF15;SERPINE1;DPYSL3;HSPB1;CSPG4;FLNC;AMOT	WikiPath & BioPlan	0.04	5.14	BioPlanet	BDNF signaling pathway
TGF-beta regulation of extracellular matrix	18	565	6E-05	2E-02	30.0	TGFB2;TNFRSF12A;SERPINE1;PTN;INHBA;ETV5;SLC7A5;CNN1;RCAN1;ACTA1;UCK2;UCHL1;PTTG1;CILP;MYOT;COL8A2;CRLF1;PFKP	WikiPath & BioPlan	0.03	4.91	BioPlanet	TGF-beta regulation of extracellular matrix
MYOD1 ENCODE	8	166	5E-04	3E-02	34.9	CACNB1;SYNPO2L;ANKRD1;XIRP1;HSPB1;TRIM16;KLHL40;RYR3	TF Consensus	0.05	5.12	ENCODE_an	MYOD1 ENCODE
SUZ12 CHEA	38	1684	2E-05	2E-03	24.6	SLC22A4;SHC4;SLC24A2;NRN1;ABCB4;HR;ADCY1;LRP8;PPM1E;NKD2;ADORA1;KLRG2;FLNC;ADAMTS8;DUSP4;TGFB2;EGR3;PRRX2;SHISA3;SYPL2;GDF6;POU3F1;KLHL40;ADAMTS20;CNKSR2;SH3RF3;GNAO1;TBX15;RBP4;FRAS1;SYT12;DOK5;SLCO2A1;BCL2;PLEKHO1;ASTN2;CRLF1;SNTB1	TF Consensus	0.02	4.62	ENCODE_an	SUZ12 CHEA
skeletal muscle cell differentiation	4	12	7E-06	3E-03	539.4	ANKRD1;ANKRD2;KLHL40;KLHL41	GO BP	0.33	9.08	GO_Biolog	skeletal muscle cell differentiation (GO:0035914)
response to muscle stretch	3	9	1E-04	2E-02	412.3	CSRP3;NPPA;ANKRD1	GO BP	0.33	8.69	GO_Biolog	response to muscle stretch (GO:0035994)
skeletal muscle fiber development	3	12	3E-04	4E-02	246.6	ACTA1;KLHL40;KLHL41	GO BP	0.25	7.95	GO_Biolog	skeletal muscle fiber development (GO:0048741)
skeletal muscle tissue development	6	39	4E-06	3E-03	205.8	ACTA1;CSRP3;ANKRD1;ANKRD2;KLHL40;KLHL41	GO BP	0.15	7.68	GO_Biolog	skeletal muscle tissue development (GO:0007519)
I band	3	15	6E-04	2E-02	169.1	ANKRD1;ANKRD2;KLHL40	GO CC	0.20	7.40	GO_Cellul	I band (GO:0031674)
sarcomere	4	27	2E-04	7E-03	133.3	ACTA1;ANKRD1;ANKRD2;MYH7	GO CC	0.15	7.06	GO_Cellul	sarcomere (GO:0030017)
filamentous actin	3	22	2E-03	3E-02	90.2	DPYSL3;XIRP1;XIRP2	GO CC	0.14	6.50	GO_Cellul	filamentous actin (GO:0031941)
sarcoplasmic reticulum	3	23	2E-03	3E-02	83.9	THBS1;RYR3;THBS4	GO CC	0.13	6.39	GO_Cellul	sarcoplasmic reticulum (GO:0016529)
ECM-receptor interaction	8	88	6E-06	6E-04	110.5	FRAS1;LAMC3;COL4A3;COL9A2;FREM1;THBS1;THBS4;ITGA9	KEGG	0.09	6.79	KEGG_2021	ECM-receptor interaction
Focal adhesion	12	201	3E-06	5E-04	76.0	SHC4;LAMC3;PDGFC;COL4A3;BCL2;PAK6;COL9A2;FLNC;THBS1;MYL12A;THBS4;ITGA9	KEGG	0.06	6.25	KEGG_2021	Focal adhesion
TAC vs Sham Fem 10wk Up	64	183	2E-80	2E-77	12268.0	CLIC5;HSPB6;PTPRN;MEOX1;NPPB;MMP23;FGF6;RNF115;UCHL1;DPYSL3;NPPA;ADAMTSL2;ANKRD1;NUDT18;RRP12;GAS2L3;TNFRSF12A;LMAN1L;SHISA4;KLHL40;ENAH;CNKSR1;ACTA1;SYNPO2L;DCT;MYOT;COL4A3;COL8A2;COL8A1;PLEKHO1;ITGA9;MYH7;HBEGF;SLC22A4;CCDC68;ABCB4;PRUNE2;NLRC3;THBS1;PPM1E;MYL12A;RTN4;THBS4;FSTL3;NUAK1;TSPAN9;NRAP;FLNC;PLET1OS;TGFB2;TMEM62;HSP25-PS1;MLLT11;TTLL7;RCAN1;MFAP4;TBX15;UCK2;CILP;OTULIN;BCL2;FMOD;CRLF1;LRP11	Muscle Gene Set	0.35	13.58	SysMyo_Mu	MGS717 Murine age10wk left ventricle female transverse aortic constriction v sham GSE18224 up
TAC ER-beta KO vs Sham Fem 10wk Up	56	181	2E-66	8E-64	8029.5	HSPB6;PTPRN;SETD7;MEOX1;RND1;NPPB;MMP23;FGF6;UCHL1;DPYSL3;NPPA;ADAMTSL2;ANKRD1;NUDT18;RRP12;GAS2L3;TNFRSF12A;LMAN1L;SHISA4;KLHL40;ENAH;CNKSR1;ACTA1;TUBB2A;CRISPLD1;SYNPO2L;MYOT;COL8A1;PLEKHO1;MYH7;HBEGF;CCDC68;PRUNE2;NLRC3;THBS1;MYL12A;THBS4;FSTL3;NUAK1;TSPAN9;NRAP;TGFB2;PMM1;GDF15;HSP25-PS1;MLLT11;RCAN1;MFAP4;TBX15;UCK2;CILP;OTULIN;MAFK;CRLF1;PFKP;LRP11	Muscle Gene Set	0.31	12.97	SysMyo_Mu	MGS715 Murine age10wk left ventricle female transverse aortic constriction estrogen receptor beta KO v sham GSE18224 up
TAC vs Sham 11wk Up	47	159	2E-54	4E-52	5840.7	CLIC5;HSPB6;SERPINE1;FHL1;NLRC3;MEOX1;MYL12A;RTN4;THBS4;UCHL1;NMRK2;DPYSL3;NPPA;ADAMTSL2;NRAP;ANKRD1;FLNC;PRKAB2;TGFB2;LMAN1L;GDF15;SHISA3;MLLT11;ENAH;TTLL7;CNKSR1;ACTA1;MFAP4;TBX15;SPCS3;UCK2;STAR;SYNPO2L;CILP;OTULIN;WDFY1;NAPEPLD;BCL2;COL8A1;MAFK;PLEKHO1;TRIM16;CRLF1;PFKP;LRP11;ITGA9;MYH7	Muscle Gene Set	0.30	12.51	SysMyo_Mu	MGS731 Murine age11wk myocardium aortic banding failing v sham GSE36074 up
TAC Gata4 Het vs WT Up	54	204	8E-60	2E-57	5725.8	FHL1;MEOX1;AMOT;NPPB;FGF6;RNF115;UCHL1;NMRK2;NUDT18;ANKRD2;RRP12;SCN1B;PRKAB2;LMAN1L;SHISA4;KLHL40;ENAH;CNKSR1;ACTA1;TUBB2A;SYNPO2L;MYOT;COL8A1;HBEGF;ITGA9;MYH7;SLC22A4;CCDC68;PPM1E;RTN4;THBS4;FSTL3;NKD2;NUAK1;FLNC;PLET1OS;MAPK4;TGFB2;GDF15;TMEM62;NEB;HSP25-PS1;MLLT11;SPCS3;RCAN1;MFAP4;UCK2;CILP;OTULIN;BCL2;MAFK;ATP13A3;CRLF1;PFKP	Muscle Gene Set	0.26	12.48	SysMyo_Mu	MGS1503 Murine ventricle transverse aortic constriction Gata4 heterozygotes v WT GSE5500 up
CREB1 KD Mouse Up	51	346	1E-42	3E-39	1897.8	PTPRN;MEOX1;AMOT;NPPB;FGF6;UCHL1;NMRK2;DPYSL3;ADAMTSL2;NPPA;NUDT18;RRP12;SCN1B;SLC38A1;GAS2L3;TNFRSF12A;LMAN1L;RRAS2;SHISA3;ETV5;ENAH;CNKSR1;SLC7A5;ACTA1;SYNPO2L;COL4A3;COL8A1;MYH7;ITGA9;SLC22A4;SHC4;CCDC68;RTN4;THBS4;FSTL3;NUAK1;PDGFC;TGFB2;EGR3;GDF15;TMEM62;RAB27B;RCAN1;MFAP4;CILP;BCL2;LNX1;CSPG4;MAFK;CRLF1;LRP11	TF Pertubations	0.15	10.89	TF_Pertur	CREB1 KD MOUSE GSE17478 CREEDSID GENE 2158 UP
GATA6 KO Mouse Up	32	225	3E-26	3E-23	1003.3	NRN1;ATP8A2;CCDC68;ABCB4;FHL3;HSPB1;NLRC3;LRRC55;THBS1;PPM1E;THBS4;AMOT;NPPB;UCHL1;DPYSL3;NPPA;XIRP2;PTK2B;FLNC;SLC38A1;TGFB2;SHISA4;ADAMTS20;CNKSR1;ACTA1;MFAP4;CILP;MYOT;PLEKHO1;ATP13A3;ASTN2;MYH7	TF Pertubations	0.14	9.97	TF_Pertur	GATA6 KO MOUSE GSE52317 CREEDSID GENE 357 UP
GATA4 KO Mouse Down	24	229	1E-16	6E-14	426.9	SLC22A4;PRKAB2;TGFB2;SERPINE1;PRUNE2;SHISA4;DAPP1;SHISA3;NEB;MEOX1;LRP8;THBS1;CNKSR1;FGF6;CNN1;ACTA1;UCK2;STAR;MYOT;SYNDIG1;PTK2B;ANKRD2;FMOD;MYH7	TF Pertubations	0.10	8.74	TF_Pertur	GATA4 ABLATION MOUSE GSE30314 CREEDSID GENE 838 DOWN
GTF2I KO Mouse Down	23	259	2E-14	7E-12	304.0	PRKAB2;TGFB2;EGR3;TNFRSF12A;CCDC68;SERPINE1;FHL1;INHBA;THBS1;FSTL3;RND1;NPPB;CNN1;RCAN1;ACTA1;MFAP4;NUAK1;COL8A2;ANKRD1;CSPG4;FMOD;CRLF1;HBEGF	TF Pertubations	0.09	8.25	TF_Pertur	GTF2I KO MOUSE GSE48790 CREEDSID GENE 1451 DOWN
GATA4 mouse TRRUST	6	22	1E-07	2E-05	549.0	NPPB;STAR;NPPA;ANKRD1;ADORA1;LRRC10	TF Consensus	0.27	9.10	TRRUST_Tr	GATA4 mouse
NKX2-5 mouse TRRUST	4	25	2E-04	1E-02	151.4	NPPB;NPPA;ADORA1;LRRC10	TF Consensus	0.16	7.24	TRRUST_Tr	NKX2-5 mouse
Cardiomyocyte Signaling Pathways Converging On Titin	4	33	5E-04	4E-02	95.8	CSRP3;FHL1;ANKRD1;ANKRD2	WikiPath & BioPlan	0.12	6.58	WikiPathw	Cardiomyocyte Signaling Pathways Converging On Titin WP5344
Focal Adhesion	11	199	1E-05	4E-03	60.5	BLK;SHC4;LAMC3;PDGFC;BCL2;PAK6;FLNC;THBS1;MYL12A;THBS4;ITGA9	WikiPath & BioPlan	0.06	5.92	WikiPathw	Focal Adhesion WP306
MicroRNAs In Cardiomyocyte Hypertrophy	6	83	3E-04	4E-02	57.1	NPPB;RCAN1;NPPA;NRG1;MAP3K14;MAPK4	WikiPath & BioPlan	0.07	5.84	WikiPathw	MicroRNAs In Cardiomyocyte Hypertrophy WP1544

Supplemental Table 10. Singular enrichment analysis of shared downregulated bulk RNAseq differentially expressed genes between published MLP$^{+/+}$ vs MLPKO[31] and this study's of cRAD$^{\Delta/\Delta}$-MLPKO vs MLPKO. The analysis was performed on genes in both studies that had log_2 FC< -0.5. Databases queried included experimental model databases (Muscle Gene Set, TF Pertubations), GO, transcription factors (TF Consensus of ENCODE & CHEA, and TRRUST) and pathway databases (Wiki Pathways, BioPlanet, KEGG). Analysis was performed using Enrichr. The genes columns includes all hits of DE downregulated genes for a given term (log_2 fold-change < -0.5) (DESeq2).

SEA Enriched Term	Genes (hits)	Curated term
Focal adhesion (Bioplanet)	12	Mechanical force sensing: ECM, Focal adhesion, ECM receptor activity
BDNF signaling pathway	11	Sarcomere & Hypertrophy
TGF-beta reg of ECM	18	Mechanical force sensing: ECM, Focal adhesion, ECM receptor activity
MYOD1 ENCODE	8	Hypertrophy Transcription Factors
SUZ12 CHEA	38	Hypertrophy Transcription Factors
skeletal muscle cell differentiation	4	Sarcomere & Hypertrophy
response to muscle stretch	3	Sarcomere & Hypertrophy
skeletal muscle fiber dev	3	Sarcomere & Hypertrophy
skeletal muscle tissue dev	6	Sarcomere & Hypertrophy
I band	3	Sarcomere & Hypertrophy
sarcomere	4	Sarcomere & Hypertrophy
filamentous actin	3	Sarcomere & Hypertrophy
sarcoplasmic reticulum	3	Sarcoplasmic Reticulum
ECM-receptor interaction	8	Mechanical force sensing: ECM, Focal adhesion, ECM receptor activity
Focal adhesion (KEGG)	12	Mechanical force sensing: ECM, Focal adhesion, ECM receptor activity
TAC vs Sham 10wk Up	64	Genes Up in HF Disease Models
TAC ER-beta KO vs Sham 10wk Up	56	Genes Up in HF Disease Models
TAC vs Sham 11wk Up	47	Genes Up in HF Disease Models
TAC Gata4 Het vs WT Up	54	Genes Up in HF Disease Models
CREB1 KD Up	51	Hypertrophy Transcription Factors
GATA6 KO Up	32	Hypertrophy Transcription Factors
GATA4 KO Down	24	Hypertrophy Transcription Factors
GTF2I KO Down	23	Hypertrophy Transcription Factors
GATA4 TRRUST	6	Hypertrophy Transcription Factors
NKX2-5 TRRUST	4	Hypertrophy Transcription Factors
Cardiomyocyte Signaling Pathways Converging On Titin	4	Sarcomere & Hypertrophy
Focal Adhesion (WikiPathways)	11	Mechanical force sensing: ECM, Focal adhesion, ECM receptor activity
MicroRNAs In Cardiomyocyte Hypertrophy	6	Sarcomere & Hypertrophy

Supplemental Table 11. Singular enrichment analysis terms and their curated category grouping of shared downregulated bulk RNAseq differentially expressed genes between published MLP$^{+/+}$ vs MLPKO[31] and this study's cRAD$^{\Delta/\Delta}$-MLPKO vs MLPKO. These SEA terms are from Supp. Table 10, and this table represents the grouping terms used in the alluvial diagram in Figure 7F.
