# Supplementary material for: RRAD-reduction reveals efficacy of targeting L-type calcium channel regulation for treatment of heart failure
Source: Cardiovasc Res. 2025 Oct 1;121(14):2204–21. doi: 10.1093/cvr/cvaf169 (PMC12638741; doi:10.1093/cvr/cvaf169)
Supplement: cvaf169_Supplementary_Data [file cvaf169_supplementary_data.zip › Supplementary Text Materials and Methods v4 June Rev1.docx]

**Supplemental Materials and Methods for:**

**RRAD-reduction reveals efficacy of targeting L-type calcium channel regulation for treatment of heart failure**

Elmore et al.

**Methods**

All experimental procedures and protocols were approved by the Animal Care and Use Committee of the University of Kentucky and conformed to the National Institute of Health “Guide for the Care and Use of Laboratory Animals.” Researchers were blinded to genotype during experiments and subsequent analysis.

*Animal model*. All mouse lines used in this work have been previously published^1,2^. Muscle lim protein knocked out (MLPKO) mice were crossed with floxed RAD (RAD^fl/fl^) mice to yield MLP^-/-^, RAD^fl/fl^. To allow inducible, cardiomyocyte-specific deletion of RAD*,* mice were then crossed with mice containing a tamoxifen-inducible Cre recombinase under control of a myosin heavy chain 6 promoter (MYH6-MerCreMer) transgene. This cross yielded MLPKO-RAD^fl/fl^ and MYH6-MerCreMer-MLPKO-RAD^fl/fl^. All mice were administered a single dose of tamoxifen (100 mg/kg intraperitoneal) at 10 weeks (2.5 months).

*Longitudinal echocardiography*. Transthoracic echocardiography was performed at 2.5, 3.5, and 4.5 months of age using the Vevo 3100 high-resolution imaging system equipped with a MX550D (25-55 MHz) linear transducer (FujiFilm, VisualSonics, Inc., Toronto, Canada). Mice were first anesthetized with 2% isoflurane in an induction chamber, chest hair was removed prior to imaging, then a nose cone was placed with inhaled isoflurane, (0.5–1% + 0.5-1.0 L/min 100% O_2_) to maintain a light anesthesia level, with heart rate (350 –500 beats per minute) and core temperature (37°C) continuously monitored and maintained by a heated platform. Male and female mice were evaluated. The heart was visualized from the modified parasternal long axis and short axis views. The left ventricular dimensions and calculated left ventricular EF were measured from the short axis M-mode view ^3^. For each observation, the echocardiogram was traced in triplicate and averaged.

*Histology and gravimetrics*. At 1- and 2-months-post-tamoxifen, mice were anesthetized with ketamine and xylazine (90+10 mg/kg intraperitoneal) before cervical dislocation and heart excision. Heart and wet lung weights were measured. Hearts were perfused with phosphate-buffered saline, fixed overnight in 10% formalin in phosphate-buffered saline, and dehydrated through an ethanol series. Gross heart images were acquired with a Nikon stereo microscope SMZ25 equipped with a Nikon DS-Ri2 and Nikon P2-SHR Plan Apo 0.5x 0.078 NA. Fixed hearts were halved along the short axis with papillary muscle visible, paraffin embedded and sectioned at 5 μm. For cross-sectional area measurements of cardiomyocytes, sections were initially washed with 1x phosphate-buffered saline three times for 3 minutes, blocked for 30 min at room temperature in 3% bovine serum albumin in phosphate-buffered saline, and washed again. Sections were then incubated with conjugated 0.1 mg/ml wheat germ agglutinin-Alexa Flour 488 (Sigma L4895) in phosphate-buffered saline for 15 min at room temperature. Sections were then washed again with phosphate-buffered saline before cover slipping with Vectashield with DAPI (Vector Labs H-1200). A Nikon (Tokyo, Japan) Eclipse E600 equipped with a DS-Fi3, 40X Plan Apo 0.95NA, and mercury lamp was used to acquire >30 technical replicate images per heart (2 sections per heart) of the interventricular septum and left ventricular free wall. Regions were only selected in which cardiomyocytes were in cross-section (circular) and not longitudinal. Cross-sectional area of cardiomyocytes was calculated via an adapted Myovision 2.0 that was trained on cardiomyocytes. A training dataset of 1280 annotated images of WGA-stained cardiomyocytes were used in training a neural network model based on the U-net architecture^4^ in a similar approach as previously done for skeletal muscle cross-sectional images^5^. Image data augmentation was used during model training (10 epochs of 300 steps each) to minimize overfitting due to limited number of annotated images. Image augmentation included random rotations, horizontal and vertical translations, shearing, and zoom. The finalized model was validated by comparing to an additional set of 31 manually quantified images, achieving high degrees of correlation in terms of both average cross-sectional area (slope = 0.97, R^2^ = 0.99) and number of cells per image (slope = 0.94, R^2^ = 0.99). For whole heart section images, slides were scanned on a AxioScan.Z1 (Zeiss, Germany). WGA-488 and Picrosirius Red stained slides were imaged with a 20x objective (Plan-Apochromat 0.8NA and N-Achroplan 0.45NA Pol respectively, Zeiss). Images were captured with a Hamamatsu Orca Flash with 16-bit depth, using identical light source intensity and exposure times. Stitching was performed in Zen Blue (Zeiss). For fibrosis quantification, 4 blinded researchers observed pyramid images of each section, including the entire section and 20X magnification of the interventricular septum and left ventricular free wall, and scored each technical replicate 1-5 (2 sections per mouse).

*Immunoblotting*. At 2.5-months of age and 1-month-post-tamoxifen, mice were anesthetized with ketamine and xylazine (90+10 mg/kg intraperitoneal) before cervical dislocation and heart excision. Freshly excised hearts were flash-frozen upon harvest, and tissue was pulverized before sonification in RIPA Lysis and Extraction Buffer (25 mM Tris, 150 mM Sodium Chloride, 1% NP-40, 1% Sodium Deoxycholate, 0.1% SDS, pH 7.6), HaltTM Protease Inhibitor Cocktail 100X solution (Thermo-scientific REF: 1860932 LOT: Y1370267), HaltTM Phosphatase Inhibitor Cocktail 100X solution (Thermo-Scientific REF: 78420 LOT: YH371140). Lysates were centrifuged at 10,000 rpm, and the protein concentration of supernatants was measured. The lysates were analysed on 4-20% SDS PAGE gels. Immunoblotting was performed with anti-Rad (1:1000, EB11419, Everest Biotech, LOT: E020719) in 3% BSA in PBS. Protein detection was performed using luminol/enhancer solution: peroxide solution (BIO-RAD). Protein quantification for detected proteins was determined through Bio-Rad Image lab software and quantified relative to total protein for each respective lane (stain-free total protein measurement).

*Cardiac magnetic resonance* (CMR) imaging was performed on a 7T system (ClinScan, Bruker) when the animals were 4.5 months old (2-months after tamoxifen). A cardiac and respiratory self-gated ultrashort echo time sequence was used with the following parameters: 15 frames per cardiac cycle, field of view 25mm x 25mm, pixel size 0.13mm, slice thickness 1mm, TE: 0.36ms, TR 9ms, flip angle 10. Five short-axis and 3 long-axis images were acquired. Ventricular volumes and feature-tracking analysis was performed by a level-3 trained CMR reader (CMR42 version 5.14.2, Circle Cardiovascular Imaging, Inc., Calgary, Canada). Circumferential, radial, and longitudinal components of strain were each calculated by averaging all 16 AHA segments at end-systole (i.e., the global average over the entire LV).

*Ventricular Myocyte Isolation*. Ventricular cardiomyocytes were prepared as previously described.^6^ Prior to heart excision mice were anesthetized with ketamine + xylazine. Hearts were excised from adult mice 1-2 months post-tamoxifen (2.5-3.5 months of age) and immediately perfused on a Langendorff apparatus with a high-potassium Tyrode buffer and then digested with 5 to 7 mg liberase (Roche). After digestion, atria were removed, and ventricular myocytes were mechanically dispersed. Calcium concentrations were gradually restored to physiological levels in a stepwise fashion, and only healthy quiescent ventricular myocytes were used for electrophysiological analysis within 8 hours.

*Electrophysiological Recordings.* I_Ca,L_ was recorded in the whole-cell configuration of the patch-clamp technique as previously described.^6^ All recordings were performed at room temperature (20° to 22° C). Borosilicate glass pipettes fire-polished to a resistance of 800-3000 kΩ were filled with a solution consisting of (in mmol/L) 125 Cs-methano-sulfonate, 15 TEA-Cl, 1 MgCl_2_, 10 EGTA, and 5 HEPES, 5 Mg-ATP, 5 phosphocreatine, pH 7.2. Physiological Tyrode’s bath solution contained (in mmol/L) 140 NaCl, 5.4 KCl, 1.2 KH_2_PO_4_, 5 HEPES, 5.55 glucose, 1 MgCl_2_, 1.8 CaCl_2_, pH 7.4. After whole-cell access was achieved, zero sodium bath solution was introduced into the chamber consisting of: (in mmol/L) 150 NMDG, 2.5 CaCl_2_, 1 MgCl_2_, 10 glucose, 10 HEPES, 0.0313 tetrodotoxin citrate, 5 4-AP, pH 7.2. L-type calcium currents were evoked by 300 ms depolarizing voltage steps starting from V_rest_ of -80 mV in +5 mV increments up to +40 mV at 5 s intervals. Currents were sampled at 10 kHz. A low-pass filter was applied using an Axopatch 200B (Molecular Devices, San Jose, Ca, USA), digitized with an Axon Digidata 1550B (Molecular Devices), acquired with Clampex 11.2 (Molecular Devices). Clampfit 11.2 (Molecular Devices) was used for analysis. Activation voltage dependence parameters were obtained by fitting the current-voltage slope conductance transform to a Boltzmann distribution of the form G(V)=G_max_/[1+exp(V½/k)], where G_max_ is the maximal conductance and V½ is the activation midpoint. For current decay kinetics, traces were normalized to peak I_Ca,L_, and current 30 ms and 150 ms after peak was normalized to maximum current for each trace.

Surface ECGs were recorded in isoflurane anesthetized mice using the B/35 and BioAmp (ADInstruments). Recordings sampled at 2kHz were analyzed offline with Lab Chart 7 Pro (ADInstruments) for RR, P-duration, PR, QRS, QT and JT intervals.

*Calcium imaging*. Isolated ventricular cardiomyocytes from 1–2-month post-tamoxifen murine hearts were loaded with Fura-2-AM (Invitrogen F1221, Waltham, Ma, USA) to record Ca^2+^ transients, and paced at room temperature at 1 Hz with field stimulation in physiological Tyrode’s (described above), following >2 min of 1 Hz to induce steady state. A subset of cells underwent a train of frequencies in the following order: 1 Hz (60 seconds), 2 Hz (20 s), 3 Hz (15 s), 0.1 Hz (60 s), 0.5 Hz (30s), 1 Hz (30 s). Sarcomere dynamics were simultaneously recorded via fast Fourier transform analysis of visible striations. F_340/380_ was corrected for background fluorescence for each cell. Data was recorded utilizing an Eclipse TE200 (Nikon, Tokyo, Japan), a Nikon oil-immersion plan flour 40X/1.30 NA objective, a Cairn (Kent, UK) arc lamp, IonOptix (Westwood, Ma, USA) MyoCam-S3, MyoPacer Field Stimulator, Fluorescence System Interface, and Hyperswitch and analyzed via IonWixard v7.5.3.165.

For local Ca^2+^ transients, dispersed cardiomyocytes were loaded with 10 µM Fluo-4-AM (Invitrogen F14201) for 15 minutes and washed twice. Cells were imaged on glass (#1.5) bottom dishes at room temperature with a Nikon AX-R confocal microscope (Nikon, Tokyo, Japan) with a Nikon oil-immersion Plan Apo 60X/1.42 NA using an excitation wavelength of 488 nm. Cells were field stimulated at 1 Hz for >3 minutes with an IonOptix MyoPacer Field Stimulator to induce steady state before imaging for 10 seconds at 965 lines per second. Spark and wave events were manually quantified by a blinded observer within the 10 seconds of field stimulation.

**Human hearts**

Donor hearts not utilized for cardiac transplantation were obtained through the United Network for Organ Sharing (UNOS). The human hearts were acquired following the approval of the institutional regulatory board at Baylor College of Medicine. The human hearts were classified according to the following inclusion and exclusion criteria: 1- Normal healthy donors: Our general inclusion criteria are: 1) age greater than or equal to 50, 2) next of kin informed consent, and 3) ejection fraction >55%.  Our exclusion criteria are: 1) history of myocardial infarction, 2) pregnant women, 3) decompensated heart failure, 4) patients with severe kidney disease, 5) asthma or severe chronic lung disease, 6) cardiac pacemaker or implantable defibrillator, 7) cerebral aneurysm clip, 8) neural stimulator, and 9) history of diabetes. 2- HFrEF donors: Our general inclusion criteria are: 1) age greater than or equal to 50, 2) next of kin informed consent, 3) ejection fraction <40%, and 4) history of myocardial infarction.  5) patients with a history of at least one of these conditions, chronic kidney disease, hypertension, or diabetes. Our exclusion criteria are: 1) pregnant women, 2) asthma or severe chronic lung disease, 3) cerebral aneurysm clip, and 4) neural stimulator.

*Human Heart slicing.* Slicing and culturing of 300 μm thick heart tissue slices were established in our lab and performed as previously described.^7,8^ Hearts were placed in a sterile glass container with cardioplegia solution and then the left ventricle was cut into 1-2 cm^3^ blocks. Each heart tissue cube was placed on a 4% agar bed on top of the specimen holder with the epicardium glued to the agar bed using Histoacryl Blue tissue glue and the endocardium facing up. The tissue block holder was placed on the cutting chamber of a Vibrating Microtome 700SMZ (Campden Instruments). The cutting chamber was filled with cold (4°C) oxygenated modified Tyrode’s solution (Tyrode’s cutting solution, NaCl 140mM; KCl 6mM; glucose 10mM; HEPES 10mM; MgCl_2_ 1mM; CaCl_2_ 1.8mM; 2,3-butanedione monoxime (BDM) 10mM; pH 7.4). To limit cardiomyocyte damage while slicing, the vibrating microtome’s z-axis vibration was calibrated with the ceramic cutting blade to <0.5μm. Prior to slicing the vibrating microtome was pre-sets to 300μm slice thickness, 0.03 mm/s advance speed, and 80 Hz vibration frequency at 2mm horizontal vibration amplitude. Following slicing, each slice was transferred immediately to 100μm nylon mesh cell strainers immersed in oxygenated washout Tyrode’s solution at room temperature (NaCl 140mM; KCl 4.5mM; glucose 10mM; HEPES 10mM; MgCl_2_ 1mM; CaCl_2_ 1.8mM; 2x Antibiotic-Antimycotic; pH 7.4) and a metal washer was placed on the top of the slice to prevent wrinkling. The heart slices were kept in the Tyrode’s washing solution for at least 20 minutes to wash out the BDM and warm the tissue to room temperature.

*Human Heart slice culture.* For stimulated culture, slices were glued at each end into sterilized polyurethane printer 6mm wide printer timing belt with metal wires embedded (Uxcell) using histoacryl blue tissue glue. Then the supported heart slices were transferred into 6 well plates containing 6ml of medium in each well (Medium 199, 1x ITS Supplement, 10% FBS, 5ng/ml VEGF, 10ng/ml FGF-basic, and 2x Antibiotic-Antimycotic). Then a C-Dish top with graphite electrodes (Ionoptix) was placed on the top of the 6 well plate and connected to the C-Pace-EM system (Ionoptix), and stimulated at 10V, 1.2Hz. The plates were placed in the incubator at 37°C with humidified air and 5% CO_2_. Media was changed three times/day with preoxygenated media. The C-Dish top with the graphite electrodes was replaced every day to avoid release of toxic carbon into the medium.

*Human heart slice calcium-transient assessment***.** Heart slices were loaded with Fluo-4 for 30 min at room temperature before being transferred to the superfusion chamber. The loading solution contained a 1:10 mixture of 5 mM Fluo-4 AM in dry DMSO and PowerloadTM concentrate (Invitrogen), which was diluted 100-fold into extracellular Tyrode’s solution (NaCl 140mM; KCl 4.5mM; glucose 10mM; HEPES 10mM; MgCl_2_ 1mM; CaCl_2_ 1.8mM; 2x Antibiotic-Antimycotic; pH 7.4). An additional 20 minutes was allowed for de-esterification before recordings were taken. Contractions and calcium transients were evoked by applying voltage pulses at 1 Hz between platinum wires placed on either side of the heart slice and connected to a field stimulator (IonOptix, Myopacer). Fluo-4 fluorescence transients were recorded via a standard filter set (#49011 ET, Chroma Technology). Resting fluorescence was recorded after cessation of pacing, and background light was obtained after removing the heart slice from the field of view at the end of the experiment. All analyses of calcium transients were based on calcium transients recorded from single cardiomyocytes within the heart slice and the calcium transient amplitude was assessed as the average of 10 consecutive beats for each cardiomyocyte. For b-adrenergic stimulation,1μM isoproterenol was used.

*Human heart slice contractile force assessment.* For contractile force measurement, middle strips of the heart slice were assessed using the Dual-Mode Muscle Lever System (300C-LR, Aurora Scientific Inc, Aurora, Canada). The heart slice strip was kept in Krebs Ringer solution gassed with a mixture of 95% O_2_ and 5% CO_2_ throughout the procedure. The heart slice was tied to a metal pin from one end, and the other end was attached to the force transducer with 4-0 silk suture. The heart slice was positioned between two platinum electrodes to provide electrical stimulation using 701C stimulator (Aurora Scientific Inc.). To find optimal length for isometric contraction, the heart slice strip was slowly stretched until passive force was ~30 mN. The preparation was then warmed to 37°C, allowing 10 min for thermo-equilibration, before measurements of contractile properties. We measured isometric specific twitch force at 1Hz at tetanic electrical stimulations (150 Hz, 800-1000 mA, 0.25 ms pulse). The contractile properties of the same heart slice were assessed basally or after addition of 1µM isoproterenol in the myobath to evaluate the inotropic response of the heart slice to b-adrenergic stimulation. All data were recorded and analyzed using commercial software (DMCv5.5 and DMAv5.3, Aurora Scientific). Force was normalized to cross-sectional area (mN/cm^2^); to estimate the heart slice cross sectional area, heart slice strip weight (g) was divided by the slice length (cm) multiplied by the muscle density (1.06 g/cm3).

*Human heart tissue for protein and RNA*. All procedures were approved by the University of Kentucky Institutional Review Board (IRB# 46103), with informed consent from subjects or their legally authorized representatives. Details of the sample procurement method have been published previously.^9^ Hearts used in this study included those from non-failing organ donors (n=10) and hearts meeting the criteria for dilated cardiomyopathy (DCM; n = 10). Our protocol permits the collection of myocardial samples from organ donors when the heart is not suitable for transplant. A heart may be deemed unsuitable for transplant for various reasons that don’t necessarily include heart disease, including age, viral infections, compatibility issues with the recipient, or size mismatch. Since it is unethical to obtain samples from completely healthy individuals, myocardium from organ donors is considered the gold standard for such research. Myocardial samples from patients fit the diagnostic criteria for DCM, which includes no known history of ischemic events, left ventricular ejection fraction (EF) less than 45%, and a left ventricular internal diameter during diastole (LVIDd) greater than 60 mm (males) and 54 mm (females)^10^.

To calculate the relative expression of *RRAD*, the raw band intensity of *RRAD* for each patient sample was first normalized to the raw band intensity of the loading control sample (lane 2). This value was then further normalized to Actin, which was calculated by dividing the raw band intensity of Actin for the patient sample by the raw band intensity of Actin from the loading control. Normalization to Actin accounted for variations in loading and total protein abundance. Data were analyzed using one-way ANOVA with disease as a fixed effect, and significance was set at p < 0.05. Each patient is represented as an opaque circle.

*Bulk RNAseq.* RNA was extracted from rapid-freeze clamped apical halves of cardiac ventricle (N=5 per group). The manufacturers protocol Qiagen RNeasy Fibrous Tissue Mini Kit (50) (Qiagen 74704, Venlo, Netherlands) was followed. RNA with RIN>9 was submitted to Novogene (Sacramento, CA) for sequencing. mRNA enrichment was performed using polyA selection (Kit) and prepared using a library kit. Each FASTQ file was processed using FastQC to assess the quality of the reads. Subsequently, the reads were aligned to the GRCm39 genome assembly using the STAR alignment software. The total successfully assigned alignments ranged from 64% to 74%. Raw counts were calculated with the featureCounts package and pre-filtered excluding non-annotated/predicted genes Gm or Rik, and genes that did not have >10 expression in at least 6 of 10 mice. Big Omics v3.4.4^11^ (Lugano, Switzerland) was used for differential gene expression analysis (DESeq2) with a false discovery rate (FDR) of 0.05 (adj. p < 0.05), principal component analysis, and unsupervised hierarchal clustering. Singular enrichment analysis was performed using Enrichr^12-14^ using an input of significant genes up or downregulated log_2_ fold change 0.5. Gene set enrichment analysis was performed using WebGestalt^15^ using all genes (12,452) ranked based on log_2_ fold change. For the comparative analyses with the MLP^+/+^ vs MLPKO study^16^ the published data was downloaded from the Gene Expression Omnibus database (GSE225008).

*Statistical analysis.* In figures, data are presented as the mean ± standard error of the mean unless represented by a box and whisker plot in which case the medians (line) and min to max (whiskers) are reported. Linear mixed models were used in experiments that had a hierarchical/nested structure of cells being sampled from the same mouse and treated mice as a random factor with genotype as a fixed factor. Normality and equality of variances were assessed visually and via normality (Shapiro-Wilk) and variance (Levene’s) tests. The p-values of pairwise comparisons were corrected for multiple comparisons using Tukey’s and Holm’s tests. The means, their 95% confidence intervals, and sample sizes are reported in the figure legends. In experiments that did not have nested structure, parametric two sample t-tests or nonparametric Mann Whitney U-test was used for comparisons of two groups. A paired t-test was used for comparing the before and after condition in the same sample. A repeated measures ANOVA was used for a subset of cells exposed to a train of stimulus frequencies (1, 2, 3, 0.1 Hz) since each cell was repeatedly measured in the recording. For all statistical tests, alpha was 0.05. Statistical analysis and data visualization was performed using GraphPad Prism 10.2.3 (Boston, Ma, USA), JASP 0.19.0^17^, R Statistical Software (R v4.3.2 and RStudio v2023.12.0; Vienna, Austria)^18^ and the following R packages: data.table, dplyr, extrafont, forcats, ggbeeswarm, gghalves, ggplot2, knitr, lubridate, openslsx, PupillometryR, purrr, RColorBrewer, readr, readxl, rlang, stringr, tibble, tidyr, tidyverse, and writexl^19-38^.

**References – Supplemental Text**

1. Arber S, Hunter JJ, Ross J, Hongo M, Sansig G, Borg J, Perriard J-C, Chien KR, Caroni P. MLP-Deficient Mice Exhibit a Disruption of Cardiac Cytoarchitectural Organization, Dilated Cardiomyopathy, and Heart Failure. *Cell*. 1997;88:393-403. doi: <https://doi.org/10.1016/S0092-8674(00)81878-4>

2. Ahern BM, Levitan BM, Veeranki S, Shah M, Ali N, Sebastian A, Su W, Gong MC, Li J, Stelzer JE, et al. Myocardial-restricted ablation of the GTPase RAD results in a pro-adaptive heart response in mice. *J Biol Chem*. 2019;294:10913-10927. doi: 10.1074/jbc.RA119.008782

3. Zacchigna S, Paldino A, Falcão-Pires I, Daskalopoulos EP, Dal Ferro M, Vodret S, Lesizza P, Cannatà A, Miranda-Silva D, Lourenço AP, et al. Towards standardization of echocardiography for the evaluation of left ventricular function in adult rodents: a position paper of the ESC Working Group on Myocardial Function. *Cardiovasc Res*. 2021;117:43-59. doi: 10.1093/cvr/cvaa110

4. Ronneberger O, Fischer P, Brox T. U-net: Convolutional networks for biomedical image segmentation. Paper/Poster presented at: International Conference on Medical image computing and computer-assisted intervention; 2015;

5. Viggars MR, Wen Y, Peterson CA, Jarvis JC. Automated cross-sectional analysis of trained, severely atrophied, and recovering rat skeletal muscles using MyoVision 2.0. *J Appl Physiol (1985)*. 2022;132:593-610. doi: 10.1152/japplphysiol.00491.2021

6. Magyar J, Kiper CE, Sievert G, Cai W, Shi G-X, Crump SM, Li L, Niederer S, Smith N, Andres DA. Rem-GTPase regulates cardiac myocyte L-type calcium current. *Channels*. 2012;6:166-173.

7. Ou Q, Abouleisa RRE, Tang XL, Juhardeen HR, Meki MH, Miller JM, Giridharan G, El-Baz A, Bolli R, Mohamed TMA. Slicing and Culturing Pig Hearts under Physiological Conditions. *J Vis Exp*. 2020. doi: 10.3791/60913

8. Ou Q, Jacobson Z, Abouleisa RRE, Tang XL, Hindi SM, Kumar A, Ivey KN, Giridharan G, El-Baz A, Brittian K, et al. Physiological Biomimetic Culture System for Pig and Human Heart Slices. *Circ Res*. 2019;125:628-642. doi: 10.1161/circresaha.119.314996

9. Blair CA, Haynes P, Campbell SG, Chung C, Mitov MI, Dennis D, Bonnell MR, Hoopes CW, Guglin M, Campbell KS. A Protocol for Collecting Human Cardiac Tissue for Research. *Vad j*. 2016;2. doi: 10.13023/vad.2016.12

10. Harkness A, Ring L, Augustine DX, Oxborough D, Robinson S, Sharma V. Normal reference intervals for cardiac dimensions and function for use in echocardiographic practice: a guideline from the British Society of Echocardiography. *Echo Res Pract*. 2020;7:G1-g18. doi: 10.1530/erp-19-0050

11. Akhmedov M, Martinelli A, Geiger R, Kwee I. Omics Playground: a comprehensive self-service platform for visualization, analytics and exploration of Big Omics Data. *NAR Genomics and Bioinformatics*. 2019;2. doi: 10.1093/nargab/lqz019

12. Chen EY, Tan CM, Kou Y, Duan Q, Wang Z, Meirelles GV, Clark NR, Ma'ayan A. Enrichr: interactive and collaborative HTML5 gene list enrichment analysis tool. *BMC Bioinformatics*. 2013;14:128. doi: 10.1186/1471-2105-14-128

13. Kuleshov MV, Jones MR, Rouillard AD, Fernandez NF, Duan Q, Wang Z, Koplev S, Jenkins SL, Jagodnik KM, Lachmann A, et al. Enrichr: a comprehensive gene set enrichment analysis web server 2016 update. *Nucleic Acids Res*. 2016;44:W90-97. doi: 10.1093/nar/gkw377

14. Xie Z, Bailey A, Kuleshov MV, Clarke DJB, Evangelista JE, Jenkins SL, Lachmann A, Wojciechowicz ML, Kropiwnicki E, Jagodnik KM, et al. Gene Set Knowledge Discovery with Enrichr. *Current Protocols*. 2021;1:e90. doi: <https://doi.org/10.1002/cpz1.90>

15. Elizarraras JM, Liao Y, Shi Z, Zhu Q, Pico Alexander R, Zhang B. WebGestalt 2024: faster gene set analysis and new support for metabolomics and multi-omics. *Nucleic Acids Research*. 2024;52:W415-W421. doi: 10.1093/nar/gkae456

16. Holmes JB, Lemieux ME, Stelzer JE. Torsional and strain dysfunction precede overt heart failure in a mouse model of dilated cardiomyopathy pathogenesis. *Am J Physiol Heart Circ Physiol*. 2023;325:H449-h467. doi: 10.1152/ajpheart.00130.2023

17. JASP-Team. *JASP (Version 0.19.0)[Computer software]*.

18. R-Core-Team. *R: A Language and Environment for Statistical Computing;* Vienna, Austria: 2023.

19. Xie Y. *knitr: A Comprehensive Tool for Reproducible Research in R;* Chapman and Hall/CRC; 2014.

20. Wickham H, Averick M, Bryan J, Chang W, McGowan LDa, François R, Grolemund G, Hayes A, Henry L, Hester J, et al. Welcome to the tidyverse. *Journal of Open Source Software*. 2019;4:1686. doi: 10.21105/joss.01686

21. Forbes SH. PupillometryR: An R package for preparing and analysing pupillometry data. *Journal of Open Source Software*. 2020;5:2285. doi: 10.21105/joss.02285

22. Neuwirth E. RColorBrewer: ColorBrewer Palettes. 2022.

23. Chang W. extrafont: Tools for Using Fonts. 2023.

24. Clarke E, Sherrill-Mix S, Dawson C. ggbeeswarm: Categorical Scatter (Violin Point) Plots. 2023.

25. Forbes S. PupillometryR: A Unified Pipeline for Pupillometry Data. 2023.

26. Müller K, Wickham H. tibble: Simple Data Frames. 2023.

27. Schauberger P, Walker A. openxlsx: Read, Write and Edit xlsx Files. 2023.

28. Spinu V, Grolemund G, Wickham H. lubridate: Make Dealing with Dates a Little Easier. 2023.

29. Wickham H. forcats: Tools for Working with Categorical Variables (Factors). 2023.

30. Wickham H. stringr: Simple, Consistent Wrappers for Common String Operations. 2023.

31. Wickham H, Bryan J. readxl: Read Excel Files. 2023.

32. Wickham H, François R, Henry L, Müller K, Vaughan D. dplyr: A Grammar of Data Manipulation. 2023.

33. Wickham H, Henry L. purrr: Functional Programming Tools. 2023.

34. Barrett T, Dowle M, Srinivasan A, Gorecki J, Chirico M, Hocking T. data.table: Extension of `data.frame`. 2024.

35. Henry L, Wickham H. rlang: Functions for Base Types and Core R and Tidyverse Features. 2024.

36. Wickham H, Chang W, Henry L, Pedersen TL, Takahashi K, Wilke C, Woo K, Yutani H, Dunnington D, van den Brand T. ggplot2: Create Elegant Data Visualisations Using the Grammar of Graphics. 2024.

37. Wickham H, Hester J, Bryan J. readr: Read Rectangular Text Data. 2024.

38. Wickham H, Vaughan D, Girlich M. *tidyr: Tidy Messy Data;* 2024.
